# Alpha-2-macroglobulin is involved in the occurrence of early-onset pre-eclampsia via its negative impact on uterine spiral artery remodeling and placental angiogenesis

**DOI:** 10.1186/s12916-023-02807-9

**Published:** 2023-03-09

**Authors:** Jingyun Wang, Ping Zhang, Mengyuan Liu, Zhengrui Huang, Xiaofeng Yang, Yuzhen Ding, Jia Liu, Xin Cheng, Shujie Xu, Meiyao He, Fengxiang Zhang, Guang Wang, Ruiman Li, Xuesong Yang

**Affiliations:** 1 Department of Gynaecology and Obstetrics, The First Affiliate Hospital of Jinan University, Jinan University, No.613 Huangpu Road West, Guangzhou, 510632 China; 2 International Joint Laboratory for Embryonic Development & Prenatal Medicine, Division of Histology and Embryology, Medical College, Jinan University, Guangzhou, 510632 China; 3 Fifth Department of Medicine (Nephrology/Endocrinology/Rheumatology/Pneumology), University Medical Centre Mannheim, University of Heidelberg, Mannheim, Germany; 4 Division of Histology & Embryology, Key Laboratory for Regenerative Medicine of the Ministry of Education, Jinan University, No.601 Huangpu Road West, Guangzhou, 510632 China

**Keywords:** A2M, PE, TGFβ1, Uterine spiral artery remodeling, Placental vascularization

## Abstract

**Background:**

Pre-eclampsia (PE) is one of the leading causes of maternal and fetal morbidity/mortality during pregnancy, and alpha-2-macroglobulin (A2M) is associated with inflammatory signaling; however, the pathophysiological mechanism by which A2M is involved in PE development is not yet understood.

**Methods:**

Human placenta samples, serum, and corresponding clinical data of the participants were collected to study the pathophysiologic mechanism underlying PE. Pregnant Sprague–Dawley rats were intravenously injected with an adenovirus vector carrying A2M via the tail vein on gestational day (GD) 8.5. Human umbilical artery smooth muscle cells (HUASMCs), human umbilical vein endothelial cells (HUVECs), and HTR-8/SVneo cells were transfected with A2M-expressing adenovirus vectors.

**Results:**

In this study, we demonstrated that A2M levels were significantly increased in PE patient serum, uterine spiral arteries, and feto-placental vasculature. The A2M-overexpression rat model closely mimicked the characteristics of PE (i.e., hypertension in mid-to-late gestation, histological and ultrastructural signs of renal damage, proteinuria, and fetal growth restriction). Compared to the normal group, A2M overexpression significantly enhanced uterine artery vascular resistance and impaired uterine spiral artery remodeling in both pregnant women with early-onset PE and in pregnant rats. We found that A2M overexpression was positively associated with HUASMC proliferation and negatively correlated with cell apoptosis. In addition, the results demonstrated that transforming growth factor beta 1 (TGFβ1) signaling regulated the effects of A2M on vascular muscle cell proliferation described above. Meanwhile, A2M overexpression regressed rat placental vascularization and reduced the expression of angiogenesis-related genes. In addition, A2M overexpression reduced HUVEC migration, filopodia number/length, and tube formation. Furthermore, HIF-1α expression was positively related to A2M, and the secretion of sFLT-1 and PIGF of placental origin was closely related to PE during pregnancy or A2M overexpression in rats.

**Conclusions:**

Our data showed that gestational A2M overexpression can be considered a contributing factor leading to PE, causing detective uterine spiral artery remodeling and aberrant placental vascularization.

**Supplementary Information:**

The online version contains supplementary material available at 10.1186/s12916-023-02807-9.

## Background

As a multifactorial disorder, PE is a serious blood pressure condition associated with excessive proteinuria or organ dysfunction in pregnant women after the 20th week of pregnancy; its pathological mechanism is poorly understood to date [1, 2]. Notably, PE is the dominant factor causing maternal and fetal morbidity and mortality worldwide [3, 4], and subsequent risks of PE are particularly severe, endangering the lives of both mothers and their babies [4]. Persistent hypoxia in the placenta is a major factor in the pathogenesis of PE. Hypoxia-induced oxidative stress in PE can cause an imbalance between proangiogenic factors (such as vascular endothelial growth factor (VEGF) and placental growth factor [PlGF]) and antiangiogenic factors (such as soluble fms-like tyrosine kinase-1 [sFlt1]), thereby impacting on placental vascular function [5]. In addition, the renin–angiotensin–aldosterone system (RAAS) plays a key role in the high blood pressure of early-onset PE, i.e., there is an increased sensitivity to circulating angiotensin II (ANG II) [6]. Furthermore, compared with normal pregnancies, the serum levels and activities of most components of the RAAS in PE are decreased, especially the levels of AT1R autoantibodies (AT1R-AA), Ang I, Ang II, and Ang-(1-7) [7, 8]. These biologically active molecules enhance vasoconstriction of the resistance arteries in the placenta, leading to increased hypoxia in PE.

Growing evidence indicates that the development of PE may be most likely due to the inadequate remodeling of the uterine spiral artery during the process of vascular remodeling because of dysfunctional trophoblasts [9]. Proper uterine spiral artery remodeling is accomplished when the disruption of the maternal uterine muscular elastic wall facilitates the invasion of extravillous trophoblasts (EVTs), i.e., when epithelial and vascular smooth muscle cells (VSMCs) are replaced by infiltrating EVTs [10]. Transforming growth factor β1 (TGFβ1) derived from uterine natural killer (uNK) cells regulates vascular smooth muscle cell apoptosis and migration, which ensures proper remodeling of the spiral artery [11]. If this process does not proceed correctly, inadequate remodeling of the uterine spiral artery restricts the blood supply to the placenta and subsequently leads to placental hypoxia, which will increase the possibility of PE occurrence [12].

Defective placental angiogenesis is thought to be involved in the pathogenesis of PE [13]. For example, suppressing placental angiogenesis with suramin (a VEGF inhibitor), pregnancy-associated plasma protein-A2 (PAPP-A2), or induction of oxidative stress during pregnancy might lead to maternal hypertension, placental dysfunction, and fetal growth retardation, i.e., the diagnostic index of PE [14–16]. Furthermore, the activate autophagy by protein kinase Cβ (PKCβ) downregulation leads to impaired placental angiogenesis and ultimately induces PE-like symptoms in mice [17]. Taken together, these data suggest the importance of improper placental angiogenesis in the pathogenesis of PE.

A2M is a serum panprotease inhibitor that plays a role in a unique “trapping” mechanism [18–20]. In addition, A2M exerts anti-infective and anti-inflammatory effects through trapping and inhibiting proteases released by neutrophils [21]. A2M is mainly synthesized in the liver and expressed in the brain, heart, and reproductive tract, and in these tissues, A2M is involved in many physiological functions and pathological changes [18, 22, 23]. A variety of important angiogenetic factors are inactivated by binding to A2M, such as basic fibroblast growth factor (bFGF), VEGF, and PlGF [22]. In particular, A2M can coordinately regulate the uterine vasculature during pregnancy [22], which prompted us to investigate the biological functions of A2M in the context of PE. We hypothesize that the increased A2M expression might play a negative role in uterine spiral artery remodeling and placental angiogenesis during pregnancy, thereby contributing to the development of PE.

## Methods

### Human tissue collection

Placental tissues were obtained from 53 healthy pregnancies and 52 pregnant women with early-onset PE (diagnosed before 34 gestational weeks). Serum samples were collected from pregnant women in early pregnancy (22 healthy pregnancies in the normal group and 18 early-onset PE patients in the PE group), middle pregnancy (23 healthy pregnancies and 12 early-onset PE patients), late pregnancy (35 healthy pregnancies and 30 early-onset PE patients), and a week after delivery (18 healthy pregnancies and 21 early-onset PE patients). These pregnant women, including healthy pregnancies and early-onset PE pregnancies, were hospitalized in the Department of Gynecology and Obstetrics of Overseas Hospital, Jinan University, China, from 1 January 2018 to 23 May 2021. The PE diagnosis was based on the criteria issued by the International Society for the Study of Hypertension in Pregnancy (ISSHP) in 2018 [24]. The inclusion and exclusion criteria for tissue collection are listed in Table 1.

**Table 1:** Inclusion and exclusion criteria

**Inclusion criteria**	
• Asian	
• Age: 19–40 years	
• Singleton pregnancy	
• Within the third trimester of pregnancy	
• Meeting the diagnostic criteria for early-onset pre-eclampsia	
• Free of chronic diseases (kidney disease diabetes, hypertension, or other chronic diseases), autoimmune disorders, infections, or hepatitis in preconception	
• Obtain patient informed consent	
**Exclusion criteria**	
• Multiple gestations	
• Fetal congenital malformation	
• Fetal chromosomal disorders	
• History of chronic diseases	
• Complicated with serious internal or surgery-related disease	

The placental villi and decidual tissues were immediately collected after delivery, washed in ice-cold phosphate-buffered saline (PBS) 2 or 3 times to remove the blood, and fixed with liquid nitrogen or 4% paraformaldehyde for further study. The placental villous tissues in the first trimester of pregnancy were obtained from cases of uncomplicated pregnancies and fixed with 4% paraformaldehyde. In addition, peripheral venous blood was collected from pregnant women in the normal group and the early-onset PE group at three sampled times of gestation: 11–13^+6^ weeks of gestation, 14–27^+6^ weeks of gestation, and the first day of the latest hospital admission (usually occurring within a week before delivery). Postnatal blood was collected from the third day after delivery, and umbilical cord blood was collected immediately after delivery. Maternal and umbilical cord blood were collected into EDTA vacuum blood collection tubes and centrifuged (3000×g, 4 °C, 15 min), and the supernatant (i.e., plasma) was extracted and stored at −80 °C for further study.

This study was approved by the Ethics Committee of Overseas Hospital, Jinan University, China (approval number: KY-2021-054) and conducted in accordance with the Declaration of Helsinki. Signed informed consent was obtained from all study participants.

### Animal model

SPF Sprague–Dawley rats (6~8 weeks age, 180~200 g weight) were purchased from Beijing Vital River Laboratory Animal Technology Co., Ltd. (SCXK 2012-0001, Beijing, China). Based on a previous report [25], we established an A2M-overexpression rat model via tail vein injection as previously described [26]. Briefly, on gestational day (GD) 8.5, rats (excluding non-pregnant rats) were injected with adenoviruses expressing A2M (OBiO Technology Co., Shanghai, China), and the sequencing results are shown in Additional file 1: Supplementary Result 1. We injected an adenoviral dose of approximately 1–2×10^9^ pfu per animal, and the adenoviruses were dissolved in phosphate-buffered saline (PBS) to a total volume of 400 μl. The treated rats were sacrificed on GD19.5 (corresponding to the third trimester) for further study. The following parameters were assessed: blood pressure, blood flow, and proteinuria. The primary outcome of this study will be hypertension with blood pressure measurement. Secondary outcomes constitute blood flow, proteinuria, and histological analyses to measure the morphology and cell function of the spiral artery and placental vascular. For the rat samples, the pregnant rats were first euthanized to collect placentas and fetuses. According to Resource Equation Approach [27], a total of 20 rats were studied, the rats were randomly divided into two groups (*n* = 10 in each group): the control and A2M-overexpression groups (note: the rats used in this experiment came from at least three different modelling batches). Random numbers were generated using the standard = RAND() function in Microsoft Excel. Each rat was euthanized by cervical dislocation after the experiment. Experiments involving animals were performed in accordance with the ARRIVE guidelines. All experimental processes involving animal treatments were conducted in accordance with the procedures of the Ethics Committee for Animal Experimentation, Jinan University (approval number: 20210302-46).

### Blinding

For each animal, at least seven different investigators were involved as follows: J.W. was the only person aware of the group allocation based on the randomization table. P.Z. administered intravenous tail vein injections with the assistance of J.W.. Then, G.W. performed the data analysis with the support of X.C.. Finally, P.Z., G.W., R.L., and X.Y. (also unaware of the group allocation) were responsible for the outcome assessment.

### Measuring blood pressure and Doppler ultrasound evaluation as well as proteinuria

As previously described [28, 29], the blood pressure of conscious pregnant rats was measured using an automated computerized tail-cuff system after five consecutive training periods (Visitech BP2000, Visitech Systems, Inc., USA). The blood flow of the rat uterine artery was measured using a high-resolution ultrasound device (Esaote MyLab30 Gold, Esaote, Genova, Italy) to obtain two-dimensional images. Twenty-four-hour urine samples were collected on GD7.5 and GD19.5 for urine protein analysis.

### Histological analysis

Hematoxylin and eosin (HE) and periodic acid Schiff (PAS) staining were performed as follows. The tissues were fixed in 4% paraformaldehyde and subsequently embedded in paraffin. Then, 4-μm-thick cross-sections were processed and stained with HE or PAS for morphological analysis.

Immunohistochemical and immunofluorescent staining were performed as follows. Human or rat tissue was fixed in 4% paraformaldehyde, dehydrated, embedded in paraffin wax, and serially sectioned at a thickness of 4 μm. The sections were incubated with primary antibodies overnight at 4 °C. Subsequently, the sections were stained with fluorescent secondary antibodies. The nuclei were stained with DAPI (Invitrogen). The sections were imaged using a fluorescence microscope (Olympus BX53, Tokyo, Japan). A minimum of 5 random images from 3 samples were analyzed per group. Immunohistochemical statistical analysis was conducted with the Fromowitz comprehensive scoring method [30]. The details of the antibodies are listed in Additional file 1: Supplementary Table 1.

### Western blotting analysis

Proteins from human and rat tissues, HUASMCs, HTR-8/SVneo cells, and HUVECs in Western blotting experiments were analyzed with at least three replicates as previously described [31]. The details of the antibodies are listed in Additional file 1: Supplementary Table 2.

### Enzyme-linked immunosorbent assay (ELISA)

Whole blood samples were collected from human and rat maternal or umbilical cord blood. The substances to be tested in the sera were measured by UV spectrophotometry using detection kits according to the manufacturer’s instructions (Mbbiology Biological, Jiangsu, China). The kit details are provided in Additional file 1: Supplementary Table 3.

### Transmission electron microscopic analysis

Biopsies from rat kidneys (1 mm^3^) were fixed for 2–4 h at 4 °C, washed, and stored overnight at 37°C. The fixed samples were then prepared for ultrathin sectioning. After uranium–lead double staining, the samples were incubated at room temperature overnight, and images were collected and analyzed under a transmission electron microscope (HT7700, Hitachi, Japan).

### Flow cytometry

An Annexin V-FITC apoptosis kit (88-8005-72, Thermo Fisher, USA) was used to determine the apoptosis rates of HUASMC and HTR-8/SVneo cells by flow cytometry analysis. The cells were analyzed using a FACS flow cytometer (Becton-Dickinson, San Jose, CA, USA). The acquired data were analyzed using FCS-Express software version 3.0 (De Novo).

Cell cycle analysis was also performed by flow cytometry. Briefly, the cells were collected and washed in PBS, followed by fixation in ethanol (70%). After overnight incubation at −20 °C, the cells were stained with PI and subjected to flow cytometry. Then, the distribution of cells in the G1, S, and G2/M phases of the cell cycle was determined.

### Wound healing assay

A total of 5 × 10^5^ HTR-8/SVneo cells or HUVECs administered vehicle or A2M-expressing adenovirus vectors over 48 h were seeded in 6-well plates and grown to reach confluent monolayers. Then, a 2-μl pipette tip was used to create the scratches. The images of migrated cells were recorded at 0–36 h. The percentage of wound closure was analyzed.

### Transwell invasion/migration assay

Transfected cells (1×10^5^ HTR-8/SVneo cells or HUVECs in 100 μl serum-free medium) were seeded into transwell inserts (8 -μm pores; #3422, Costar, Cambridge, MA, USA), and the rest of the protocol was previously described [32] (note: the cells were serum-starved for 24 h before being harvested from the plates).

### Tube formation assay

HUVECs (5 × 10^4^) were plated on the Matrigel-coated wells of 24-well plates and incubated for 6 h. HUVEC tubes were evaluated under an inverted fluorescence microscope (Nikon TE300, Japan). The length of the formed tubes was analyzed. The experiments were performed in triplicate.

### Data analysis

In all of our experimental studies, each experiment was performed at least in triplicate, and blinded outcome assessment was implemented. The mean coefficients of variation (CVs) for triplicate values were calculated, and a grand mean CV was then determined based on these values. Statistical analysis was performed using the SPSS 23.0 statistical package program. Construction of statistical charts was performed using the GraphPad Prism 8 software package (GraphPad Software, CA, USA). *T* tests were used to analyze the normally distributed continuous variables, and Mann–Whitney *U* tests were used to analyze the skewed variables (data were normally distributed). All values are presented as the mean ± SD or median (interquartile range). The rates were compared using Fisher’s exact and Pearson’s chi-square tests, which were employed to establish whether there was any difference between the control and experimental data. *P* < 0.05 was considered significant [31, 33, 34]. All animal experiments statistics data and the exact value of *n* are presented in Additional file 1: Supplementary Table 4.

## Results

### A2M was predominantly expressed in the vascular smooth muscle of the spiral artery and feto-placental endothelium, and its expression was significantly elevated in PE patients

**Fig. 1:**
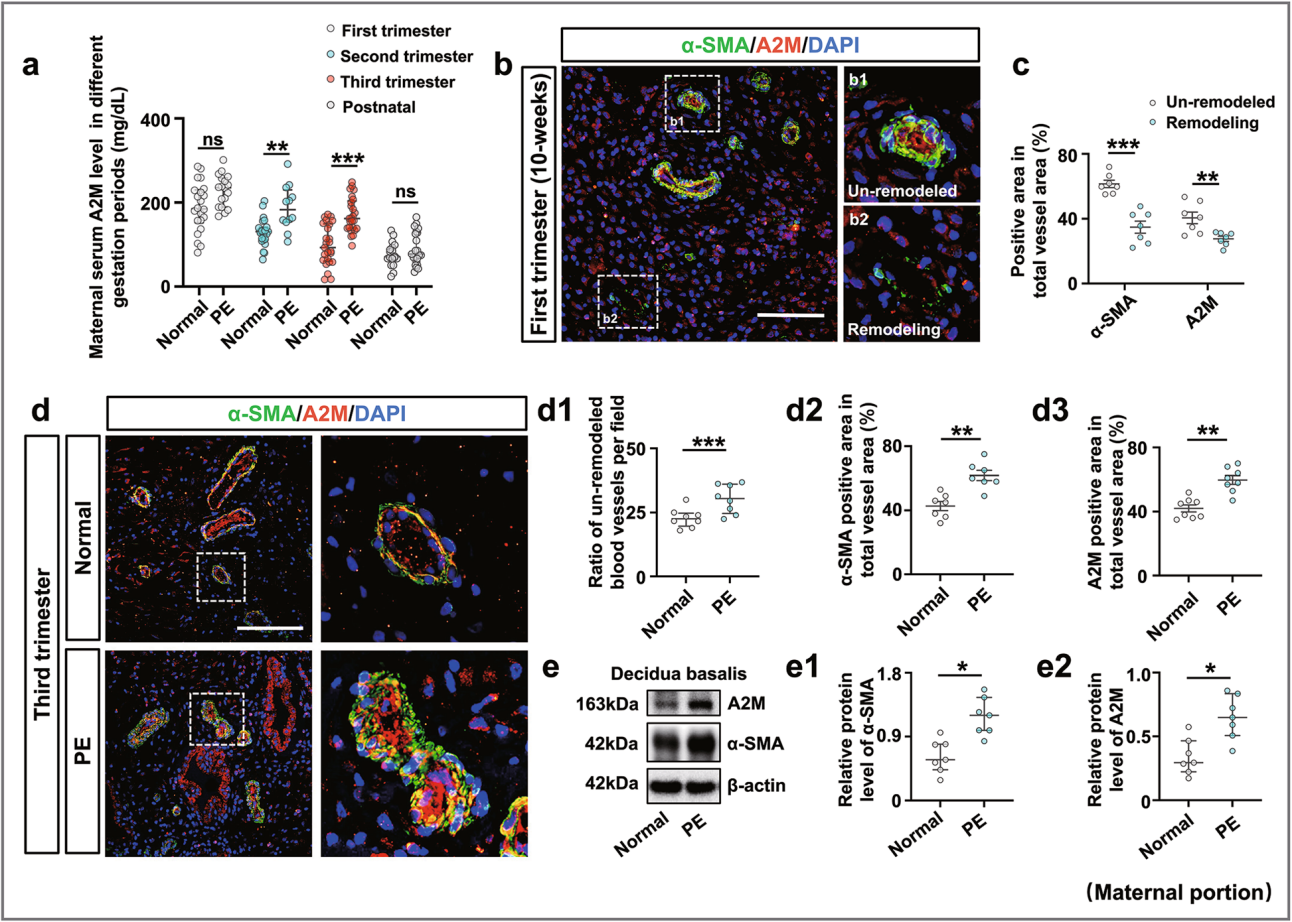

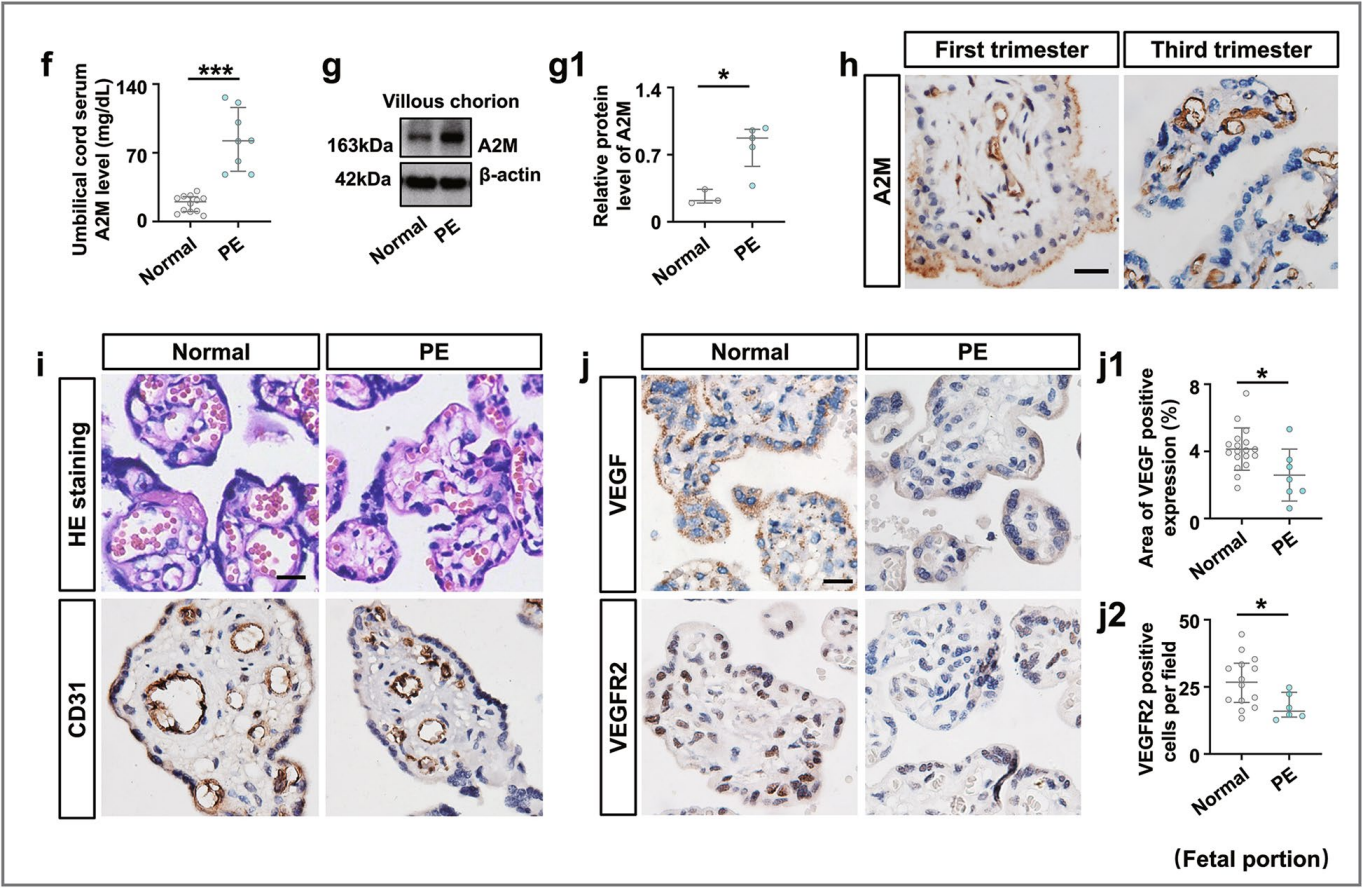
Assessing A2M expression in the maternal portion and fetal portion of the placenta. **a** Determination of the A2M levels in maternal serum obtained from the first, second, and third trimesters of pregnancy and the third day after delivery in the normal and PE groups by ELISA. **b**, **c** Representative immunofluorescence staining of A2M and α-SMA in the cross-sections of the spiral artery from the decidua basalis of the first trimester (counterstained with DAPI) (**b**), and **c** is the quantitative analysis of the positive expression area of α-SMA and A2M in the total vessel. **d**, **d1**–**d3** Representative immunofluorescent staining of α-SMA and A2M in the cross-sections of the spiral artery from the decidua basalis of the third trimester (counterstained with DAPI) (**d**), **d1** is the analysis of the ratio of un-remodeled blood vessels per field, and **d2**–**d3** is the quantitative analysis of the positive expression area of α-SMA (**d2**) and A2M (**d3**) in the total vessel. **e**, **e1**–**e2** Western blotting data showing the expression of α-SMA and A2M in the decidua basalis of the third trimester (**e**), and **e1**–**e2** are the quantitative analysis of the expression of α-SMA (**e1**) and A2M (**e2**) in the normal and PE groups. **f** Determination of the A2M levels in umbilical cord serum in the normal and PE groups by ELISA. **g**, **g1** Western blotting data showing the level of A2M in the villous chorion of the third trimester (**g**), and **g1** is the quantitative analysis of A2M expression in the normal and PE groups. **h** A2M immunohistochemical analysis of cross-sections of first or third trimester villous chorion. **i** Representative HE and CD31 immunohistochemical staining on cross-sections of villous chorion of the third trimester from the normal and PE groups. **j**, **j1**–**j2** VEGF and VEGFR2 immunohistochemical staining on cross-sections of the villous chorion of the third trimester from the normal and PE groups (**j**), and **j1**–**j2** show the quantitative analysis of VEGF (**j1**) and VEGFR2 (**j2**) expression in the two groups. Scale bars = 100 μm in **b** and **d**; 20 μm in **h**–**j**. *P < 0.05, **P < 0.01, ***P < 0.001

In this study, the data of 53 healthy pregnant women and 52 pregnant women with early-onset PE were statistically analyzed to assess their clinical and laboratory characteristics and adverse pregnancy outcomes (Table 2). The results are consistent with the characteristics of PE (Additional file 1: Supplementary Result 2). Using ELISA and immunofluorescent staining, we demonstrated that maternal serum A2M levels in the second and third trimesters were significantly elevated in PE patients compared to healthy subjects, but there was no significant difference in the postnatal period (Fig. 1a). Immunofluorescent staining of A2M and α-SMA showed that A2M was predominantly expressed in the smooth muscles of the spiral artery in the human decidua basalis (Fig. 1b, d), and there was significantly higher expression of α-SMA and A2M in the un-remodeled spiral arteries than in the remodeled spiral arteries (Fig. 1c). In addition, we discovered the more un-remodeled spiral arteries in PE decidua basalis than in normal pregnancy (Fig. 1d, d1). Furthermore, immunofluorescence double staining of the cross-sections of the spiral arteries demonstrated significantly higher expression of α-SMA and A2M in the PE group compared to the normal group (Fig. 1d, d2–d3), which was confirmed by Western blotting data from the human decidua basalis (Fig. 1e, e1–e2).

**Table 2:** Clinical and laboratory characteristics of the study population

Variables	Normal ***n***=53	PE ***n***=52	***P***-value
Gestational age at diagnosis in weeks	**—**	30.3±0.5	**—**
Maternal age (years)	30.3±0.5	30.9±0.7	0.562
Weight (kg)	66.2±1.2	69.5±1.8	0.142
Height (cm)	160.8±0.8	155.8±3.2	0.129
BMI (kg/m^2^)	25.6±0.4	27.6±0.4	0.019**
Systolic blood pressure (mmHg)	116.6±1.4	163.5±2.1	<0.001***
Diastolic blood pressure (mmHg)	73.5±1.1	102.0±1.4	<0.001***
MAP (mmHg)	87.8±1.0	123.1±1.5	<0.001***
Gestational weeks at delivery (week)	39.3±0.1	37.0±0.4	<0.001***
Proteinuria (g/24 h)	**—**	1.0±0.1	**—**
Platelet count (×10^9/L)	210.0±6.9	193.1±7.7	0.105
Birthweight (kg)	3.2±0.5	2.4±0.1	<0.001***
Umbilical cord length (cm)	50.6±1.0	45.88±1.3	0.004**
Amniotic fluid volume (ml)	547.7±39.9	467.9±20.5	0.079
Placental weight (g)	555.2±8.7	466.9±14.0	<0.001***
Cesarean section rate (*n*, %)	16 (30.2)	42 (80.8)	<0.001***
Maternal adverse outcomes (*n*, %)	1 (1.9)	15 (35.7)	<0.001***
Placental abruption (*n*, %)	0 (0.0)	3 (7.1)	0.076
PPH (*n*, %)	1 (1.9)	2 (4.8)	0.547
Eclampsia (*n*, %)	0 (0.0)	0 (0.0)	**—**
Incomplete uterine rupture (*n*, %)	0 (0.0)	2 (4.8)	0.149
Cardiac insufficiency (*n*, %)	0 (0.0)	1 (1.9)	0.310
Fetal/neonatal adverse outcomes (*n*, %)	4 (7.5)	30 (57.7)	<0.001***
Preterm birth (<37 weeks) (*n*, %)	0 (0.0)	16 (30.8)	<0.001***
LBW (*n*, %)	0 (0.0)	15 (28.8)	<0.001***
Myocardial damage (*n*, %)	1 (1.9)	13 (25.0)	<0.001***
PFO (*n*, %)	1 (1.9)	8 (15.4)	0.014*
Hypoalbuminemia (*n*, %)	0 (0.0)	16 (30.8)	<0.001***
Neonatal anemia (*n*, %)	0 (0.0)	9 (17.3)	0.002**
Neonatal hyperbilirubinemia (*n*, %)	3 (5.7)	15 (28.0)	0.002**

Differences between the groups were compared with the Mann–Whitney *U* test or chi-square test or Fisher’s exact test; *P<0.05, **P<0.01, ***P<0.001. *PE*, pre-eclampsia; *weight*, the maternal weight in the first day of the latest hospital admission (usually occur within a week before delivery); *BMI*, body mass index; *MAP*, mean arterial pressure; *PPH*, postpartum hemorrhage; *LBW*, low-birth-weight infant; *PFO*, patent foramen ovale. Values are mean ± S.E.M

Using ELISA and Western blotting, we demonstrated that the A2M levels in umbilical cord serum and villous chorion were significantly elevated in PE patients compared to healthy subjects (Fig. 1f, g, g1), and A2M was specifically expressed in the vascular endothelium of the villous chorion in human placenta, as revealed by immunohistochemistry staining of A2M (Fig. 1h). In addition, fewer blood vessels and smaller vascular capacitances were observed in PE patient placentas than in normal group placentas (Fig. 1i), while we discovered the significantly decreased expression of VEGF and its receptor VEGFR2 in the PE group (Fig. 1j, j1–j2).

### The A2M-overexpression rat model closely mimicked the phenotypes observed in PE patients

**Fig. 2:**
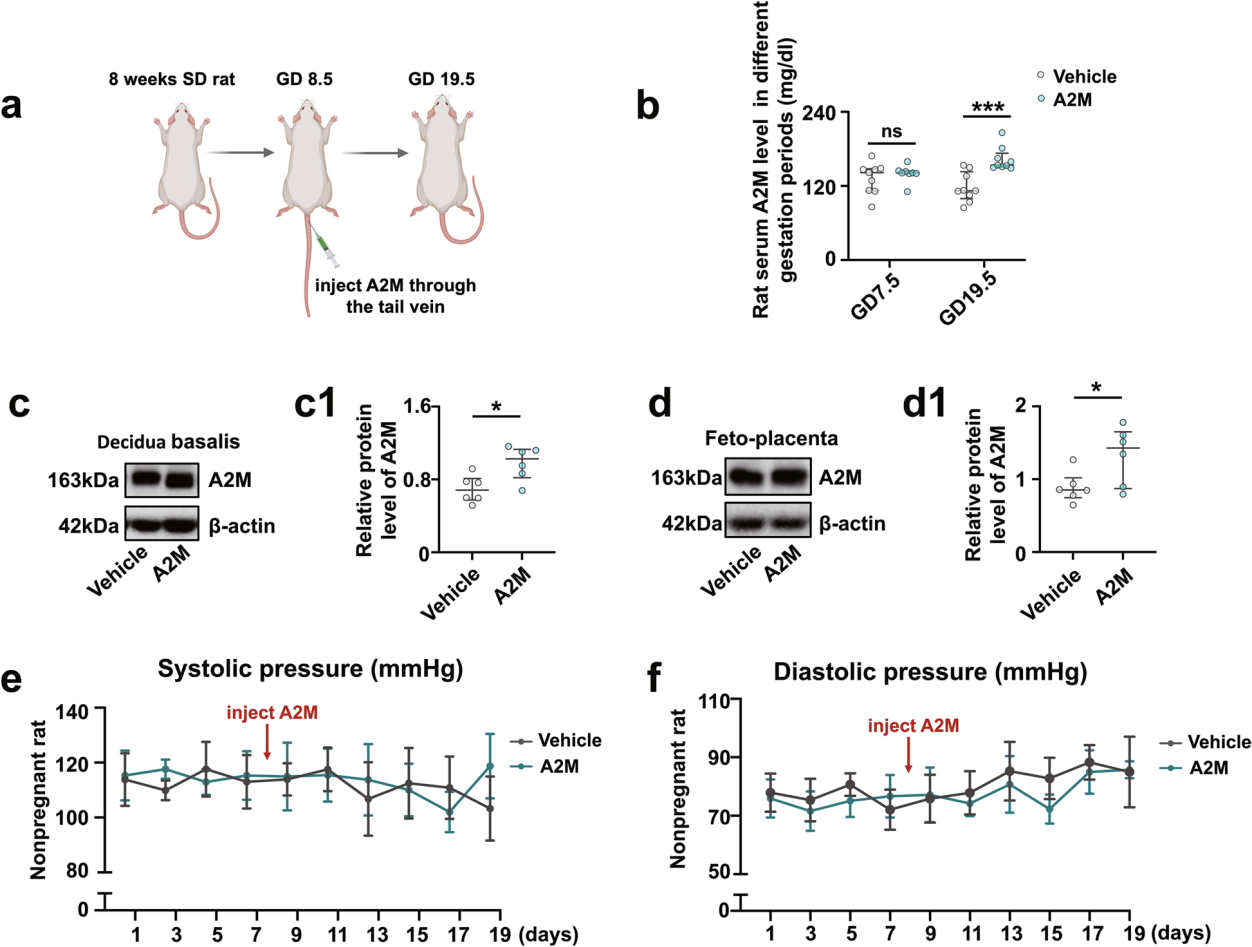

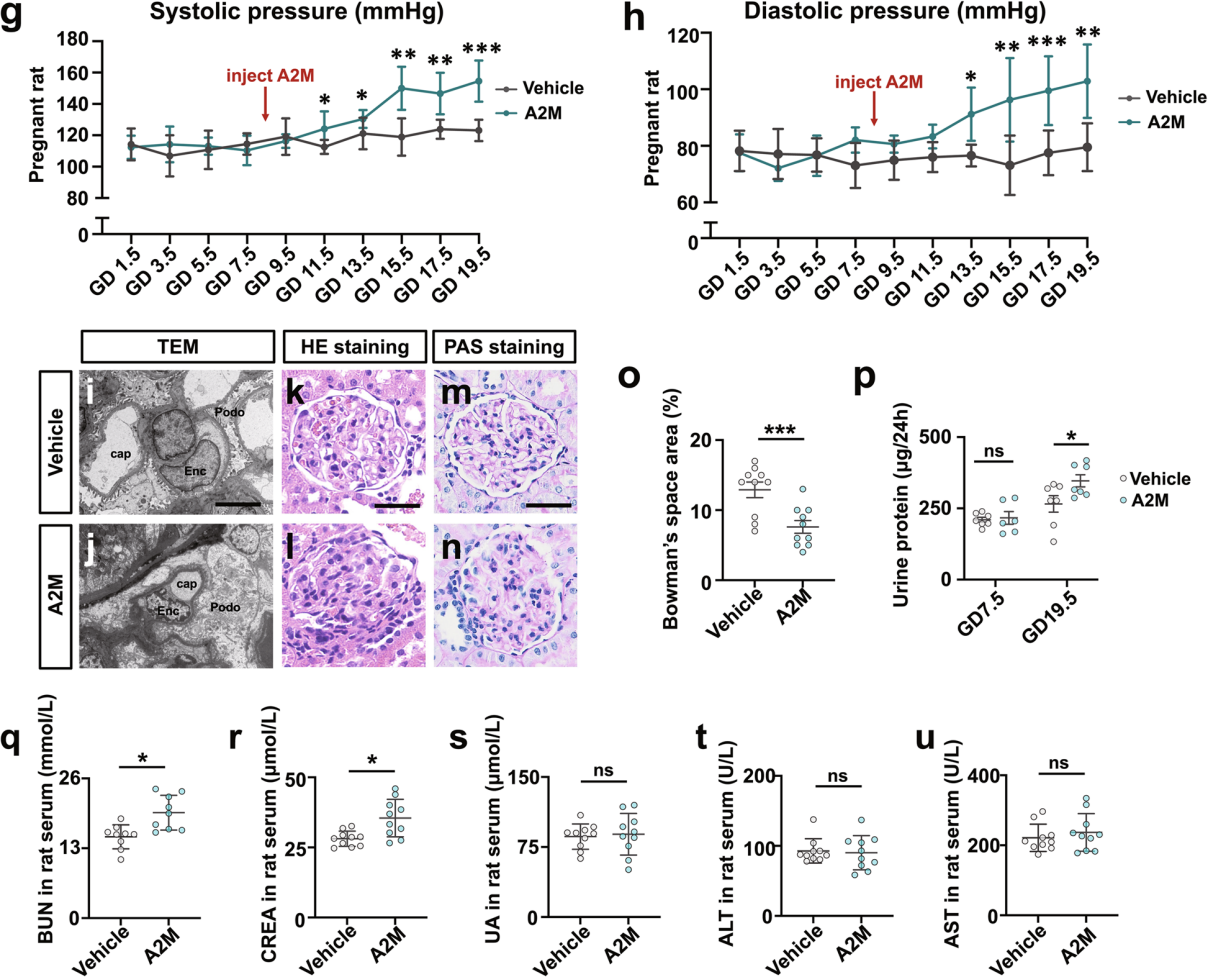
Assessment of blood pressure and liver and kidney function indices in the A2M-overexpression rat model. **a** Schematic illustration of the establishment of a humanized A2M-overexpression pregnant rat model. **b** Determination of the A2M levels in the sera of pregnant rats during different stages of gestation (GD7.5 and GD19.5) in the control and A2M-overexpression groups by ELISA. **c**, **d**, **c1**–**dI** Western blotting data showing the level of A2M in the decidua basalis (**c**) and feto-placenta (**d**), and **c1**–**d1** are the quantitative analysis of A2M expression in control and A2M-overexpression rats. **e**–**h** The trends in the variation in systolic (**e**, **g**) or diastolic (**f**, **h**) pressure from the control and A2M-overexpressing groups in both non-pregnant (**e**, **f**) and pregnant (**g**, **h**) rats. **i**, **j** Representative TEM images of the kidneys from both groups. **k**–**o** Representative HE staining (**k**–**l**) and PAS staining (**m**, **n**) of cross-sections of rat kidneys from the control and A2M-overexpression groups, and **o** shows the quantitative analysis of the area of Bowman’s space from both groups. **p** Urine protein (mg/24 h) levels at GD7.5 and GD19.5 from the control and A2M-overexpression rats. **q**–**u** Determination of the levels of BUN (**q**), CREA (**r**), UA (**s**), ALT (**t**), and AST (**u**) in rat sera from the control and A2M-overexpression groups. Scale bars = 5 μm in **i**, **j**; 50 μm in **k**–**n**. Abbreviations: TEM, transmission electron microscope; Enc, endothelial cell; Podo, podocyte; cap, capillary; BUN, blood urea nitrogen; CREA, creatinine; UA, uric acid; ALT, alanine aminotransferase; AST, aspartate aminotransferase. *P < 0.05, **P < 0.01, ***P < 0.001

The successful establishment of the A2M-overexpression rat model (Fig. 2a) was verified by the data from ELISA (Fig. 2b) and Western blotting (Fig. 2c, d). There were significantly increased A2M levels in the maternal serum and A2M-overexpression rats at gestational day 19.5 (Fig. 2b) and enhanced expression of A2M protein in the A2M-overexpression rat decidua basalis (Fig. 2c, c1) and feto-placenta (Fig. 2d, d1) compared to those in the control (vehicle) rats.

To evaluate whether manifestations of the A2M-overexpression rat model were consistent with the clinical manifestations of PE, we first dynamically measured the blood pressure of rats administered either vehicle or A2M-expressing adenovirus vectors. The results showed no changes in blood pressure in non-pregnant rats after A2M administration (Fig. 2e, f), but both the systolic and diastolic pressures were significantly but gradually enhanced in A2M-administered rats within 1 week of A2M administration (Fig. 2g, h). TEM images of the rat glomerulus clearly indicated that A2M overexpression caused ultrastructural damage to the glomerulus, such as edema, collapsed vascular lumen, and endothelial hyperplasia (Fig. 2i, j). HE and PAS staining showed that A2M overexpression indeed caused an increase in inflammatory cell infiltration and even morphological damage in the glomerulus, such as a narrower Bowman’s capsule, compared to the control (Fig. 2k–o). The 24-h urine protein measure was performed using a BCA protein assay, and the results showed a significant increase in the A2M-overexpression rat compared to the control rats at gestational day 19.5 (Fig. 2p). The ELISA results indicated significant increases in the BUN and CREA level but no significant changes in the ALT, AST and UA levels in the A2M-administered rat serum compared to control rat serum (Fig. 2q–u). In addition, maternal A2M overexpression led to fetal growth restriction (e.g., fewer and smaller fetuses, and placenta size) (Additional file 1: Fig. S1).

### A2M overexpression led to elevated vascular resistance and defective uterine spiral artery remodeling

**Fig. 3:**
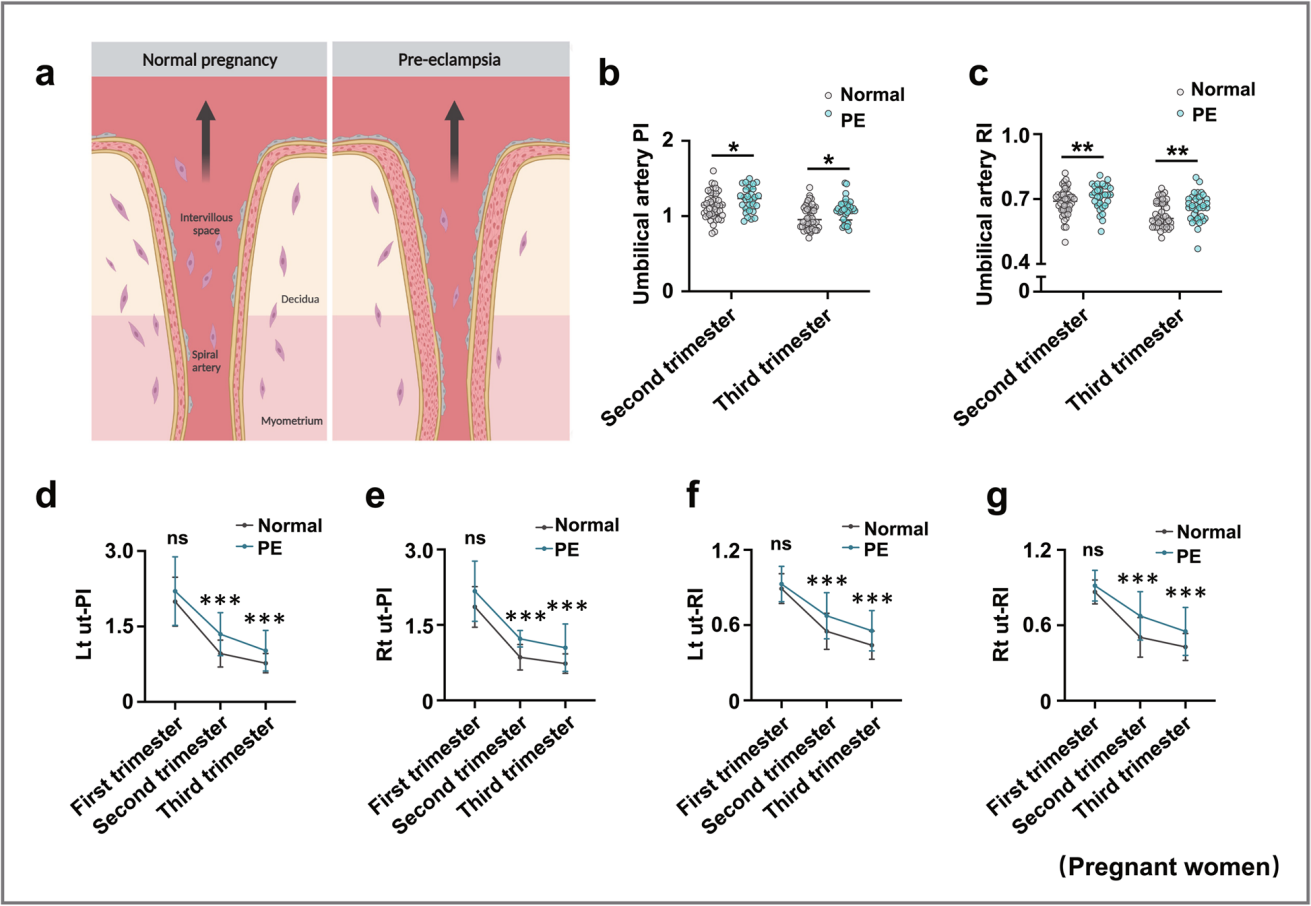

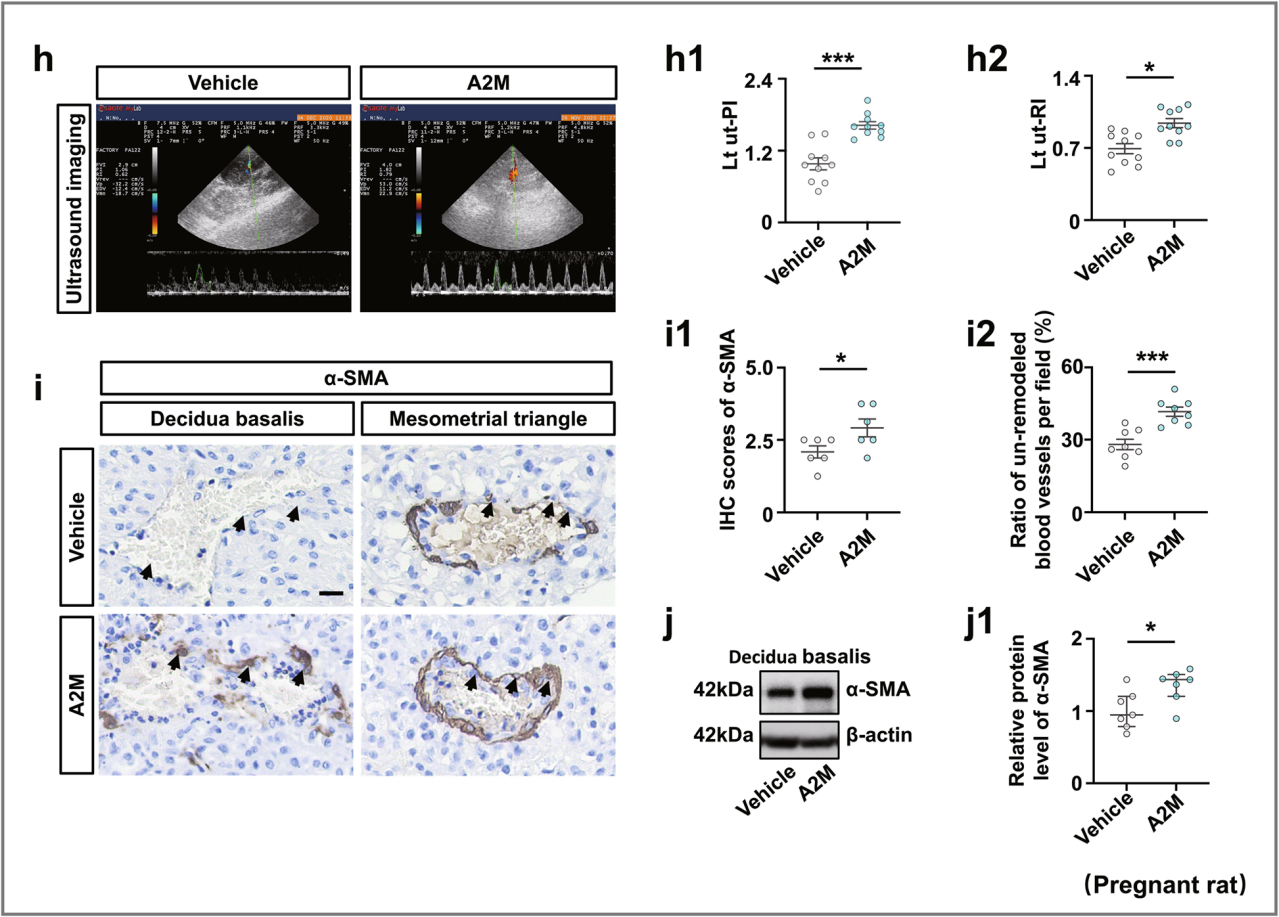
Assessing the index of spiral artery remodeling in pregnant women and A2M-overexpression rats. **a** Schematic illustration of PE establishment due to failure of spiral artery remodeling. **b**, **c** Determination of the values of umbilical artery PI (**b**) and RI (**c**) in the normal and PE groups during the second and third trimesters of pregnancy. **d**–**g** Determination of the levels of Lt ut-PI (**d**), Rt ut-PI (**e**), Lt ut-RI (**f**), and Rt ut-RI (**g**) in the normal and PE groups during the first, secondary, and third trimesters of pregnancy. **h**, **h1**–**h2** Representative ultrasonography of rat uterine spiral artery from control and A2M-overexpression rats (**h**), and **h1**–**h2** show the quantitative analysis of Lt ut-PI (**h1**) and Lt ut-RI (**h2**) from the above groups. **i**, **i1**–**i2** α-SMA immunohistochemical analysis of cross-sections of spiral arteries from the control and A2M-overexpression groups (**i**). **i1** is the quantitative analysis of α-SMA expression, and **i2** is the ratio of un-remodeled spiral arteries per field in the two groups. **j**, **j1** Western blotting data showing the level of α-SMA in the decidua basalis (**j**) and **j1** is the quantitative analysis of α-SMA expression in the control and A2M-overexpressing groups. Scale bars = 100 μm in **i**. Abbreviations: PI, pulsatility index; RI, resistive index; Lt ut, left uterine artery; Rt ut, right uterine artery. *P < 0.05, **P < 0.01, ***P < 0.001

Because A2M is highly expressed in the vascular smooth muscles of pregnant women with early-onset PE, we reasonably hypothesized that PE was caused by notably defective uterine spiral artery remodeling [35], which further led to increased placental vascular resistance [36] (Fig. 3a). Therefore, we assessed a series of vascular resistance indexes [37] in human pregnant women (Fig. 3b–g). The results showed that umbilical cord PI (Fig. 3b) and RI (Fig. 3c) were significantly increased in the second and third trimesters of pregnancy in women with PE compared to healthy women, and there were significant increases in the left uterine artery pulsatility index (Lt ut-PI) (Fig. 3d), the right uterine artery pulsatility index (Rt ut-PI) (Fig. 3e), the left uterine artery resistive index (Lt ut-RI) (Fig. 3f), and the right uterine artery resistive index (Rt ut-RI) (Fig. 3g) in the pregnant women with PE compared to the healthy pregnant women. Moreover, ultrasound evaluation of pregnant rats (Fig. 3h) indicated that A2M overexpression significantly enhanced Lt ut-PI (Fig. 3h1) and Lt ut-RI (Fig. 3h2) compared to the control. Immunohistochemistry displayed enhanced expression of α-SMA in the spiral artery and more un-remodeled spiral arteries in A2M-overexpression rats (Fig. 3i, i1–i2). Similar results were obtained for α-SMA expression by Western blotting (Fig. 3j).

### A2M overexpression-induced defective uterine spiral artery remodeling was partially derived from aberrant cell proliferation and apoptosis

**Fig. 4:**
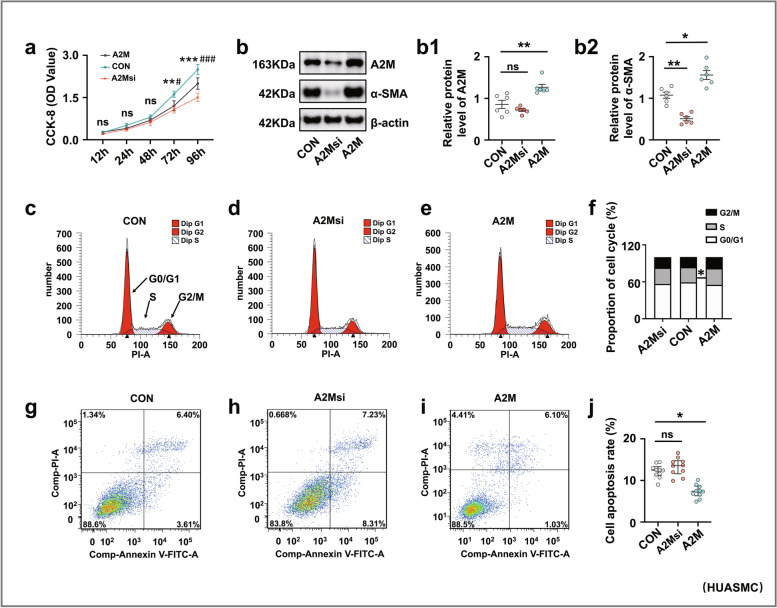

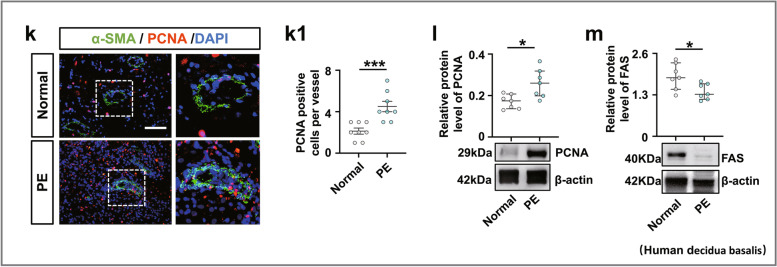
Assessment of proliferation and apoptosis in human umbilical artery smooth muscle cells (HUASMCs) following manipulation of A2M expression and human decidua basalis. **a**, **b**, **b1**–**b2** Determination of HUASMC viability in the control, A2M-overexpression and A2M-downregulated groups after 12-, 24-, 48-, 72-, and 96-h incubation by CCK-8 assay (**a**). Western blotting data showing the expression of A2M and α-SMA in HUASMCs (**b**), and **b1**–**b2** show the quantitative analysis of A2M (**b1**) and α-SMA (**b2**) expression among the control, A2M-overexpression, and A2M-downregulated groups. **c**–**f** Flow cytometry data showing the analysis of the DNA contents in HUASMCs transfected with negative control (control) (**c**), A2M-silencing vector (A2Msi) (**d**), or A2M-overexpressing vector (A2M) (**e**), and **f** shows the quantitative analysis of the proportion of cells in each phase of the cell cycle in the three groups. **g**–**j** Apoptosis of HUASMCs transfected with negative control (control) (**g**), A2M-silencing vectors (**h**), or A2M-overexpression vector (**i**) was determined by flow cytometry using the annexin V-FITC/PI apoptosis assay, and **j** shows the quantitative analysis of the cell apoptosis rates in the three groups. **k**, **k1** Representative double immunofluorescence staining of α-SMA and PCNA on the cross-sections of the spiral arteries (counterstained with DAPI) of the third trimester from the normal and PE groups, and **k1** shows the quantitative analysis of PCNA expression in both groups. **l**, **m** Western blotting data showing the expression of PCNA (**l**) and FAS (**m**) and the quantitative analysis of the human decidua basalis of the third trimester from the normal and PE groups. Scale bars = 50 μm in **k**. *P < 0.05, **P < 0.01, ***P < 0.001

We investigated the effect of A2M expression on the proliferation and apoptosis of uterine spiral artery smooth muscle cells by manipulating A2M expression in HUASMCs. The CCK8 assay (Fig. 4a) and Western blotting (Fig. 4b, b1–b2) indicated that cell proliferation was dramatically increased in the A2M overexpression group and decreased in the A2M-downregulated group. Flow cytometry analysis demonstrated that knockdown of A2M expression in HUASMCs did not affect cell cycle progression or apoptosis, but A2M overexpression caused cell cycle arrest at the S phase (Fig. 4c–f) and suppression of cell apoptosis (Fig. 4g–j). Experiments on human samples reinforced the observation as follows. Double immunofluorescence staining of α-SMA and PCNA showed increased numbers of PCNA-positive cells in the decidua basalis of human patients with PE (Fig. 4k, k1), and similar results were obtained for PCNA expression by Western blotting (Fig. 4l). In addition, Western blotting analysis showed that FAS expression was decreased in the decidua basalis of PE patients (Fig. 4m).

To determine the effects of EVTs on uterine spiral artery remodeling [38, 39] in the context of A2M overexpression, wound healing and transwell invasion assays were used, and the results demonstrated that A2M overexpression significantly suppressed the migratory and invasive abilities of HTR-8/SVneo cells (Additional file 1: Fig. S2). We also found that trophoblast cell proliferation was decreased and cell apoptosis was enhanced in the context of A2M overexpression (Additional file 1: Fig. S3).

### TGFβ1 played an important role in the overproliferation of vascular smooth muscle cells in the context of A2M overexpression

**Fig. 5:**
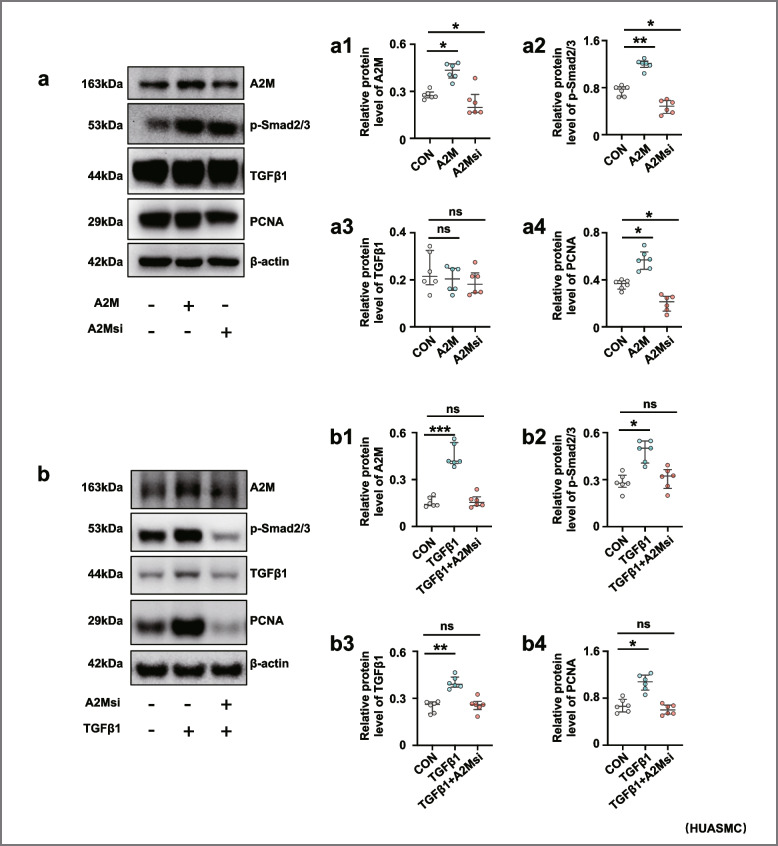

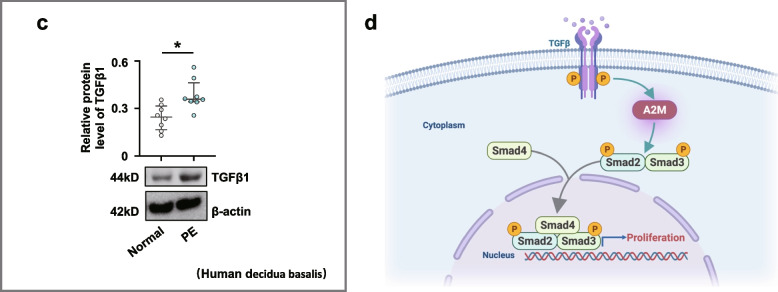
Assessment of proliferation and TGFβ signaling in smooth muscle cells following the manipulation of A2M expression and human decidua basalis. **a**, **a1**–**a4** Western blotting data showing the expression of A2M, p-Smad2/3, TGFβ1, and PCNA in HUASMCs transfected with negative control, A2M-overexpresion, or A2M-silencing vectors (**a**), and **a1**–**a4** shows the quantitative analysis of A2M (**a1**), p-Smad2/3 (**a2**), TGFβ1 (**a3**), and PCNA (**a4**) in the control, A2M-overexpression and A2M-downregulated groups. **b**, **b1**–**b4** Western blotting data showing the expression of A2M, p-Smad2/3, TGFβ1, and PCNA in HUASMCs transfected with negative control, treat with TGFβ1 and A2M-silenced + treated with TGFβ1 (**b**), and **b1**–**b4** show the quantitative analysis of A2M (**b1**), p-Smad2/3 (**b2**), TGFβ1 (**b3**), and PCNA (**b4**) expressions among the control, treatment with TGFβ1 and A2M-silenced + treated with TGFβ1 groups. **c** Western blotting data showing the expression of TGFβ1 and the quantitative analysis of TGFβ1 expression in human decidua basalis of the third trimester from the normal and PE groups. **d** Schematic illustration of A2M overexpression leading to cell proliferation through the TGFβ signaling pathway. *P < 0.05, **P < 0.01, ***P < 0.001

Western blotting showed that A2M overexpression enhanced the expression of PCNA and p-Smad2/3, while A2M downregulation suppressed the expression of both genes in HUASMCs (Fig. 5a, a1–a4); furthermore, the addition of TGFβ1 significantly increased A2M, PCNA, and p-Smad2/3 expression (Fig. 5b, b1–b4). Western blotting data demonstrated that TGFβ1 expression was elevated in the decidua basalis of patients with PE in comparison to normal pregnant women (Fig. 5c).

### A2M overexpression during pregnancy dramatically restricted feto-placental angiogenesis

**Fig. 6:**
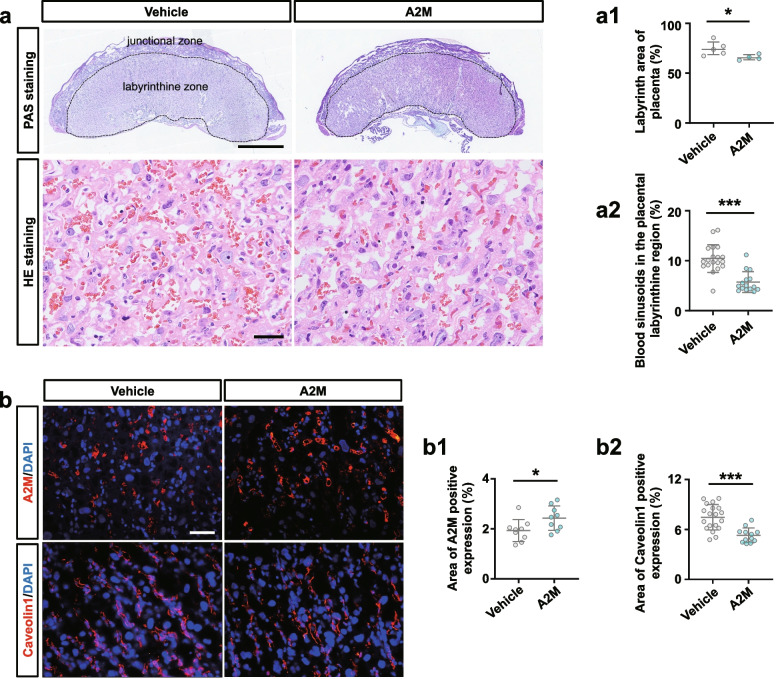

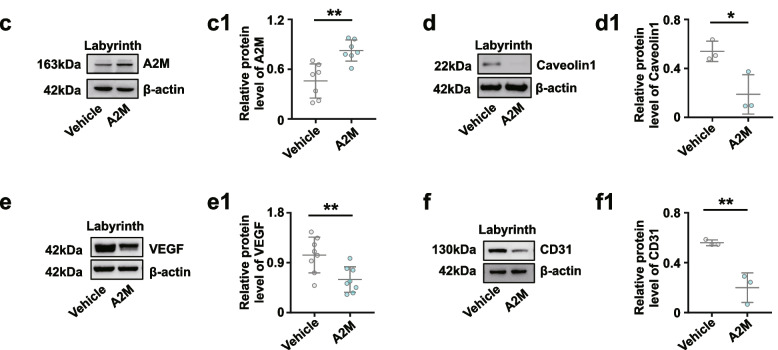
Assessment of feto-placental angiogenesis in the A2M-overexpression rat model. **a**, **a1**–**a2** Representative PAS and HE staining (**a**) on the cross-sections of rat placenta from the control and A2M-overexpression groups, and **a1**–**a2** are the quantitative analysis of the area of the placental labyrinthine zone (**a1**) and the area of placental blood sinusoid (**a2**) from the control and A2M-overexpression groups. **b**, **b1**–**b2** Representative immunofluorescent staining of A2M and Caveolin1 in the cross-sections of rat placenta from the control and A2M-overexpression groups (**b**), and **b1**–**b2** are the quantitative analysis of positive areas of A2M and Caveolin1 expression (%). **c**–**f**, **c1**–**f1** Western blotting data showing the expression of A2M (**c**), Caveolin1 (**d**), VEGF (**e**), and CD31 (**f**) in the placental labyrinthine zone from the control and A2M-overexpression groups. **c1**–**f1** shows the quantitative analysis of A2M (**c1**), Caveolin1 (**d1**), VEGF (**e1**), and CD31 (**f1**) expression from the control and A2M-overexpression groups. Scale bars = 2 mm in the upper panel of **a**; 100 μm in the lower panel of **a**; 50 μm in **b**. *P < 0.05, **P < 0.01, ***P < 0.001

PAS staining showed that the ratios of labyrinth size to whole placenta size were smaller in A2M-overexpression rats than in control rats (Fig. 6a, a1). HE staining showed that the area of blood sinusoids was decreased in the labyrinth layer of A2M-overexpression rats compared to the control (Fig. 6a, a2). Immunofluorescence staining revealed the elevated A2M and reduced Caveolin1 expression in the labyrinth of the A2M-overexpression rats compared to control rats (Fig. 6b, b1–b2). These findings were confirmed by Western blotting data from the rat labyrinth (Fig. 6c, d, c1–d1). In addition, Western blotting showed that VEGF and CD31 expression in A2M-overexpression placental labyrinth was significantly higher than that in the control (Fig. 6e, f, e1–f1).

**Fig. 7:**
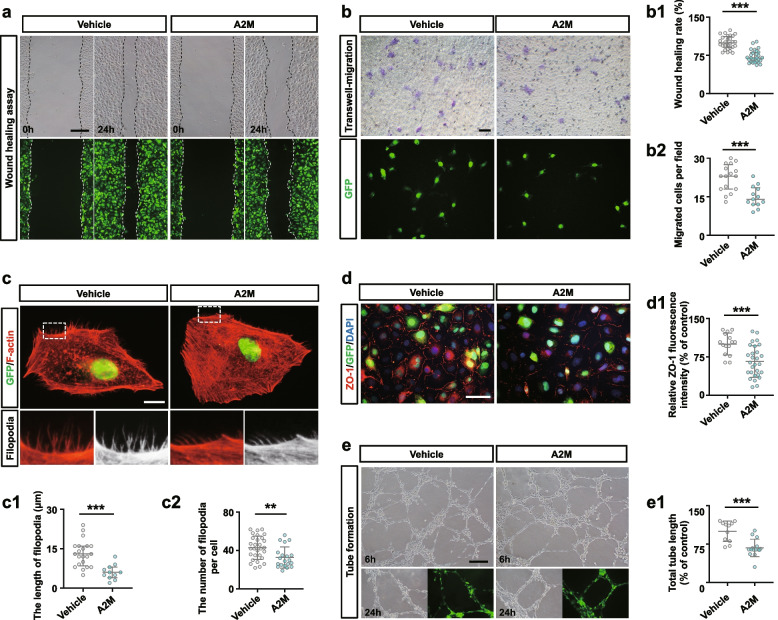
Assessing cell migration and tube formation of HUVECs following manipulation of A2M expression. **a**, **a1** Representative images of wound healing assays of HUVECs at 24 h from negative control (control) or A2M-overexpression groups (**a**), and **a1** shows the quantitative analysis of relative cell migration in both groups. **b**, **b1** Representative images of transwell migration assays of HUVECs transfected with either negative control or A2M-overexpression vectors after 24 h of incubation (**b**), and **b1** shows the quantitative analysis of the numbers of migrated cells in both groups. **c**, **c1**–**c2** Representative fluorescent staining and high magnification images of F-actin on the negative control or A2M-overexpression HUVECs (**c**), and **c1**–**c2** show the quantitative analysis of the numbers (**c1**) and length (**c2**) of filopodia of each cell in both groups. **d**, **d1** Representative immunofluorescent staining of ZO-1 on the negative control or A2M-overexpression HUVECs (**d**), and **d1** shows the quantitative analysis of relative ZO-1 fluorescence intensity (% of control) in both groups. **e**, **e1** Representative images of tube formation assays of HUVECs after 6- and 24-h incubation from negative control or A2M-overexpression groups, and **e1** shows the quantitative analysis of total tube length (% of control) at 6-h incubation in both groups. Scale bars = 200 μm in **a** and **e**; 50 μm in **b** and **d**; 20 μm in **c**. **P < 0.01, ***P < 0.001

Next, wound healing and transwell migration assays were used to assess the migration and invasion capabilities of HUVECs. The results showed that both the wound-closure rate (Fig. 7a, a1) and the number of HUVECs that migrated to the lower side of the membrane (Fig. 7b, b1) significantly decreased in A2M overexpression groups compared to control groups. Filopodia affect cell migration, as presented in Fig. 7c, F-actin staining in HUVECs indicated the reduced length and numbers of filopodia in A2M overexpression HUVECs compared to control (Fig. 1c, c1–c2). Furthermore, A2M overexpression in HUVECs significantly decreased ZO-1 expression (Fig. 7d, d1), and significantly suppressed HUVEC tube formation (Fig. 7e, e1), implying the inhibition of epithelial cell migration capability.

### Placental ischemia/hypoxia derived from defective spiral artery remodeling and aberrant placental angiogenesis promoted sFLT-1 and suppressed PIGF secretion

**Fig. 8:**
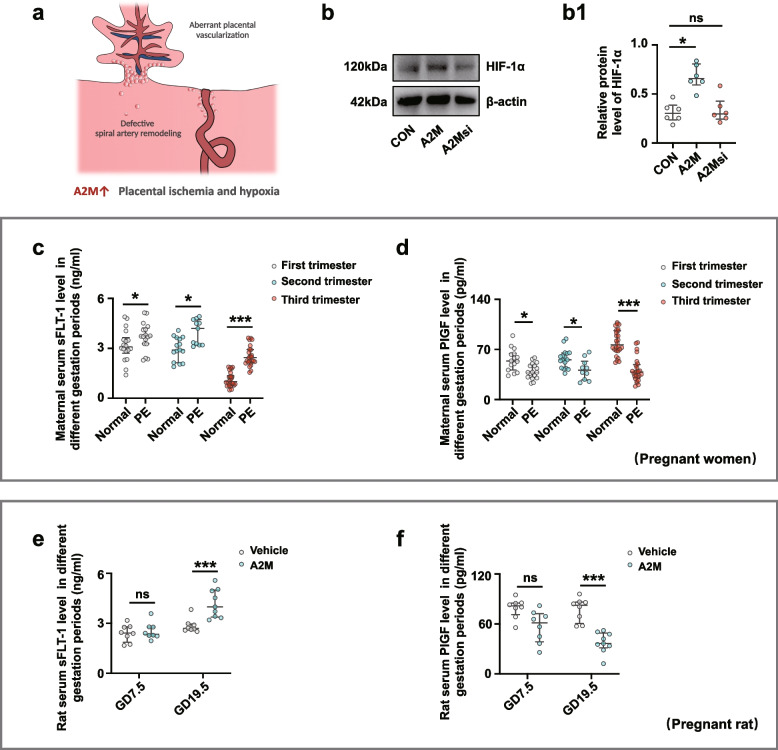
Measurement of HIF-1α and sFIt-1/PIGF levels in maternal serum and A2M-manipulated HUASMCs. **a** Schematic illustration of inappropriate spiral artery remodeling and abnormal placental angiogenesis in the presence of A2M overexpression. **b**, **b1** Western blotting data showing the expression of HIF-1α in the control, A2M-overexpression, and A2M-downregulated groups (**b**), and **b1** shows the quantitative analysis of HIF-1α expression in the three groups. **c**, **d** ELISA data showing the sFLT-1 (**c**) and PIGF (**d**) levels in human maternal serum obtained from the first, second, and third trimesters of pregnancy in the normal and PE groups. **e**, **f** ELISA data showing the sFLT-1 (**e**) and PIGF (**f**) levels in maternal rat serum at GD7.5 and GD19.5 in the control and A2M-overexpression groups. *P < 0.05, ***P < 0.001

Western blotting demonstrated high HIF-1α expression in A2M-overexpression HUASMCs but no change in HIF-1α expression when A2M was downregulated (Fig. 8b–b1). These results suggested that defective spiral artery remodeling as well as aberrant placental angiogenesis causes ischemia, which further induces the high expression of HIF-1α. Notably, we found that the sFLT-1 level was significantly higher and the PIGF level was significantly reduced in the PE group compared to that in the normal group during the different stages of pregnancy (Fig. 8c, d). There was a negative correlation between PIGF and A2M (*R* = −0.768, *P* <0.001) and a positive correlation between sFLT-1 and A2M (*R* = 0.659, *P* <0.001) in maternal plasma of the pre-eclampsia women (Additional file 1: Fig. S4). In the A2M-overexpression rat model, sFLT-1 levels increased (Fig. 8e), and PIGF levels decreased (Fig. 8f), significantly in the sera of A2M-overexpression rats compared to the control rats at 19.5 days of gestation.

## Discussion

**Fig. 9:**
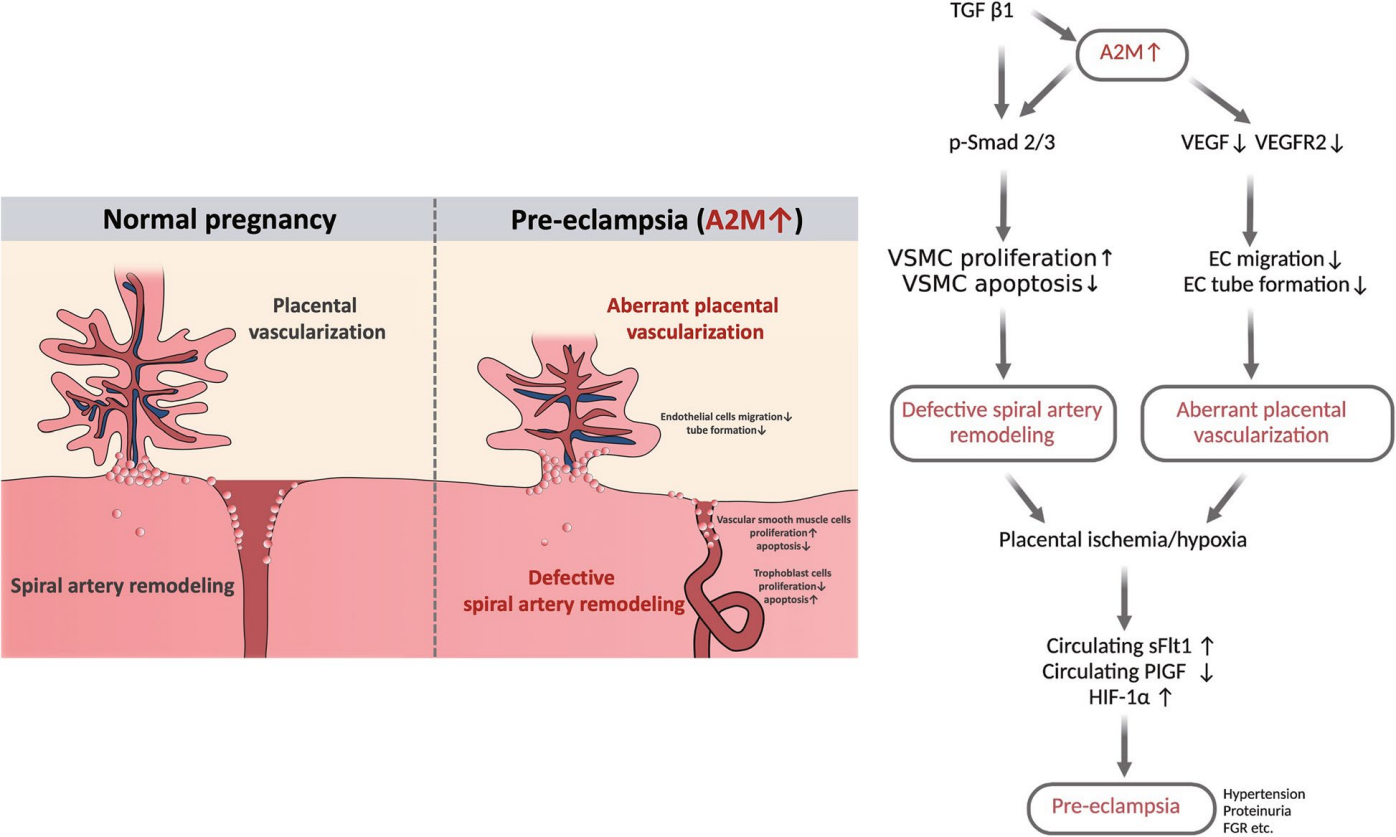
A proposed model that shows the underlying assumption for the role of A2M in the development of PE. The proposed mechanism by which A2M is involved in the occurrence of PE via its negative impact on uterine spiral artery remodeling and placental angiogenesis

A previous study demonstrated that mild systemic inflammation actually occurred in healthy pregnant women, and the status worsened in PE [40]. In the present study, we reported that an increased level of A2M was involved in the occurrence of early-onset pre-eclampsia via its negative impact on uterine spiral artery remodeling and placental angiogenesis (Fig. 9). Because A2M can reduce endogenous/exogenous inflammatory injury, it has been used in a variety of managements of orthopedic pains, such as subacromial bursitis, lateral epicondylitis, and Achilles tendonitis [41]. A2M is also used to ascertain inflammation status in degenerative, immune, digestive, and urinary system diseases [42–45]. In this study, we found an imbalance between pro-inflammatory and anti-inflammatory cytokines in pregnancy with PE (Additional file 1: Fig. S5), implying the possibility that A2M is involved in developing PE during pregnancy. As a unique proteinase inhibitor, A2M does not completely remove pro-inflammatory cytokines in PE, while elevated A2M can be responsible for the PE-like phenotype, suggesting that A2M may play a double-edged sword role in the development of PE [46].

Both uterine spiral arterial modification and placental angiogenesis are crucial events that provide sufficient blood supply to fully perfuse the placenta and thus provide the demands of the growing fetus during pregnancy [47, 48]. Therefore, if the modifications are interrupted for any reason, the consequence would be the inadequate modification of spiral arteries and aberrant placental angiogenesis, which could greatly increase the risk of the PE scenario [49]. Hence, in this study, we investigated the possible effects of A2M overexpression on both events in developing PE during pregnancy.

Our clinical pregnancy cohort study showed that A2M was predominantly expressed in the spiral artery from the third trimester of pregnancy, which is similar to the expression of α-SMA; the A2M levels in the sera of pregnant women with early-onset PE were significantly increased in the secondary and third trimesters of pregnancy, and similar results were observed in the human uterine decidua basalis (Fig. 1a–e), which indicated the occurrence of inadequate uterine spiral artery remodeling. In other words, A2M overexpression might be closely associated with the pathophysiology of PE by negatively affecting uterine spiral artery remodeling. We speculated that A2M inhibited related proteases, cytokines, and growth factors [50], which would impact the survival and physiological functions of smooth muscle cells during spiral artery remodeling. In addition, we found that the overexpression of A2M in the placental vascular bed dramatically restricted placental angiogenesis (Fig. 1f–j); thus, abnormal vascularization of placental villi is obviously a key factor that cannot be neglected. It should be noted that A2M was reported to be involved in atherosclerosis by facilitating the lipogenesis of vascular smooth muscle cells [51], suggesting a possible pathological mechanism in PE, i.e., a similar vascular lesion in pregnancy.

Overexpression of A2M was established in non-pregnant and pregnant rats (Fig. 2) to investigate the possibility of A2M involvement in PE. The different blood pressure responses to A2M overexpression between non-pregnant and pregnant rats notably indicated that the increased blood pressure induced by A2M overexpression was associated with pregnancy (Fig. 2e–h). Furthermore, the following phenotypes were observed: morphological changes in rat kidneys and proteinuria in the rats that undoubtedly contributed to the onset of PE in A2M-overexpression rats (Fig. 2i–u) and intrauterine growth restriction and poor placentation (Additional file 1: Fig. S1) that were extremely coincident with the diagnosis indexes of PE [52, 53]. Additionally, A2M overexpression also greatly suppressed placental vascularization (Fig. 6). All of these data prompted us to further explore the causal relationship between A2M overexpression and PE.

Regarding uterine spiral artery remodeling [54], we found that A2M overexpression promoted HUASMC cell proliferation and inhibited HUASMC cell apoptosis (Fig. 4a–j), implying that the normal replacement of uterine spiral endothelial cells and smooth muscle cells was restricted in the context of A2M overexpression. In other words, A2M overexpression might prevent the cascade regulation from the normally progressive breakdown of the endothelial and smooth muscle cells in uterine spiral arteries, thereby increasing the risk of developing PE. On the other hand, the features of trophoblast cells overexpressed by the A2M gene were assessed because the migration and invasion of trophoblast cells play very important roles in uterine spiral artery remodeling [55, 56]. The experimental results revealed that both migratory and invasive abilities, as well as apoptosis and proliferation of trophoblast cells, were dramatically affected by elevated A2M expression (Additional file 1: Fig. S2-S3), suggesting that EVTs of fetal origin could be influenced by overloaded A2M through an unclear mechanism. To further address the abovementioned phenotypes, we assessed TGFβ signaling following A2M gene manipulation since the TGFβ superfamily is known to regulate vascular endothelial and smooth muscle cell responses during vessel remodeling under both physiological and pathological conditions [57]. As expected, our experimental evidence (Fig. 5a, b) clearly showed a causal relationship between A2M gene expression and TGFβ signaling activation, i.e., TGFβ signaling might be responsible for the A2M overexpression-induced cell responses of endothelial and smooth muscles in the uterine spiral artery described above, which then results in an inappropriate vascular remodeling. This finding is well established and confirmed by the fact that TGFβ1 was highly expressed in the uterine spiral artery smooth muscles of pregnant women with early-onset PE (Fig. 5c).

On the other hand, A2M overexpression affected placental vascularization, such as the coarctation of the placental labyrinth and the reduced expression of specific endothelial markers (Caveolin1, CD31) and VEGF (Fig. 6). Angiogenesis is involved in cell proliferation, migration, adhesion, and tube formation [58]. In this study, A2M overexpression clearly inhibited HUVEC migration, probably by inhibiting the formation of endothelial filopodia and cell–cell junctions, as well as tube formation (Fig. 7a–e). These results revealed that A2M overexpression brought about the obstacle in placental vascularization, which would provoke or aggravate the occurrence of PE.

The initiating event in early-onset PE is usually considered to be placental ischemia and hypoxia, which in turn cause the release of various factors of placental origin and eventually affect maternal blood pressure during pregnancy [59–61]. A2M-induced excessive proliferation of smooth muscle cells aggravated inappropriate vascular remodeling, as well as aberrant placental vascularization, which may be crucial factors for placental ischemia and hypoxia. Notably, ischemia and anoxia were proven to be closely associated with the level of A2M gene expression (Fig. 8b). Similarly, we observed a significant increase in sFLT-1 levels and a decrease in PIGF levels in PE patient serum in the second and/or third trimesters of pregnancy (Fig. 8c, d), and sFLT-1 and PIGF levels were closely associated with the level of A2M in maternal plasma of the pre-eclampsia women (Additional file 1: Fig. S4). Furthermore, a similar trend of change in the levels of sFLT-1 and PIGF in the sera of pregnant rats was clearly observed in A2M-overexpression rats (Fig. 8e, f). These results suggest that A2M overexpression participates in inducing the release of these factors of placental origin in the context of exacerbated placental ischemia/hypoxia.

The renin–angiotensin–aldosterone system (RAAS) plays a vitally important role in maintaining adequate uteroplacental circulation in normal pregnancy, as well as the development of PE [62, 63]. In addition, angiotensin II type 1 receptor agonistic autoantibody (AT1-AAs) was first discovered in women with PE and can activate the angiotensin II type 1 receptor in response to placental ischemia, thereby increasing vasoconstriction of the placental vasculature [64]. In this study, many components of the RAAS and AT1-AAs showed abnormal expressions in PE (Additional file 1: Fig. S6-S8). Therefore, dysregulation of the RAAS might act as a subsequent mechanism of PE in the context of A2M overexpression.

## Conclusions

In summary, our data clearly showed how A2M was involved in the pathophysiology of PE. Briefly, A2M is predominantly expressed in the vascular smooth muscle of the spiral artery and feto-placental vasculature, and its level is elevated under the influence of activated TGFβ1 in the context of PE; the high expression in turn causes the excessive proliferation and reduced apoptosis of vascular smooth muscle cells in the uterine spiral artery as well as insufficient trophoblast migration and invasion (i.e., inappropriate uterine spiral artery remodeling). Meanwhile, the overexpressed A2M in placental villi also greatly restricts placental angiogenesis. The two comprehensive results exacerbate placental ischemia/hypoxia and alter the release of sFLT-1 and PIGF from the placenta, which lead to the occurrence of maternal hypertension, proteinuria, and fetal growth restriction, i.e., PE. In addition, this work has several limitations, such as the lack of precise molecular mechanisms underlying A2M involvement in PE progression, small numbers of PE and control samples, and the lack of prospective studies. Finally, it is important to design more integrated experiments to completely address the pathophysiological mechanism underlying PE. If so, we can expect that A2M will become a potential biomarker and therapeutic target for PE in the future.

## Supplementary Information

**Additional file 1: Table S1.** Antibodies for immunohistochemistry. **Table S2.** Antibodies for Western blotting. **Table S3.** Details of Elisa kits. **Table S4.** Animal experiments statistics data. **Supplementary Result 1.** A2M sequencing result. **Supplementary Result 2.** The clinical and laboratory characteristics and adverse pregnancy outcomes of pregnant women enrolled in this study. **Figure S1.** Assessment of placental and fetal development in the A2M-overexpression rat model. **Figure S2.** Determining HTR-8/SVneo cell migration and cell viability following A2M upregulation. **Figure S3.** Determining HTR-8/SVneo cell proliferation and apoptosis following A2M upregulation. **Figure S4.** Correlation between PlGF, sFLT-1 and A2M levels in maternal plasma of the preeclampsia women. **Figure S5.** Determining the serum and placental levels of human inflammatory cytokins and NF-κB. **Figure S6.** Determining key components of the RAAS system in human serum. **Figure S7.** Determining key components of the RAAS system in rat serum in the presence of high levels of A2M. **Figure S8.** Schematic illustration of the changes in key components of the RAAS system in the presence of high A2M levels.

**Additional file 2.** Western blot source data.

## Data Availability

Data sharing is not applicable to this article as no datasets were generated or analyzed during the current study.

## Abbreviations

A2M: Alpha-2-macroglobulin
ANG II: Angiotensin II
AT1-AAs: Angiotensin II type 1 receptor agonistic autoantibody
AT1R-AA: AT1R autoantibodies
bFGF: Basic fibroblast growth factor
ELISA: Enzyme-linked immunosorbent assay
EVTs: Extravillous cytotrophoblasts
HE: Hematoxylin and eosin
HUASMC: Human umbilical artery smooth muscle cells
HUVECs: Human umbilical vein endothelial cells
Lt ut-PI: Left uterine artery pulsatility index
Lt ut-RI: Left uterine artery resistive index
PAS: Periodic acid Schiff
PBS: Phosphate-buffered saline
PE: Pre-eclampsia
PKCβ: Protein kinase Cβ
PlGF: Placenta growth factor
RAAS: Renin–angiotensin–aldosterone system
Rt ut-RI: Right uterine artery resistive index
sFlt1: Soluble fms-like tyrosine kinase-1
TGFβ1: Transforming growth factor β1
uNK: Uterine natural killer
VEGF: Vascular endothelial growth factor
VSMCs: Vascular smooth muscle cells
