# Supplementary material for: Alpha-2-macroglobulin is involved in the occurrence of early-onset pre-eclampsia via its negative impact on uterine spiral artery remodeling and placental angiogenesis
Source: BMC Med. 2023 Mar 9;21:90. doi: 10.1186/s12916-023-02807-9 (PMC9999529; doi:10.1186/s12916-023-02807-9)
Supplement: Supplementary file 1 — Additional file 1: Table S1. Antibodies for immunohistochemistry. Table S2. Antibodies for Western blotting. Table S3. Details of Elisa kits. Table S4. Animal experiments statistics data. Supplementary Result 1. A2M sequencing result. Supplementary Result 2. The clinical and laboratory characteristics and adverse pregnancy outcomes of pregnant women enrolled in this study. Figure S1. Assessment of placental and fetal development in the A2M-overexpression rat model. Figure S2. Determining HTR-8/SVneo cell migration and cell viability following A2M upregulation. Figure S3. Determining HTR-8/SVneo cell proliferation and apoptosis following A2M upregulation. Figure S4. Correlation between PlGF, sFLT-1 and A2M levels in maternal plasma of the preeclampsia women. Figure S5. Determining the serum and placental levels of human inflammatory cytokins and NF-κB. Figure S6. Determining key components of the RAAS system in human serum. Figure S7. Determining key components of the RAAS system in rat serum in the presence of high levels of A2M. Figure S8. Schematic illustration of the changes in key components of the RAAS system in the presence of high A2M levels. [file 12916_2023_2807_MOESM1_ESM.docx]

**Contents**

[Supplementary Tables 2](#_Toc10802)

[Supplementary Table 1. Antibodies for immunohistochemistry 2](#_Toc28467)

[Supplementary Table 2. Antibodies for Western blotting 3](#_Toc11535)

[Supplementary Table 3. Details of Elisa kits 4](#_Toc8972)

[Supplementary Table 4. Animal experiments statistical data 5](#_Toc28629)

[Supplementary Results 8](#_Toc1263)

[Supplementary Result 1. A2M sequencing result 8](#_Toc31792)

[Supplementary Result 2. The clinical and laboratory characteristics and adverse pregnancy outcomes of pregnant women enrolled in this study 17](#_Toc14320)

[Supplementary Figures 18](#_Toc4251)

[Supplementary Fig. 1 18](#_Toc20870)

[Supplementary Fig. 2 20](#_Toc8688)

[Supplementary Fig. 3 21](#_Toc23394)

[Supplementary Fig. 4 22](#_Toc17008)

[Supplementary Fig. 5 23](#_Toc22724)

[Supplementary Fig. 6 25](#_Toc25148)

[Supplementary Fig. 7 26](#_Toc5034)

[Supplementary Fig. 8 27](#_Toc15437)

# *Supplementary Tables*

## *Supplementary Table 1. Antibodies for immunohistochemistry*

| **Antibody** | **Original Concentration** | **Dilution** | **Applied Concentration** | **Catalog #** | **Company** | **Country** |
| --- | --- | --- | --- | --- | --- | --- |
| A2M | 0.5 mg/ml | 1:200 | 2.5 μg/ml | ab58703 | Abcam | USA |
| α-SMA | 1 mg/ml | 1:400 | 2.5 μg/ml | ab7817 | Abcam | USA |
| PCNA | 1 mg/ml | 1:400 | 2.5 μg/ml | ab29 | Abcam | USA |
| ZO-1 | 0.667 mg/ml | 1:400 | 1.668 μg/ml | ab221547 | Abcam | USA |
| VEGF | 0.296 mg/ml | 1:300 | 0.987 μg/ml | ab52917 | Abcam | USA |
| VEGFR2 | 0.503 mg/ml | 1:300 | 1.677 μg/ml | ab233693 | Abcam | USA |
| CD31 | 7 µg/ml | 1:300 | 0.023 μg/ml | 77699 | CST | USA |
| TGFβ1 | 0.521 mg/ml | 1:200 | 2.605 μg/ml | ab215715 | Abcam | USA |

## *Supplementary Table 2. Antibodies for Western blotting*

| **Antibody** | **Original Concentration** | **Dilution** | **Applied Concentration** | **Catalog #** | **Company** | **Country** |
| --- | --- | --- | --- | --- | --- | --- |
| A2M | 0.5 mg/ml | 1:1000 | 0.5 μg/ml | ab58703 | Abcam | USA |
| α-SMA | 1 mg/ml | 1:1000 | 1 μg/ml | ab7817 | Abcam | USA |
| PCNA | 1 mg/ml | 1:1000 | 1 μg/ml | ab29 | Abcam | USA |
| TGFβ1 | 0.521 mg/ml | 1:500 | 1.042 μg/ml | ab215715 | Abcam | USA |
| pSmad2/3 | 1 mg/ml | 1:1000 | 1 μg/ml | PA5-36028 | Thermo | USA |
| HIF1α | 0.165 mg/ml | 1:700 | 0.236 μg/ml | ab179483 | Abcam | USA |
| VEGF | 1 mg/ml | 1:1000 | 1 μg/ml | ab46154 | Abcam | USA |
| Caveolin1 | 0.114 mg/ml | 1:1000 | 0.114 μg/ml | ab32577 | Abcam | USA |
| p65 | 0.52 mg/ml | 1:500 | 1.04 μg/ml | ab32536 | Abcam | USA |
| IKBα | 0.091 mg/ml | 1:1000 | 0.091 μg/ml | ab32518 | Abcam | USA |
| CD31 | 7 µg/ml | 1:600 | 0.012 μg/ml | 77699 | CST | USA |
| IL-6 | 101 µg/ml | 1:1000 | 0.101 μg/ml | 12912 | CST | USA |
| TNF-α | 102 µg/ml | 1:500 | 0.204 μg/ml | 11948 | CST | USA |
| β-actin | 1 mg/ml | 1:3000 | 0.333 μg/ml | ab8226 | Abcam | USA |
| GAPDH | 2 mg/ml | 1:3000 | 0.667 μg/ml | ab8245 | Abcam | USA |

## *Supplementary Table 3. Details of Elisa kits*

| **Drug Name** | **Art.No.** | **Company** | **Country** |
| --- | --- | --- | --- |
| A2M (Human) | MB-1240A | Mbbiology Biological | China |
| PIGF (Human) | MB-0099B | Mbbiology Biological | China |
| sFlt-1 (Human) | MB-3845B | Mbbiology Biological | China |
| Angiotensinogen (Human) | MB-0754A | Mbbiology Biological | China |
| AngII (Human) | MB-0004A | Mbbiology Biological | China |
| AT1R (Human) | MB-4358B | Mbbiology Biological | China |
| Ang-(1-7) (Human) | MB-3568A | Mbbiology Biological | China |
| AT1AA (Human) | MB-16056A | Mbbiology Biological | China |
| IL-10 (Human) | MB-3705A | Mbbiology Biological | China |
| IL-6 (Human) | MB-0049A | Mbbiology Biological | China |
| NF-κB (Human) | MB-3525B | Mbbiology Biological | China |
| TNF-α (Human) | MB-0122B | Mbbiology Biological | China |
| A2M (Rat) | MB-7300B | Mbbiology Biological | China |
| PIGF (Rat) | MB-1659B | Mbbiology Biological | China |
| sFlt-1 (Rat) | MB-6627B | Mbbiology Biological | China |
| Angiotensinogen (Rat) | MB-1924B | Mbbiology Biological | China |
| AngII (Rat) | MB-1753B | Mbbiology Biological | China |
| AT1R (Rat) | MB-7498A | Mbbiology Biological | China |
| Ang-(1-7) (Rat) | MB-3391B | Mbbiology Biological | China |
| AT1AA (Rat) | MB-7491B | Mbbiology Biological | China |

## *Supplementary Table 4.* ***Animal experiments statistical data***

| **Fig. 2** | **Statistical index** | | | | **Vehicle** | | | **A2M** | | | ***p* value** | | |  |  |
| --- | --- | --- | --- | --- | --- | --- | --- | --- | --- | --- | --- | --- | --- | --- | --- |
| **b** | Rat serum A2M level | GD 7.5 | | | 132.32±8.40 n=9 | | | 140.07±4.76 n=8 | | | 0.44 | | |  |  |
|  |  | GD 19.5 | | | 117.7±7.95 n=9 | | | 163.14±6.37 n=9 | | | ＜0.001 | | |  |  |
| **c1** | Relative protein level of A2M | | | | 0.87±0.08 n=6 | | | 1.22±0.09 n=6 | | | 0.02 | | |  |  |
| **d1** | Relative protein level of A2M | | | | 0.89±0.09 n=6 | | | 1.32±0.16 n=6 | | | 0.04 | | |  |  |
| **e** | Nonpregnant rat Systolic pressure (mmHg) | | Day 1 | | 113.79±4.82 n=6 | | | 115.29±4.53 n=6 | | | 0.83 | | |  |  |
|  |  |  | Day 3 | | 109.92±1.80 n=7 | | | 112.58±3.51 n=6 | | | 0.52 | | |  |  |
|  |  |  | Day 5 | | 117.58±4.99 n=5 | | | 112.96±2.36 n=6 | | | 0.45 | | |  |  |
|  |  |  | Day 7 | | 113.04±4.88 n=6 | | | 115.29±4.42 n=7 | | | 0.74 | | |  |  |
|  |  |  | Day 9 | | 113.92±2.94 n=5 | | | 114.96±6.16 n=6 | | | 0.88 | | |  |  |
|  |  |  | Day 11 | | 117.54±3.99 n=6 | | | 115.46±4.83 n=6 | | | 0.75 | | |  |  |
|  |  |  | Day 13 | | 106.75±6.69 n=6 | | | 113.75±6.49 n=7 | | | 0.48 | | |  |  |
|  |  |  | Day 15 | | 112.50±6.44 n=6 | | | 110.11±4.81 n=6 | | | 0.77 | | |  |  |
|  |  |  | Day 17 | | 110.88±4.03 n=8 | | | 102.00±3.67 n=6 | | | 0.19 | | |  |  |
|  |  |  | Day 19 | | 103.25±4.13 n=8 | | | 118.75±5.89 n=6 | | | 0.05 | | |  |  |
| **f** | Nonpregnant rat Diastolic pressure (mmHg) | | Day 1 | | 57.01±2.67 n=6 | | | 55.92±3.26 n=6 | | | 0.65 | | |  |  |
|  |  |  | Day 3 | | 55.33±2.99 n=7 | | | 51.66±3.36 n=6 | | | 0.44 | | |  |  |
|  |  |  | Day 5 | | 60.63±1.59 n=5 | | | 55.06±2.77 n=6 | | | 0.1 | | |  |  |
|  |  |  | Day 7 | | 52.05±3.43 n=6 | | | 56.67±3.65 n=7 | | | 0.39 | | |  |  |
|  |  |  | Day 9 | | 55.88±4.10 n=5 | | | 57.13±4.67 n=6 | | | 0.85 | | |  |  |
|  |  |  | Day 11 | | 57.85±3.73 n=6 | | | 54.19±2.17 n=6 | | | 0.43 | | |  |  |
|  |  |  | Day 13 | | 65.25±5.02 n=6 | | | 60.75±4.85 n=7 | | | 0.54 | | |  |  |
|  |  |  | Day 15 | | 62.75±3.54 n=6 | | | 52.25±4.99 n=6 | | | 0.52 | | |  |  |
|  |  |  | Day 17 | | 68.25±2.95 n=8 | | | 65.00±3.72 n=6 | | | 0.52 | | |  |  |
|  |  |  | Day 19 | | 65.00±6.06 n=8 | | | 65.75±1.44 n=6 | | | 0.91 | | |  |  |
| **g** | Pregnant rat Systolic pressure (mmHg) | | GD 1.5 | | | 114.21±3.82 n=7 | | | 112.36±2.63 n=8 | | | 0.69 | | |  |
|  |  |  | GD 3.5 | | | 106.98±4.97 n=7 | | | 114.26±4.04 n=8 | | | 0.27 | | |  |
|  |  |  | GD 5.5 | | | 110.75±4.32 n=8 | | | 113.12±2.08 n=7 | | | 0.65 | | |  |
|  |  |  | GD 7.5 | | | 114.52±2.58 n=7 | | | 110.39±3.56 n=7 | | | 0.37 | | |  |
|  |  |  | GD 9.5 | | | 119.14±4.29 n=7 | | | 116.37±1.64 n=7 | | | 0.57 | | |  |
|  |  |  | GD 11.5 | | | 112.67±2.02 n=5 | | | 124.16±4.20 n=7 | | | 0.04 | | |  |
|  |  |  | GD 13.5 | | | 121.17±4.09 n=6 | | | 131.91±1.36 n=7 | | | 0.02 | | |  |
|  |  |  | GD 15.5 | | | 118.96±4.49 n=7 | | | 147.82±5.18 n=9 | | | 0.001 | | |  |
|  |  |  | GD 17.5 | | | 125.03±2.16 n=8 | | | 142.24±4.06 n=9 | | | 0.003 | | |  |
|  |  |  | GD 19.5 | | | 123.1±2.61 n=7 | | | 154.62±4.38 n=9 | | | ＜0.001 | | |  |
|  | Pregnant rat Diastolic pressure (mmHg) | | GD 1.5 | | | 58.20±3.20 n=7 | | | 57.55±2.48 n=8 | | | 0.87 | | |  |
| **h** |  |  | GD 3.5 | | | 57.15±3.97 n=7 | | | 52.13±1.72 n=7 | | | 0.23 | | |  |
|  |  |  | GD 5.5 | | | 56.75±2.65 n=8 | | | 56.48±2.72 n=7 | | | 0.95 | | |  |
|  |  |  | GD 7.5 | | | 53.06±3.58 n=7 | | | 59.23±4.79 n=7 | | | 0.12 | | |  |
|  |  |  | GD 9.5 | | | 54.94±3.11 n=7 | | | 60.64±1.14 n=7 | | | 0.08 | | |  |
|  |  |  | GD 11.5 | | | 56.02±2.35 n=5 | | | 61.89±2.93 n=7 | | | 0.07 | | |  |
|  |  |  | GD 13.5 | | | 56.61±1.94 n=6 | | | 71.19±3.56 n=7 | | | 0.03 | | |  |
|  |  |  | GD 15.5 | | | 53.13±3.98 n=7 | | | 76.29±4.92 n=9 | | | 0.004 | | |  |
|  |  | | GD 17.5 | | | 57.53±7.90 n=8 | | | 79.47±4.97 n=9 | | | ＜0.001 | | |  |
|  |  |  | GD 19.5 | | | 59.56±3.21 n=7 | | | 82.85±4.32 n=9 | | | 0.001 | | |  |
| **o** | Bowman's space area (%) | | | | 13.30±0.94 n=10 | | | 7.20±0.71 n=10 | | | ＜0.001 | | |  |  |
| **p** | Urine protein (μg/24h) | | | GD 7.5 | | | 210.09±8.52 n=7 | | | 216.52±22.37 n=6 | | | 0.78 | | |
|  |  |  |  | GD 19.5 | | | 265.6±28.83 n=7 | | | 346.60±21.25 n=7 | | | 0.04 | | |
| **q** | BUN in rat serum (mmol/L) | | | | 15.13±0.75 n=9 | | | 19.63±1.08 n=9 | | | 0.003 | | |  |  |
| **r** | CREA in rat serum (μmol/L) | | | | 28.1±0.86 n=10 | | | 35.46±2.22 n=10 | | | 0.005 | | |  |  |
| **s** | UA in rat serum (μmol/L) | | | | 85.37±4.70 n=9 | | | 92.90±6.32 n=9 | | | 0.35 | | |  |  |
| **t** | ALT in rat serum (U/L) | | | | 92.76±5.50 n=10 | | | 90.17±7.70 n=10 | | | 0.79 | | |  |  |
| **u** | AST in rat serum (U/L) | | | | 110.28±6.86 n=9 | | | 118.46±9.48 n=9 | | | 0.49 | | |  |  |

| **Fig. 3** | **Statistical index** | **Vehicle** | **A2M** | ***p* value** |
| --- | --- | --- | --- | --- |
| **h1** | Lt ut-PI | 1.29±0.05 n=10 | 1.61±0.03 n=10 | ＜0.001 |
| **h2** | Lt ut-RI | 0.70±0.02 n=10 | 0.79±0.02 n=10 | 0.002 |
| **i1** | IHC scores of α-SMA | 3.33±0.33 n=6 | 4.67±0.49 n=6 | 0.04 |
| **i2** | Ratio of un-remodeled blood vessels (%) | 28.00±2.16 n=8 | 41.63±1.91 n=8 | ＜0.001 |
| **j1** | Relative protein level of α-SMA | 1.01±0.10 n=7 | 1.35±0.09 n=7 | 0.025 |

| **Fig. 6** | **Statistical index** | **Vehicle** | **A2M** | ***p* value** |
| --- | --- | --- | --- | --- |
| **a1** | Labyrinth area of placenta (%) | 74.71±3.16 n=5 | 65.75±1.41 n=4 | 0.04 |
| **a2** | Blood sinusoids in the labyrinthine (%) | 10.42±0.64 n=19 | 5.70±0.54 n=15 | ＜0.001 |
| **b1** | Area of A2M positive expression (%) | 1.93±0.15 n=9 | 2.43±0.16 n=9 | 0.04 |
| **b2** | Area of Caveolin1 positive expression (%) | 7.46±0.35 n=19 | 5.30±0.26 n=12 | ＜0.001 |
| **c1** | Relative protein level of A2M | 0.46±0.08 n=7 | 0.82±0.05 n=7 | 0.002 |
| **d1** | Relative protein level of Caveolin1 | 0.54±0.05 n=3 | 0.19±0.09 n=3 | 0.03 |
| **e1** | Relative protein level of VEGF | 1.04±0.11 n=8 | 0.59±0.08 n=8 | 0.007 |
| **f1** | Relative protein level of CD31 | 0.56±0.01 n=3 | 0.20±0.07 n=3 | 0.07 |

| **Fig. 8** | **Statistical index** | | **Vehicle** | **A2M** | | ***p* value** | |  |
| --- | --- | --- | --- | --- | --- | --- | --- | --- |
| **e** | Rat serum sFLT-1 level (ng/ml) | GD 7.5 | 2.40±0.18 n=8 | 2.54±0.18 n=8 | | 0.61 | |  |
|  |  | GD 19.5 | 2.85±0.15 n=8 | 4.19±0.29 n=9 | | ＜0.001 | |  |
| **f** | Rat serum PIGF level (pg/ml) | GD 7.5 | 76.35±4.36 n=8 | | 61.005±6.05 n=8 | | 0.05 | |
|  |  | GD 19.5 | 77.83±5.04 n=8 | | 38.63±4.67 n=7 | | ＜0.001 | |

| **Fig. S1** | **Statistical index** | **Vehicle** | **A2M** | ***p* value** |
| --- | --- | --- | --- | --- |
| **b** | number of fetuses | 17.17±0.51 n=12 | 14.67±0.57 n=12 | 0.003 |
| **d** | fetal weight (g) | 4.25±0.09 n=22 | 3.45±0.11 n=22 | ＜0.001 |
| **e** | crown-rump length (cm) | 3.69±0.03 n=47 | 3.11±0.05 n=47 | ＜0.001 |
| **f** | placental weight (g) | 0.57±0.02 n=47 | 0.49±0.01 n=47 | ＜0.001 |
| **g** | placental diameter (cm) | 1.41±0.01 n=43 | 1.40±0.02 n=35 | 0.44 |

| **Fig. S7** | | **Statistical index** | | **Vehicle** | | **A2M** | | ***p* value** | |
| --- | --- | --- | --- | --- | --- | --- | --- | --- | --- |
| **f** | Angiotensinogen level (μg/ml) | | 3.09±0.82 n=6 | | 2.22±0.32 n=6 | | 0.36 | |  |
| **g** | AngⅡlevel (pg/ml) | | 10.07±1.10 n=11 | | 6.15±1.24 n=12 | | 0.03 | |  |
| **h** | ATIR level (ng/ml) | | 5.92±0.33 n=12 | | 6.45±0.42 n=12 | | 0.32 | |  |
| **i** | Ang-(1-7) level (ng/ml) | | 3.82±0.21 n-12 | | 3.02±0.25 n=12 | | 0.02 | |  |
| **j** | AT1-Aas level (ng/ml) | | 13.91±1.57 n=8 | | 20.41±1.58 n=8 | | 0.01 | |  |

# *Supplementary Results*

## *Supplementary Result 1. A2M sequencing result*

ATTCTATTGGCTGAGCTGCGTTCTACGTGGGTATAAGAGGCGCGACCAGCGTCGGTACCGTCGCAGTCTTCGGTCTGACCACCGTAGAACGCAGATCAGATCTCGAGCTCAAGCTTCGAATTCGCCACCATGGGGAAGAACAAACTCCTTCATCCAAGTCTGGTTCTTCTCCTCTTGGTCCTCCTGCCCACAGACGCCTCAGTCTCTGGAAAACCGCAGTATATGGTTCTGGTCCCCTCCCTGCTCCACACTGAGACCACTGAGAAGGGCTGTGTCCTTCTGAGCTACCTGAATGAGACAGTGACTGTAAGTGCTTCCTTGGAGTCTGTCAGGGGAAACAGGAGCCTCTTCACTGACCTGGAGGCGGAGAATGACGTACTCCACTGTGTCGCCTTCGCTGTCCCAAAGTCTTCATCCAATGAGGAGGTAATGTTCCTCACTGTCCAAGTGAAAGGACCAACCCAAGAATTTAAGAAGCGGACCACAGTGATGGTTAAGAACGAGGACAGTCTGGTCTTTGTCCAGACAGACAAATCAATCTACAAACCAGGGCAGACAGTGAAATTTCGTGTTGTCTCCATGGATGAAAACTTTCACCCCCTGAATGAGTTGATTCCACTAGTATACATTCAGGATCCCAAAGGAAATCGCATCGCACAATGGCAGAGTTTCCAGTTAGAGGGTGGCCTCAAGCAATTTTCTTTTCCCCTCTCATCAGAGCCCTTCCAGGGCTCCTACAAGGTGGTGGTACAGAAGAAATCAGGTGGAAGGACAGAGCACCCTTTCACCGTGGAGGAATTTGTTCTTCCCAAGTTTGAAGTACAAGTAACAGTGCCAAAGATAATCACCATCTTGGAAGAAGAGATGAATGTATCAGTGTGTGGCCTATACACATATGGGAAGCCTGTCCCTGGACATGTGACTGTGAGCATTTGCAGAAAGTATAGTGACGCTTCCGACTGCCACGGTGAAGATTCACAGGCTTTCTGTGAGAAATTCAGTGGACAGCTAAACAGCCATGGCTGCTTCTATCAGCAAGTAAAAACCAAGGTCTTCCAGCTGAAGAGGAAGGAGTATGAAATGAAACTTCACACTGAGGCCCAGATCCAAGAAGAAGGAACAGTGGTGGAATTGACTGGAAGGCAGTCCAGTGAAATCACAAGAACCATAACCAAACTCTCATTTGTGAAAGTGGACTCACACTTTCGACAGGGAATTCCCTTCTTTGGGCAGGTGCGCCTAGTAGATGGGAAAGGCGTCCCTATACCAAATAAAGTCATATTCATCAGAGGAAATGAAGCAAACTATTACTCCAATGCTACCACGGATGAGCATGGCCTTGTACAGTTCTCTATCAACACCACCAATGTTATGGGTACCTCTCTTACTGTTAGGGTCAATTACAAGGATCGTAGTCCCTGTTACGGCTACCAGTGGGTGTCAGAAGAACACGAAGAGGCACATCACACTGCTTATCTTGTGTTCTCCCCAAGCAAGAGCTTTGTCCACCTTGAGCCCATGTCTCATGAACTACCCTGTGGCCATACTCAGACAGTCCAGGCACATTATATTCTGAATGGAGGCACCCTGCTGGGGCTGAAGAAGCTCTCCTTCTATTATCTGATAATGGCAAAGGGAGGCATTGTCCGAACTGGGACTCATGGACTGCTTGTGAAGCAGGAAGACATGAAGGGCCATTTTTCCATCTCAATCCCTGTGAAGTCAGACATTGCTCCTGTCGCTCGGTTGCTCATCTATGCTGTTTTACCTACCGGGGACGTGATTGGGGATTCTGCAAAATATGATGTTGAAAATTGTCTGGCCAACAAGGTGGATTTGAGCTTCAGCCCATCACAAAGTCTCCCAGCCTCACACGCCCACCTGCGAGTCACAGCGGCTCCTCAGTCCGTCTGCGCCCTCCGTGCTGTGGACCAAAGCGTGCTGCTCATGAAGCCTGATGCTGAGCTCTCGGCGTCCTCGGTTTACAACCTGCTACCAGAAAAGGACCTCACTGGCTTCCCTGGGCCTTTGAATGACCAGGACGATGAAGACTGCATCAATCGTCATAATGTCTATATTAATGGAATCACATATACTCCAGTATCAAGTACAAATGAAAAGGATATGTACAGCTTCCTAGAGGACATGGGCTTAAAGGCATTCACCAACTCAAAGATTCGTAAACCCAAAATGTGTCCACAGCTTCAACAGTATGAAATGCATGGACCTGAAGGTCTACGTGTAGGTTTTTATGAGTCAGATGTAATGGGAAGAGGCCATGCACGCCTGGTGCATGTTGAAGAGCCTCACACGGAGACCGTACGAAAGTACTTCCCTGAGACATGGATCTGGGATTTGGTGGTGGTAAACTCAGCAGGTGTGGCTGAGGTAGGAGTAACAGTCCCTGACACCATCACCGAGTGGAAGGCAGGGGCCTTCTGCCTGTCTGAAGATGCTGGACTTGGTATCTCTTCCACTGCCTCTCTCCGAGCCTTCCAGCCCTTCTTTGTGGAGCTCACAATGCCTTACTCTGTGATTCGTGGAGAGGCCTTCACACTCAAGGCCACGGTCCTAAACTACCTTCCCAAATGCATCCGGGTCAGTGTGCAGCTGGAAGCCTCTCCCGCCTTCCTAGCTGTCCCAGTGGAGAAGGAACAAGCGCCTCACTGCATCTGTGCAAACGGGCGGCAAACTGTGTCCTGGGCAGTAACCCCAAAGTCATTAGGAAATGTGAATTTCACTGTGAGCGCAGAGGCACTAGAGTCTCAAGAGCTGTGTGGGACTGAGGTGCCTTCAGTTCCTGAACACGGAAGGAAAGACACAGTCATCAAGCCTCTGTTGGTTGAACCTGAAGGACTAGAGAAGGAAACAACATTCAACTCCCTACTTTGTCCATCAGGTGGTGAGGTTTCTGAAGAATTATCCCTGAAACTGCCACCAAATGTGGTAGAAGAATCTGCCCGAGCTTCTGTCTCAGTTTTGGGAGACATATTAGGCTCTGCCATGCAAAACACACAAAATCTTCTCCAGATGCCCTATGGCTGTGGAGAGCAGAATATGGTCCTCTTTGCTCCTAACATCTATGTACTGGATTATCTAAATGAAACACAGCAGCTTACTCCAGAGATCAAGTCCAAGGCCATTGGCTATCTCAACACTGGTTACCAGAGACAGTTGAACTACAAACACTATGATGGCTCCTACAGCACCTTTGGGGAGCGATATGGCAGGAACCAGGGCAACACCTGGCTCACAGCCTTTGTTCTGAAGACTTTTGCCCAAGCTCGAGCCTACATCTTCATCGATGAAGCACACATTACCCAAGCCCTCATATGGCTCTCCCAGAGGCAGAAGGACAATGGCTGTTTCAGGAGCTCTGGGTCACTGCTCAACAATGCCATAAAGGGAGGAGTAGAAGATGAAGTGACCCTCTCCGCCTATATCACCATCGCCCTTCTGGAGATTCCTCTCACAGTCACTCACCCTGTTGTCCGCAATGCCCTGTTTTGCCTGGAGTCAGCCTGGAAGACAGCACAAGAAGGGGACCATGGCAGCCATGTATATACCAAAGCACTGCTGGCCTATGCTTTTGCCCTGGCAGGTAACCAGGACAAGAGGAAGGAAGTACTCAAGTCACTTAATGAGGAAGCTGTGAAGAAAGACAACTCTGTCCATTGGGAGCGCCCTCAGAAACCCAAGGCACCAGTGGGGCATTTTTACGAACCCCAGGCTCCCTCTGCTGAGGTGGAGATGACATCCTATGTGCTCCTCGCTTATCTCACGGCCCAGCCAGCCCCAACCTCGGAGGACCTGACCTCTGCAACCAACATCGTGAAGTGGATCACGAAGCAGCAGAATGCCCAGGGCGGTTTCTCCTCCACCCAGGACACAGTGGTGGCTCTCCATGCTCTGTCCAAATATGGAGCAGCCACATTTACCAGGACTGGGAAGGCTGCACAGGTGACTATCCAGTCTTCAGGGACATTTTCCAGCAAATTCCAAGTGGACAACAACAACCGCCTGTTACTGCAGCAGGTCTCATTGCCAGAGCTGCCTGGGGAATACAGCATGAAAGTGACAGGAGAAGGATGTGTCTACCTCCAGACATCCTTGAAATACAATATTCTCCCAGAAAAGGAAGAGTTCCCCTTTGCTTTAGGAGTGCAGACTCTGCCTCAAACTTGTGATGAACCCAAAGCCCACACCAGCTTCCAAATCTCCCTAAGTGTCAGTTACACAGGGAGCCGCTCTGCCTCCAACATGGCGATCGTTGATGTGAAGATGGTCTCTGGCTTCATTCCCCTGAAGCCAACAGTGAAAATGCTTGAAAGATCTAACCATGTGAGCCGGACAGAAGTCAGCAGCAACCATGTCTTGATTTACCTTGATAAGGTGTCAAATCAGACACTGAGCTTGTTCTTCACGGTTCTGCAAGATGTCCCAGTAAGAGATCTGAAACCAGCCATAGTGAAAGTCTATGATTACTACGAGACGGATGAGTTTGCAATTGCTGAGTACAATGCTCCTTGCAGCAAAGATCTTGGAAATGCTGAATTCGCCACCATGGACTACAAGGATGACGATGACAAGGATTACAAAGACGACGATGATAAGGACTATAAGGATGATGACGACAAAGGATCCGGAAGCGGAGCTACTAACTTC

1 50

seq (1) ATTCTATTGGCTGAGCTGCGTTCTACGTGGGTATAAGAGGCGCGACCAGC

A2M (1) --------------------------------------------------

51 100

seq (51) GTCGGTACCGTCGCAGTCTTCGGTCTGACCACCGTAGAACGCAGATCAGA

A2M (1) --------------------------------------------------

101 150

seq (101) TCTCGAGCTCAAGCTTCGAATTCGCCACCATGGGGAAGAACAAACTCCTT

A2M (1) -----------------------------ATGGGGAAGAACAAACTCCTT

151 200

seq (151) CATCCAAGTCTGGTTCTTCTCCTCTTGGTCCTCCTGCCCACAGACGCCTC

A2M (22) CATCCAAGTCTGGTTCTTCTCCTCTTGGTCCTCCTGCCCACAGACGCCTC

201 250

seq (201) AGTCTCTGGAAAACCGCAGTATATGGTTCTGGTCCCCTCCCTGCTCCACA

A2M (72) AGTCTCTGGAAAACCGCAGTATATGGTTCTGGTCCCCTCCCTGCTCCACA

251 300

seq (251) CTGAGACCACTGAGAAGGGCTGTGTCCTTCTGAGCTACCTGAATGAGACA

A2M (122) CTGAGACCACTGAGAAGGGCTGTGTCCTTCTGAGCTACCTGAATGAGACA

301 350

seq (301) GTGACTGTAAGTGCTTCCTTGGAGTCTGTCAGGGGAAACAGGAGCCTCTT

A2M (172) GTGACTGTAAGTGCTTCCTTGGAGTCTGTCAGGGGAAACAGGAGCCTCTT

351 400

seq (351) CACTGACCTGGAGGCGGAGAATGACGTACTCCACTGTGTCGCCTTCGCTG

A2M (222) CACTGACCTGGAGGCGGAGAATGACGTACTCCACTGTGTCGCCTTCGCTG

401 450

seq (401) TCCCAAAGTCTTCATCCAATGAGGAGGTAATGTTCCTCACTGTCCAAGTG

A2M (272) TCCCAAAGTCTTCATCCAATGAGGAGGTAATGTTCCTCACTGTCCAAGTG

451 500

seq (451) AAAGGACCAACCCAAGAATTTAAGAAGCGGACCACAGTGATGGTTAAGAA

A2M (322) AAAGGACCAACCCAAGAATTTAAGAAGCGGACCACAGTGATGGTTAAGAA

501 550

seq (501) CGAGGACAGTCTGGTCTTTGTCCAGACAGACAAATCAATCTACAAACCAG

A2M (372) CGAGGACAGTCTGGTCTTTGTCCAGACAGACAAATCAATCTACAAACCAG

551 600

seq (551) GGCAGACAGTGAAATTTCGTGTTGTCTCCATGGATGAAAACTTTCACCCC

A2M (422) GGCAGACAGTGAAATTTCGTGTTGTCTCCATGGATGAAAACTTTCACCCC

601 650

seq (601) CTGAATGAGTTGATTCCACTAGTATACATTCAGGATCCCAAAGGAAATCG

A2M (472) CTGAATGAGTTGATTCCACTAGTATACATTCAGGATCCCAAAGGAAATCG

651 700

seq (651) CATCGCACAATGGCAGAGTTTCCAGTTAGAGGGTGGCCTCAAGCAATTTT

A2M (522) CATCGCACAATGGCAGAGTTTCCAGTTAGAGGGTGGCCTCAAGCAATTTT

701 750

seq (701) CTTTTCCCCTCTCATCAGAGCCCTTCCAGGGCTCCTACAAGGTGGTGGTA

A2M (572) CTTTTCCCCTCTCATCAGAGCCCTTCCAGGGCTCCTACAAGGTGGTGGTA

751 800

seq (751) CAGAAGAAATCAGGTGGAAGGACAGAGCACCCTTTCACCGTGGAGGAATT

A2M (622) CAGAAGAAATCAGGTGGAAGGACAGAGCACCCTTTCACCGTGGAGGAATT

801 850

seq (801) TGTTCTTCCCAAGTTTGAAGTACAAGTAACAGTGCCAAAGATAATCACCA

A2M (672) TGTTCTTCCCAAGTTTGAAGTACAAGTAACAGTGCCAAAGATAATCACCA

851 900

seq (851) TCTTGGAAGAAGAGATGAATGTATCAGTGTGTGGCCTATACACATATGGG

A2M (722) TCTTGGAAGAAGAGATGAATGTATCAGTGTGTGGCCTATACACATATGGG

901 950

seq (901) AAGCCTGTCCCTGGACATGTGACTGTGAGCATTTGCAGAAAGTATAGTGA

A2M (772) AAGCCTGTCCCTGGACATGTGACTGTGAGCATTTGCAGAAAGTATAGTGA

951 1000

seq (951) CGCTTCCGACTGCCACGGTGAAGATTCACAGGCTTTCTGTGAGAAATTCA

A2M (822) CGCTTCCGACTGCCACGGTGAAGATTCACAGGCTTTCTGTGAGAAATTCA

1001 1050

seq (1001) GTGGACAGCTAAACAGCCATGGCTGCTTCTATCAGCAAGTAAAAACCAAG

A2M (872) GTGGACAGCTAAACAGCCATGGCTGCTTCTATCAGCAAGTAAAAACCAAG

1051 1100

seq (1051) GTCTTCCAGCTGAAGAGGAAGGAGTATGAAATGAAACTTCACACTGAGGC

A2M (922) GTCTTCCAGCTGAAGAGGAAGGAGTATGAAATGAAACTTCACACTGAGGC

1101 1150

seq (1101) CCAGATCCAAGAAGAAGGAACAGTGGTGGAATTGACTGGAAGGCAGTCCA

A2M (972) CCAGATCCAAGAAGAAGGAACAGTGGTGGAATTGACTGGAAGGCAGTCCA

1151 1200

seq (1151) GTGAAATCACAAGAACCATAACCAAACTCTCATTTGTGAAAGTGGACTCA

A2M (1022) GTGAAATCACAAGAACCATAACCAAACTCTCATTTGTGAAAGTGGACTCA

1201 1250

seq (1201) CACTTTCGACAGGGAATTCCCTTCTTTGGGCAGGTGCGCCTAGTAGATGG

A2M (1072) CACTTTCGACAGGGAATTCCCTTCTTTGGGCAGGTGCGCCTAGTAGATGG

1251 1300

seq (1251) GAAAGGCGTCCCTATACCAAATAAAGTCATATTCATCAGAGGAAATGAAG

A2M (1122) GAAAGGCGTCCCTATACCAAATAAAGTCATATTCATCAGAGGAAATGAAG

1301 1350

seq (1301) CAAACTATTACTCCAATGCTACCACGGATGAGCATGGCCTTGTACAGTTC

A2M (1172) CAAACTATTACTCCAATGCTACCACGGATGAGCATGGCCTTGTACAGTTC

1351 1400

seq (1351) TCTATCAACACCACCAATGTTATGGGTACCTCTCTTACTGTTAGGGTCAA

A2M (1222) TCTATCAACACCACCAATGTTATGGGTACCTCTCTTACTGTTAGGGTCAA

1401 1450

seq (1401) TTACAAGGATCGTAGTCCCTGTTACGGCTACCAGTGGGTGTCAGAAGAAC

A2M (1272) TTACAAGGATCGTAGTCCCTGTTACGGCTACCAGTGGGTGTCAGAAGAAC

1451 1500

seq (1451) ACGAAGAGGCACATCACACTGCTTATCTTGTGTTCTCCCCAAGCAAGAGC

A2M (1322) ACGAAGAGGCACATCACACTGCTTATCTTGTGTTCTCCCCAAGCAAGAGC

1501 1550

seq (1501) TTTGTCCACCTTGAGCCCATGTCTCATGAACTACCCTGTGGCCATACTCA

A2M (1372) TTTGTCCACCTTGAGCCCATGTCTCATGAACTACCCTGTGGCCATACTCA

1551 1600

seq (1551) GACAGTCCAGGCACATTATATTCTGAATGGAGGCACCCTGCTGGGGCTGA

A2M (1422) GACAGTCCAGGCACATTATATTCTGAATGGAGGCACCCTGCTGGGGCTGA

1601 1650

seq (1601) AGAAGCTCTCCTTCTATTATCTGATAATGGCAAAGGGAGGCATTGTCCGA

A2M (1472) AGAAGCTCTCCTTCTATTATCTGATAATGGCAAAGGGAGGCATTGTCCGA

1651 1700

seq (1651) ACTGGGACTCATGGACTGCTTGTGAAGCAGGAAGACATGAAGGGCCATTT

A2M (1522) ACTGGGACTCATGGACTGCTTGTGAAGCAGGAAGACATGAAGGGCCATTT

1701 1750

seq (1701) TTCCATCTCAATCCCTGTGAAGTCAGACATTGCTCCTGTCGCTCGGTTGC

A2M (1572) TTCCATCTCAATCCCTGTGAAGTCAGACATTGCTCCTGTCGCTCGGTTGC

1751 1800

seq (1751) TCATCTATGCTGTTTTACCTACCGGGGACGTGATTGGGGATTCTGCAAAA

A2M (1622) TCATCTATGCTGTTTTACCTACCGGGGACGTGATTGGGGATTCTGCAAAA

1801 1850

seq (1801) TATGATGTTGAAAATTGTCTGGCCAACAAGGTGGATTTGAGCTTCAGCCC

A2M (1672) TATGATGTTGAAAATTGTCTGGCCAACAAGGTGGATTTGAGCTTCAGCCC

1851 1900

seq (1851) ATCACAAAGTCTCCCAGCCTCACACGCCCACCTGCGAGTCACAGCGGCTC

A2M (1722) ATCACAAAGTCTCCCAGCCTCACACGCCCACCTGCGAGTCACAGCGGCTC

1901 1950

seq (1901) CTCAGTCCGTCTGCGCCCTCCGTGCTGTGGACCAAAGCGTGCTGCTCATG

A2M (1772) CTCAGTCCGTCTGCGCCCTCCGTGCTGTGGACCAAAGCGTGCTGCTCATG

1951 2000

seq (1951) AAGCCTGATGCTGAGCTCTCGGCGTCCTCGGTTTACAACCTGCTACCAGA

A2M (1822) AAGCCTGATGCTGAGCTCTCGGCGTCCTCGGTTTACAACCTGCTACCAGA

2001 2050

seq (2001) AAAGGACCTCACTGGCTTCCCTGGGCCTTTGAATGACCAGGACGATGAAG

A2M (1872) AAAGGACCTCACTGGCTTCCCTGGGCCTTTGAATGACCAGGACGATGAAG

2051 2100

seq (2051) ACTGCATCAATCGTCATAATGTCTATATTAATGGAATCACATATACTCCA

A2M (1922) ACTGCATCAATCGTCATAATGTCTATATTAATGGAATCACATATACTCCA

2101 2150

seq (2101) GTATCAAGTACAAATGAAAAGGATATGTACAGCTTCCTAGAGGACATGGG

A2M (1972) GTATCAAGTACAAATGAAAAGGATATGTACAGCTTCCTAGAGGACATGGG

2151 2200

seq (2151) CTTAAAGGCATTCACCAACTCAAAGATTCGTAAACCCAAAATGTGTCCAC

A2M (2022) CTTAAAGGCATTCACCAACTCAAAGATTCGTAAACCCAAAATGTGTCCAC

2201 2250

seq (2201) AGCTTCAACAGTATGAAATGCATGGACCTGAAGGTCTACGTGTAGGTTTT

A2M (2072) AGCTTCAACAGTATGAAATGCATGGACCTGAAGGTCTACGTGTAGGTTTT

2251 2300

seq (2251) TATGAGTCAGATGTAATGGGAAGAGGCCATGCACGCCTGGTGCATGTTGA

A2M (2122) TATGAGTCAGATGTAATGGGAAGAGGCCATGCACGCCTGGTGCATGTTGA

2301 2350

seq (2301) AGAGCCTCACACGGAGACCGTACGAAAGTACTTCCCTGAGACATGGATCT

A2M (2172) AGAGCCTCACACGGAGACCGTACGAAAGTACTTCCCTGAGACATGGATCT

2351 2400

seq (2351) GGGATTTGGTGGTGGTAAACTCAGCAGGTGTGGCTGAGGTAGGAGTAACA

A2M (2222) GGGATTTGGTGGTGGTAAACTCAGCAGGTGTGGCTGAGGTAGGAGTAACA

2401 2450

seq (2401) GTCCCTGACACCATCACCGAGTGGAAGGCAGGGGCCTTCTGCCTGTCTGA

A2M (2272) GTCCCTGACACCATCACCGAGTGGAAGGCAGGGGCCTTCTGCCTGTCTGA

2451 2500

seq (2451) AGATGCTGGACTTGGTATCTCTTCCACTGCCTCTCTCCGAGCCTTCCAGC

A2M (2322) AGATGCTGGACTTGGTATCTCTTCCACTGCCTCTCTCCGAGCCTTCCAGC

2501 2550

seq (2501) CCTTCTTTGTGGAGCTCACAATGCCTTACTCTGTGATTCGTGGAGAGGCC

A2M (2372) CCTTCTTTGTGGAGCTCACAATGCCTTACTCTGTGATTCGTGGAGAGGCC

2551 2600

seq (2551) TTCACACTCAAGGCCACGGTCCTAAACTACCTTCCCAAATGCATCCGGGT

A2M (2422) TTCACACTCAAGGCCACGGTCCTAAACTACCTTCCCAAATGCATCCGGGT

2601 2650

seq (2601) CAGTGTGCAGCTGGAAGCCTCTCCCGCCTTCCTAGCTGTCCCAGTGGAGA

A2M (2472) CAGTGTGCAGCTGGAAGCCTCTCCCGCCTTCCTAGCTGTCCCAGTGGAGA

2651 2700

seq (2651) AGGAACAAGCGCCTCACTGCATCTGTGCAAACGGGCGGCAAACTGTGTCC

A2M (2522) AGGAACAAGCGCCTCACTGCATCTGTGCAAACGGGCGGCAAACTGTGTCC

2701 2750

seq (2701) TGGGCAGTAACCCCAAAGTCATTAGGAAATGTGAATTTCACTGTGAGCGC

A2M (2572) TGGGCAGTAACCCCAAAGTCATTAGGAAATGTGAATTTCACTGTGAGCGC

2751 2800

seq (2751) AGAGGCACTAGAGTCTCAAGAGCTGTGTGGGACTGAGGTGCCTTCAGTTC

A2M (2622) AGAGGCACTAGAGTCTCAAGAGCTGTGTGGGACTGAGGTGCCTTCAGTTC

2801 2850

seq (2801) CTGAACACGGAAGGAAAGACACAGTCATCAAGCCTCTGTTGGTTGAACCT

A2M (2672) CTGAACACGGAAGGAAAGACACAGTCATCAAGCCTCTGTTGGTTGAACCT

2851 2900

seq (2851) GAAGGACTAGAGAAGGAAACAACATTCAACTCCCTACTTTGTCCATCAGG

A2M (2722) GAAGGACTAGAGAAGGAAACAACATTCAACTCCCTACTTTGTCCATCAGG

2901 2950

seq (2901) TGGTGAGGTTTCTGAAGAATTATCCCTGAAACTGCCACCAAATGTGGTAG

A2M (2772) TGGTGAGGTTTCTGAAGAATTATCCCTGAAACTGCCACCAAATGTGGTAG

2951 3000

seq (2951) AAGAATCTGCCCGAGCTTCTGTCTCAGTTTTGGGAGACATATTAGGCTCT

A2M (2822) AAGAATCTGCCCGAGCTTCTGTCTCAGTTTTGGGAGACATATTAGGCTCT

3001 3050

seq (3001) GCCATGCAAAACACACAAAATCTTCTCCAGATGCCCTATGGCTGTGGAGA

A2M (2872) GCCATGCAAAACACACAAAATCTTCTCCAGATGCCCTATGGCTGTGGAGA

3051 3100

seq (3051) GCAGAATATGGTCCTCTTTGCTCCTAACATCTATGTACTGGATTATCTAA

A2M (2922) GCAGAATATGGTCCTCTTTGCTCCTAACATCTATGTACTGGATTATCTAA

3101 3150

seq (3101) ATGAAACACAGCAGCTTACTCCAGAGATCAAGTCCAAGGCCATTGGCTAT

A2M (2972) ATGAAACACAGCAGCTTACTCCAGAGATCAAGTCCAAGGCCATTGGCTAT

3151 3200

seq (3151) CTCAACACTGGTTACCAGAGACAGTTGAACTACAAACACTATGATGGCTC

A2M (3022) CTCAACACTGGTTACCAGAGACAGTTGAACTACAAACACTATGATGGCTC

3201 3250

seq (3201) CTACAGCACCTTTGGGGAGCGATATGGCAGGAACCAGGGCAACACCTGGC

A2M (3072) CTACAGCACCTTTGGGGAGCGATATGGCAGGAACCAGGGCAACACCTGGC

3251 3300

seq (3251) TCACAGCCTTTGTTCTGAAGACTTTTGCCCAAGCTCGAGCCTACATCTTC

A2M (3122) TCACAGCCTTTGTTCTGAAGACTTTTGCCCAAGCTCGAGCCTACATCTTC

3301 3350

seq (3301) ATCGATGAAGCACACATTACCCAAGCCCTCATATGGCTCTCCCAGAGGCA

A2M (3172) ATCGATGAAGCACACATTACCCAAGCCCTCATATGGCTCTCCCAGAGGCA

3351 3400

seq (3351) GAAGGACAATGGCTGTTTCAGGAGCTCTGGGTCACTGCTCAACAATGCCA

A2M (3222) GAAGGACAATGGCTGTTTCAGGAGCTCTGGGTCACTGCTCAACAATGCCA

3401 3450

seq (3401) TAAAGGGAGGAGTAGAAGATGAAGTGACCCTCTCCGCCTATATCACCATC

A2M (3272) TAAAGGGAGGAGTAGAAGATGAAGTGACCCTCTCCGCCTATATCACCATC

3451 3500

seq (3451) GCCCTTCTGGAGATTCCTCTCACAGTCACTCACCCTGTTGTCCGCAATGC

A2M (3322) GCCCTTCTGGAGATTCCTCTCACAGTCACTCACCCTGTTGTCCGCAATGC

3501 3550

seq (3501) CCTGTTTTGCCTGGAGTCAGCCTGGAAGACAGCACAAGAAGGGGACCATG

A2M (3372) CCTGTTTTGCCTGGAGTCAGCCTGGAAGACAGCACAAGAAGGGGACCATG

3551 3600

seq (3551) GCAGCCATGTATATACCAAAGCACTGCTGGCCTATGCTTTTGCCCTGGCA

A2M (3422) GCAGCCATGTATATACCAAAGCACTGCTGGCCTATGCTTTTGCCCTGGCA

3601 3650

seq (3601) GGTAACCAGGACAAGAGGAAGGAAGTACTCAAGTCACTTAATGAGGAAGC

A2M (3472) GGTAACCAGGACAAGAGGAAGGAAGTACTCAAGTCACTTAATGAGGAAGC

3651 3700

seq (3651) TGTGAAGAAAGACAACTCTGTCCATTGGGAGCGCCCTCAGAAACCCAAGG

A2M (3522) TGTGAAGAAAGACAACTCTGTCCATTGGGAGCGCCCTCAGAAACCCAAGG

3701 3750

seq (3701) CACCAGTGGGGCATTTTTACGAACCCCAGGCTCCCTCTGCTGAGGTGGAG

A2M (3572) CACCAGTGGGGCATTTTTACGAACCCCAGGCTCCCTCTGCTGAGGTGGAG

3751 3800

seq (3751) ATGACATCCTATGTGCTCCTCGCTTATCTCACGGCCCAGCCAGCCCCAAC

A2M (3622) ATGACATCCTATGTGCTCCTCGCTTATCTCACGGCCCAGCCAGCCCCAAC

3801 3850

seq (3801) CTCGGAGGACCTGACCTCTGCAACCAACATCGTGAAGTGGATCACGAAGC

A2M (3672) CTCGGAGGACCTGACCTCTGCAACCAACATCGTGAAGTGGATCACGAAGC

3851 3900

seq (3851) AGCAGAATGCCCAGGGCGGTTTCTCCTCCACCCAGGACACAGTGGTGGCT

A2M (3722) AGCAGAATGCCCAGGGCGGTTTCTCCTCCACCCAGGACACAGTGGTGGCT

3901 3950

seq (3901) CTCCATGCTCTGTCCAAATATGGAGCAGCCACATTTACCAGGACTGGGAA

A2M (3772) CTCCATGCTCTGTCCAAATATGGAGCAGCCACATTTACCAGGACTGGGAA

3951 4000

seq (3951) GGCTGCACAGGTGACTATCCAGTCTTCAGGGACATTTTCCAGCAAATTCC

A2M (3822) GGCTGCACAGGTGACTATCCAGTCTTCAGGGACATTTTCCAGCAAATTCC

4001 4050

seq (4001) AAGTGGACAACAACAACCGCCTGTTACTGCAGCAGGTCTCATTGCCAGAG

A2M (3872) AAGTGGACAACAACAACCGCCTGTTACTGCAGCAGGTCTCATTGCCAGAG

4051 4100

seq (4051) CTGCCTGGGGAATACAGCATGAAAGTGACAGGAGAAGGATGTGTCTACCT

A2M (3922) CTGCCTGGGGAATACAGCATGAAAGTGACAGGAGAAGGATGTGTCTACCT

4101 4150

seq (4101) CCAGACATCCTTGAAATACAATATTCTCCCAGAAAAGGAAGAGTTCCCCT

A2M (3972) CCAGACATCCTTGAAATACAATATTCTCCCAGAAAAGGAAGAGTTCCCCT

4151 4200

seq (4151) TTGCTTTAGGAGTGCAGACTCTGCCTCAAACTTGTGATGAACCCAAAGCC

A2M (4022) TTGCTTTAGGAGTGCAGACTCTGCCTCAAACTTGTGATGAACCCAAAGCC

4201 4250

seq (4201) CACACCAGCTTCCAAATCTCCCTAAGTGTCAGTTACACAGGGAGCCGCTC

A2M (4072) CACACCAGCTTCCAAATCTCCCTAAGTGTCAGTTACACAGGGAGCCGCTC

4251 4300

seq (4251) TGCCTCCAACATGGCGATCGTTGATGTGAAGATGGTCTCTGGCTTCATTC

A2M (4122) TGCCTCCAACATGGCGATCGTTGATGTGAAGATGGTCTCTGGCTTCATTC

4301 4350

seq (4301) CCCTGAAGCCAACAGTGAAAATGCTTGAAAGATCTAACCATGTGAGCCGG

A2M (4172) CCCTGAAGCCAACAGTGAAAATGCTTGAAAGATCTAACCATGTGAGCCGG

4351 4400

seq (4351) ACAGAAGTCAGCAGCAACCATGTCTTGATTTACCTTGATAAGGTGTCAAA

A2M (4222) ACAGAAGTCAGCAGCAACCATGTCTTGATTTACCTTGATAAGGTGTCAAA

4401 4450

seq (4401) TCAGACACTGAGCTTGTTCTTCACGGTTCTGCAAGATGTCCCAGTAAGAG

A2M (4272) TCAGACACTGAGCTTGTTCTTCACGGTTCTGCAAGATGTCCCAGTAAGAG

4451 4500

seq (4451) ATCTGAAACCAGCCATAGTGAAAGTCTATGATTACTACGAGACGGATGAG

A2M (4322) ATCTGAAACCAGCCATAGTGAAAGTCTATGATTACTACGAGACGGATGAG

4501 4550

seq (4501) TTTGCAATTGCTGAGTACAATGCTCCTTGCAGCAAAGATCTTGGAAATGC

A2M (4372) TTTGCAATTGCTGAGTACAATGCTCCTTGCAGCAAAGATCTTGGAAATGC

4551 4600

seq (4551) TGAATTCGCCACCATGGACTACAAGGATGACGATGACAAGGATTACAAAG

A2M (4422) T-------------------------------------------------

4601 4650

seq (4601) ACGACGATGATAAGGACTATAAGGATGATGACGACAAAGGATCCGGAAGC

A2M (4423) --------------------------------------------------

4651 4665

seq (4651) GGAGCTACTAACTTC

A2M (4423) ---------------

##

## *Supplementary Result 2. The clinical and laboratory characteristics and adverse pregnancy outcomes of pregnant women enrolled in this study*

The clinical and laboratory characteristics of the women with healthy pregnancies and PE are presented in Supplementary Table 1. In total, 52 patients with PE and 53 women with healthy pregnancies were ultimately enrolled in this study. The statistical analysis indicated that there were no significant differences in maternal age, weight, height or platelet count between the two groups, but the body mass index (BMI), systolic blood pressure, diastolic blood pressure and mean arterial pressure (MAP) in the PE group were significantly higher than those in the normal group, and the gestational weeks in the PE group were significantly shorter than those in the normal group. Regarding the condition of the fetus and appendages, we found that pregnant women with early-onset PE were more likely to deliver smaller babies before the expected date of birth. In addition, umbilical cord length and amniotic fluid volume were significantly reduced in pregnant women with early-onset PE compared to healthy pregnant women. The maternal and fetal/neonatal adverse pregnancy outcomes are shown in Supplementary Table 2. We found that the cesarean section rate in the PE group was significantly increased. Regarding pregnancy outcomes and complications of pregnant women, the numbers in pregnant women with early-onset PE were significantly higher than those in healthy pregnant women. For the fetus/neonates, some common adverse outcomes, such as preterm birth, LBW (low birth weight), myocardial damage, PFO (patent foramen ovale), hypoalbuminemia, neonatal anemia, and neonatal hyperbilirubinemia, were significantly increased in the PE group compared to the normal group. These findings suggest that PE does have an adverse effect on maternal health and fetal/neonatal growth.

# *Supplementary Figures*

## *Supplementary Fig. 1*

***
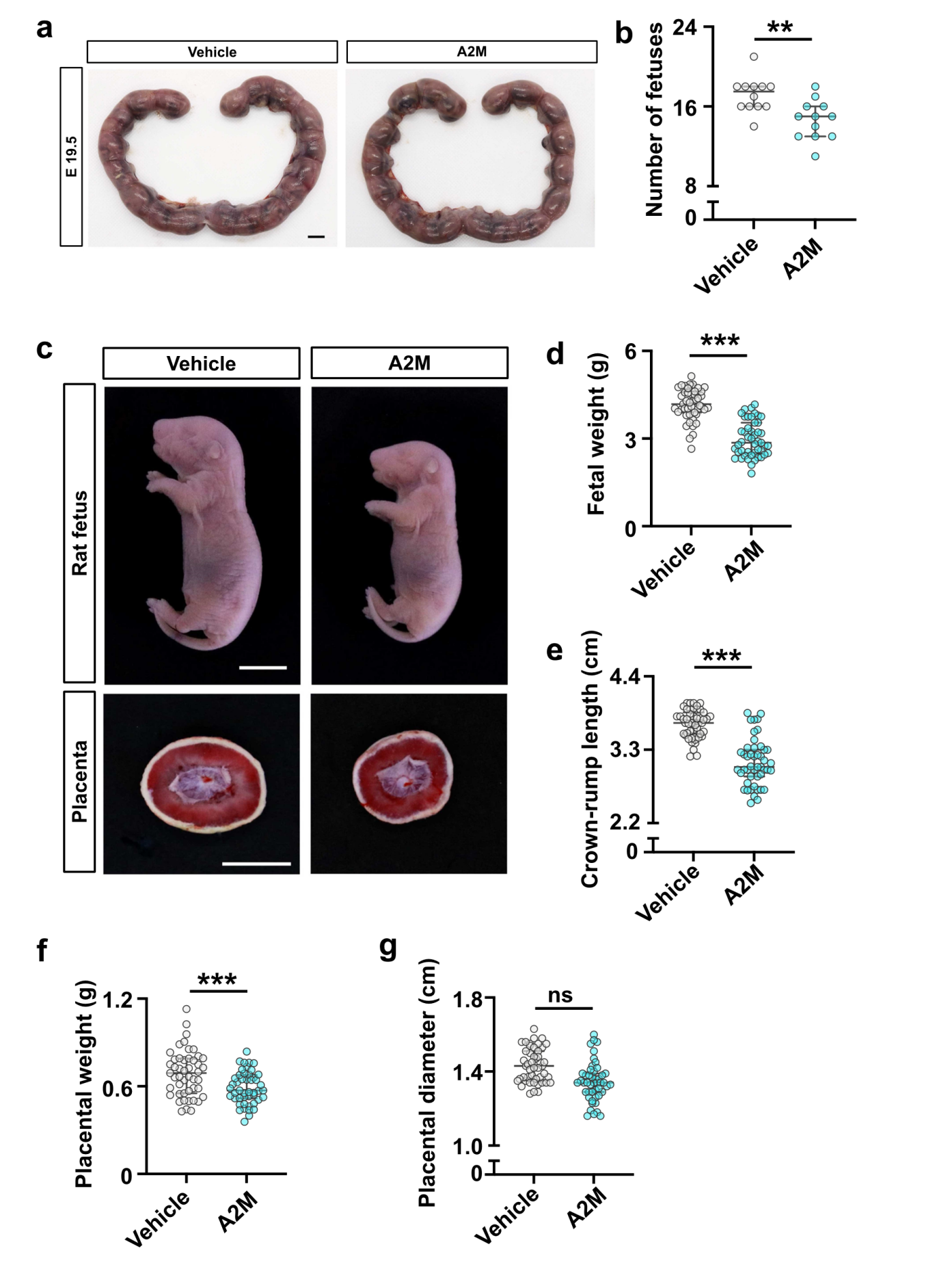
***

***Supplementary Fig. 1. Assessment of placental and fetal development in the A2M-overexpression rat model.***

**a-b**: Representative images of uterine tissues (a) of the control and A2M-overexpression groups and the corresponding quantitative analysis (b) of the number of fetuses. **c-g**: Representative images of the fetus and placenta (c). d-e Quantitative analysis of the fetal weight (d) and crown-rump length (e) of the fetus; f-g Quantitative analysis of the placental weight (f) and placental diameter (g) of the control and A2M-overexpression groups. Scale bars = 1 cm in a, c. **P < 0.01, ***P < 0.001.

## *Supplementary Fig. 2*


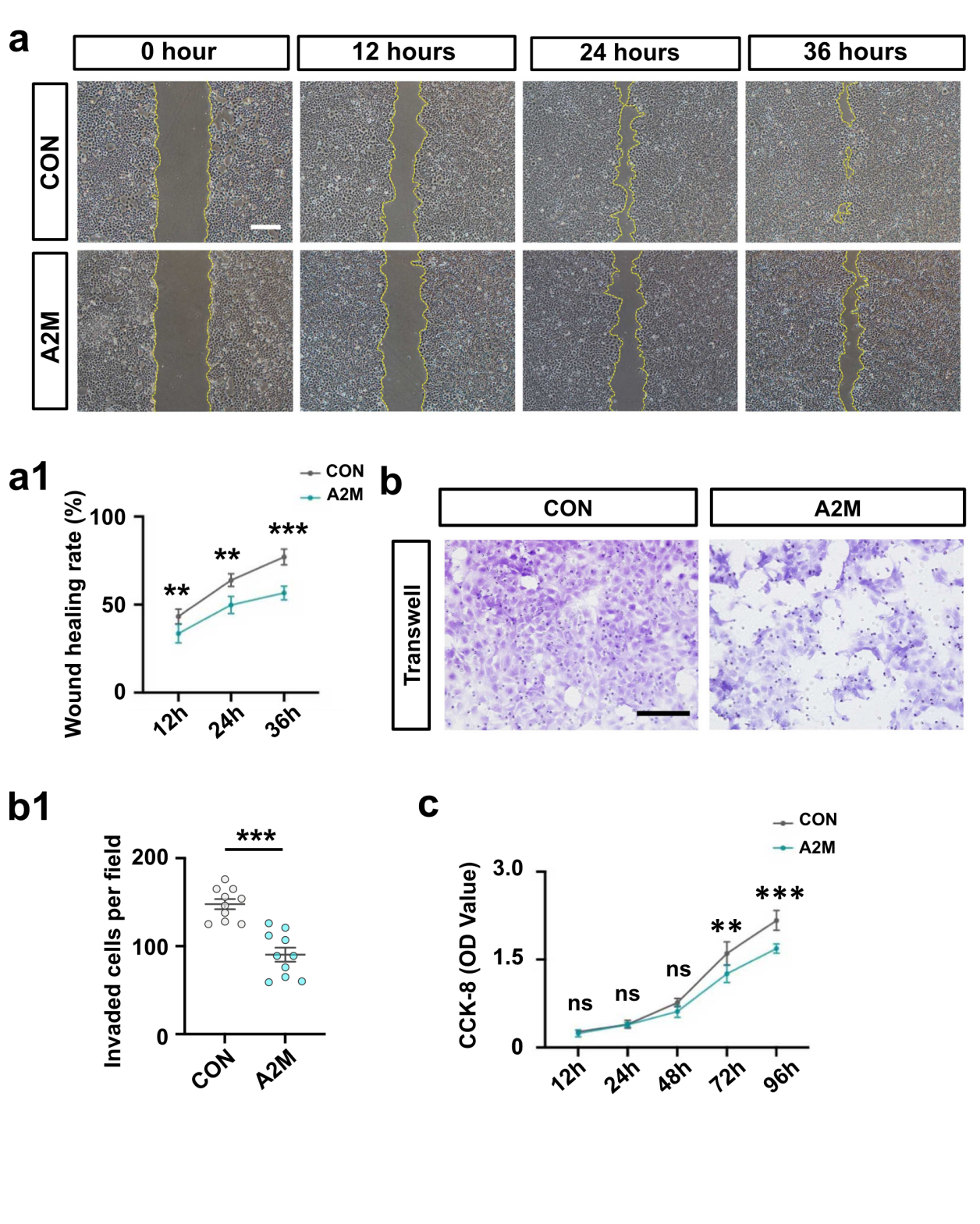


***Supplementary Fig. 2. Determining HTR-8/SVneo cell migration and cell viability following A2M upregulation.***

**a-a1**: Representative images of HTR-8/SVneo cells in the wound-healing assay, which were subjected to scratch wounding after 0, 12, 24, and 36 hours of incubation from the control and A2M-overexpression groups, and a1 shows the quantitative analysis of the wound healing rate in both groups. **b-b1**: Representative images of transwell invasion assays of HTR-8/SVneo cells either transfected with negative control (control) or A2M-overexpression vectors at 48 hours of incubation, and b1 shows the quantitative analysis of the numbers of invaded cells in both groups. **c**: Determination of cell viability in HTR-8/SVneo cells from both groups after 12, 24, 48, 72 and 96 hours of incubation by CCK-8 assay. Scale bars = 100 μm in a; 50 μm in b. **P < 0.01, ***P < 0.001.

## *Supplementary Fig. 3*


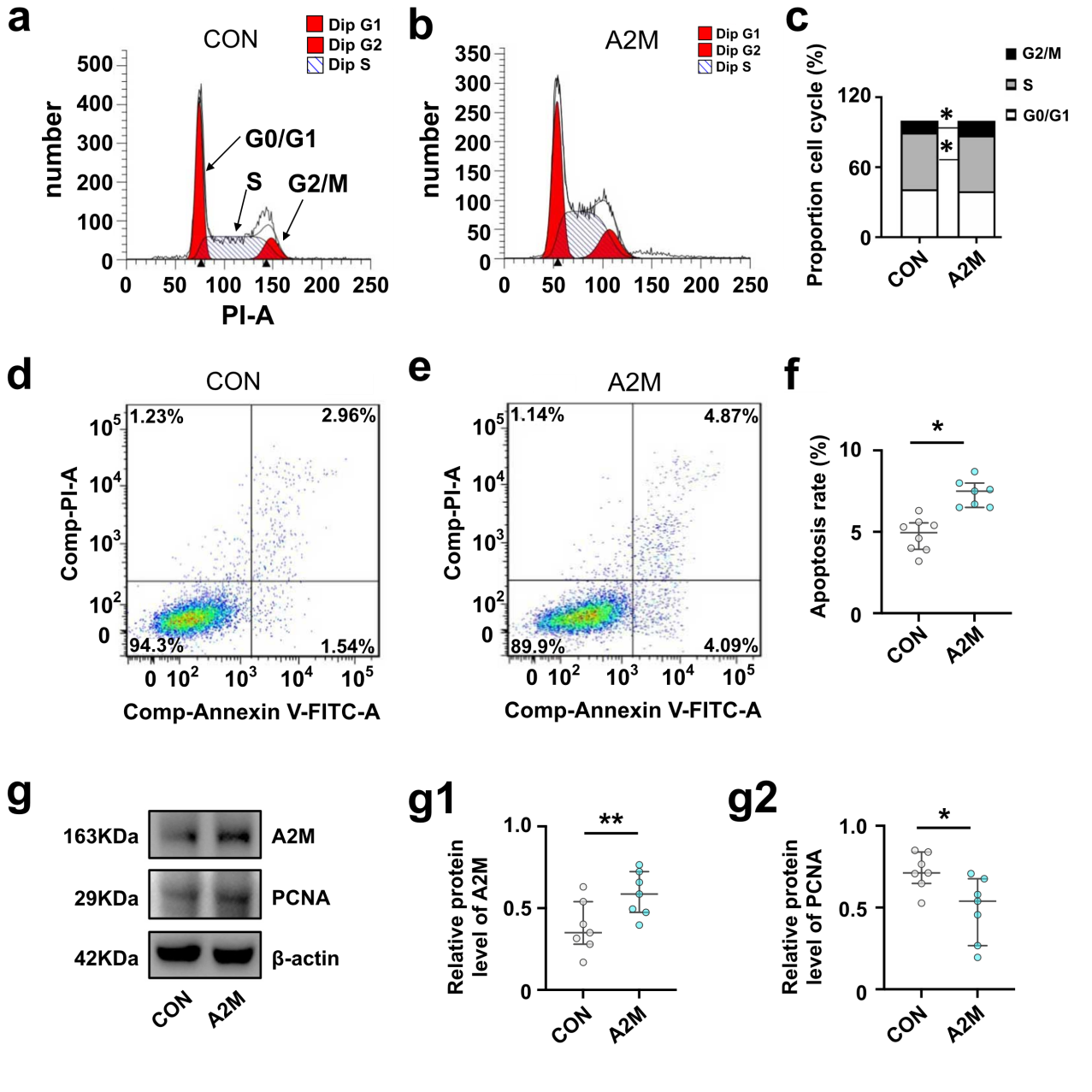


***Supplementary Fig. 3. Determining HTR-8/SVneo cell proliferation and apoptosis following A2M upregulation.***

**a-c**: Flow cytometry data showing the analysis of DNA contents in HTR-8/SVneo cells transfected with either negative control (a) or A2M-overexpression vectors (b), and c shows the quantitative analysis of the proportion of cells in each phase of the cell cycle in the two groups. **d-f**: Apoptosis of HTR-8/SVneo cells transfected with either negative control (d) or A2M-overexpression vectors (e) was determined by flow cytometry using the annexin V-FITC/PI apoptosis assay, and f shows the quantitative analysis of the cell apoptosis rates in the two groups. **g, g1-g2**: Western blotting data showing the expression of A2M and PCNA in HTR-8/SVneo cells from both groups (g), and g1-g2 show the quantitative analyses of A2M (g1) and PCNA (g2) expression in the two groups. *P < 0.05, **P < 0.01.

## *Supplementary Fig. 4*

***
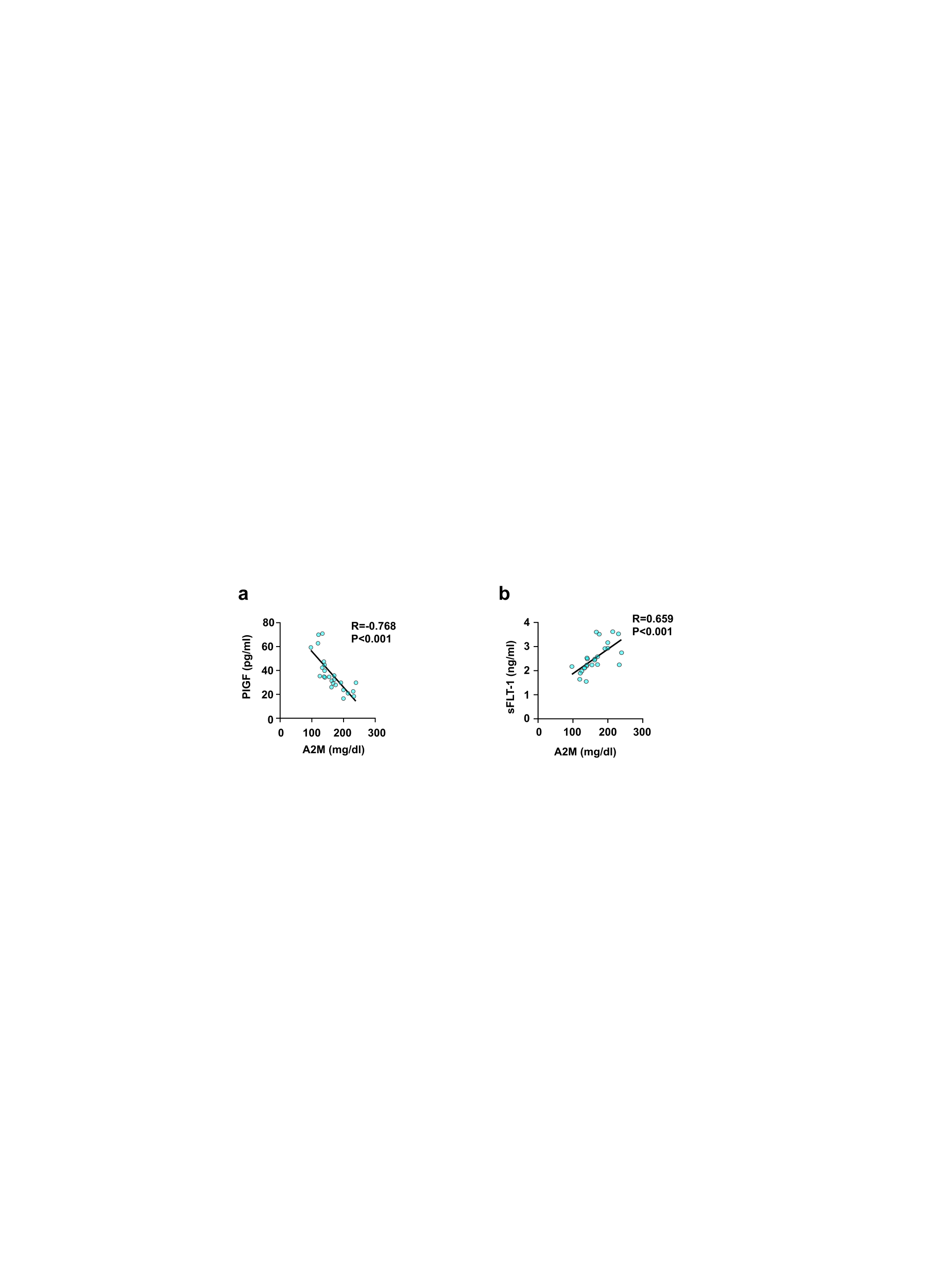
***

***Supplementary Fig. 4. Correlation between PlGF, sFLT-1 and A2M levels in maternal plasma of the preeclampsia women.***

**a:** Correlation between PlGF and A2M levels in maternal plasma of the preeclampsia women. **b:** Correlation between sFLT-1 and A2M levels in maternal plasma of the preeclampsia women.

## *Supplementary Fig. 5*

***
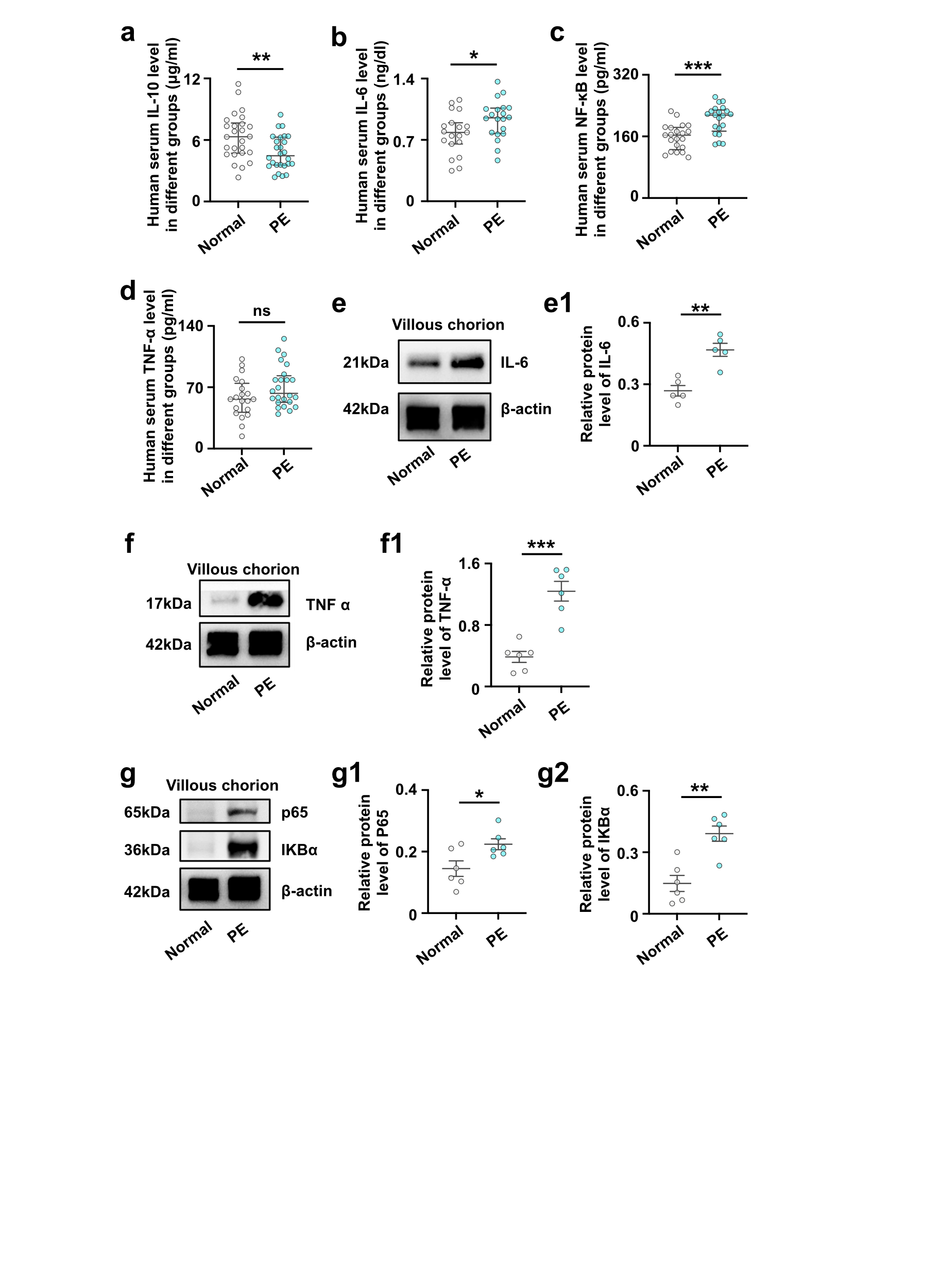
***

***Supplementary Fig. 5. Determining the serum and placental levels of human inflammatory cytokins and NF-κB***

**a-d**: Comparisons of serum levels of IL-10 (a), IL-6 (b), NF-κB (c) and TNF-α (d) between the normal and PE groups, as detected by ELISA. **e-g, e1-g1, g2**: Western blotting data showing the expression of IL-6 (e), TNF-α (f), p65 (g) and IKBα (g) in the placenta, and e1-g1, g2 are the quantitative analysis of the normal and PE groups, respectively. *P < 0.05, **P < 0.01, ***P < 0.001.

## *Supplementary Fig. 6*


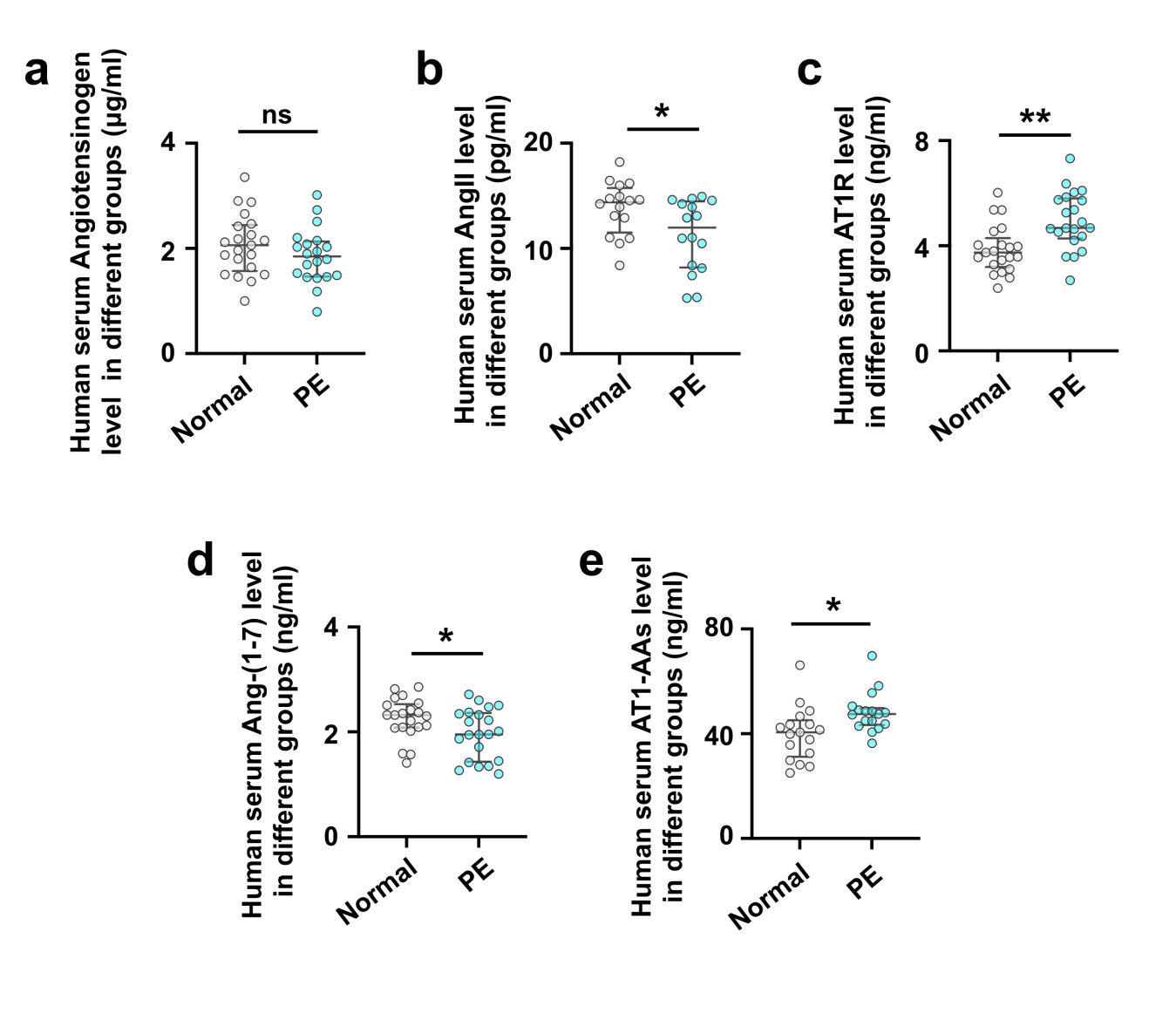


***Supplementary Fig. 6. Determining key components of the RAAS system in human serum.***

**a-e**: ELISA data showing angiotensinogen (a), Ang II (b), AT1R (c), Ang-(1-7) (d) and AT1-AA (e) levels in human maternal serum in the normal and PE groups. *P < 0.05, **P < 0.01.

## *Supplementary Fig. 7*


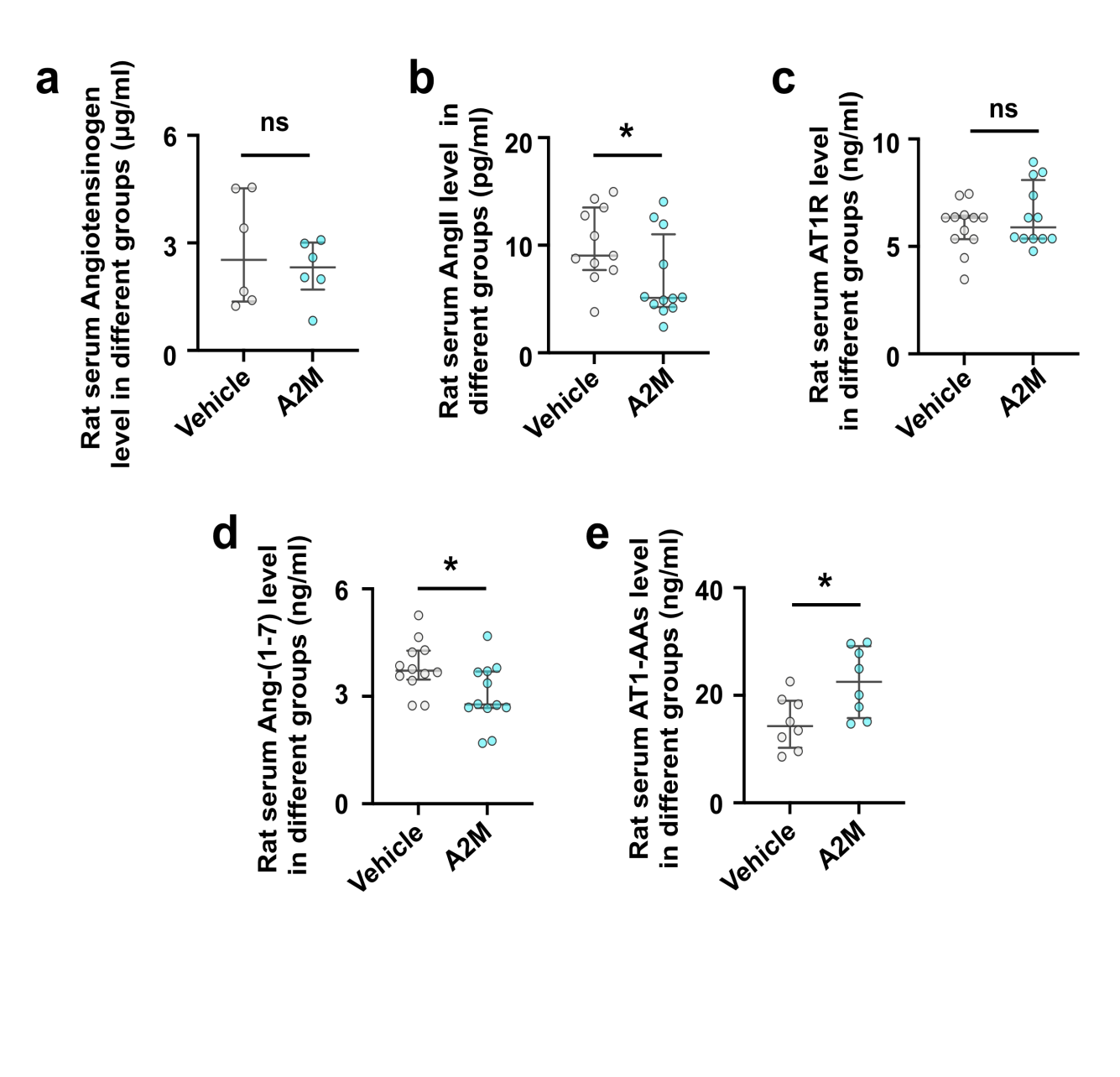


***Supplementary Fig. 7. Determining key components of the RAAS system in rat serum in the presence of high levels of A2M.***

**a-e**: ELISA data showing angiotensinogen (a), Ang II (b), AT1R (c), Ang-(1-7) (d) and AT1-AA (e) levels in GD19.5 rat serum in the control and A2M-overexpression groups. *P < 0.05.

## *Supplementary Fig. 8*


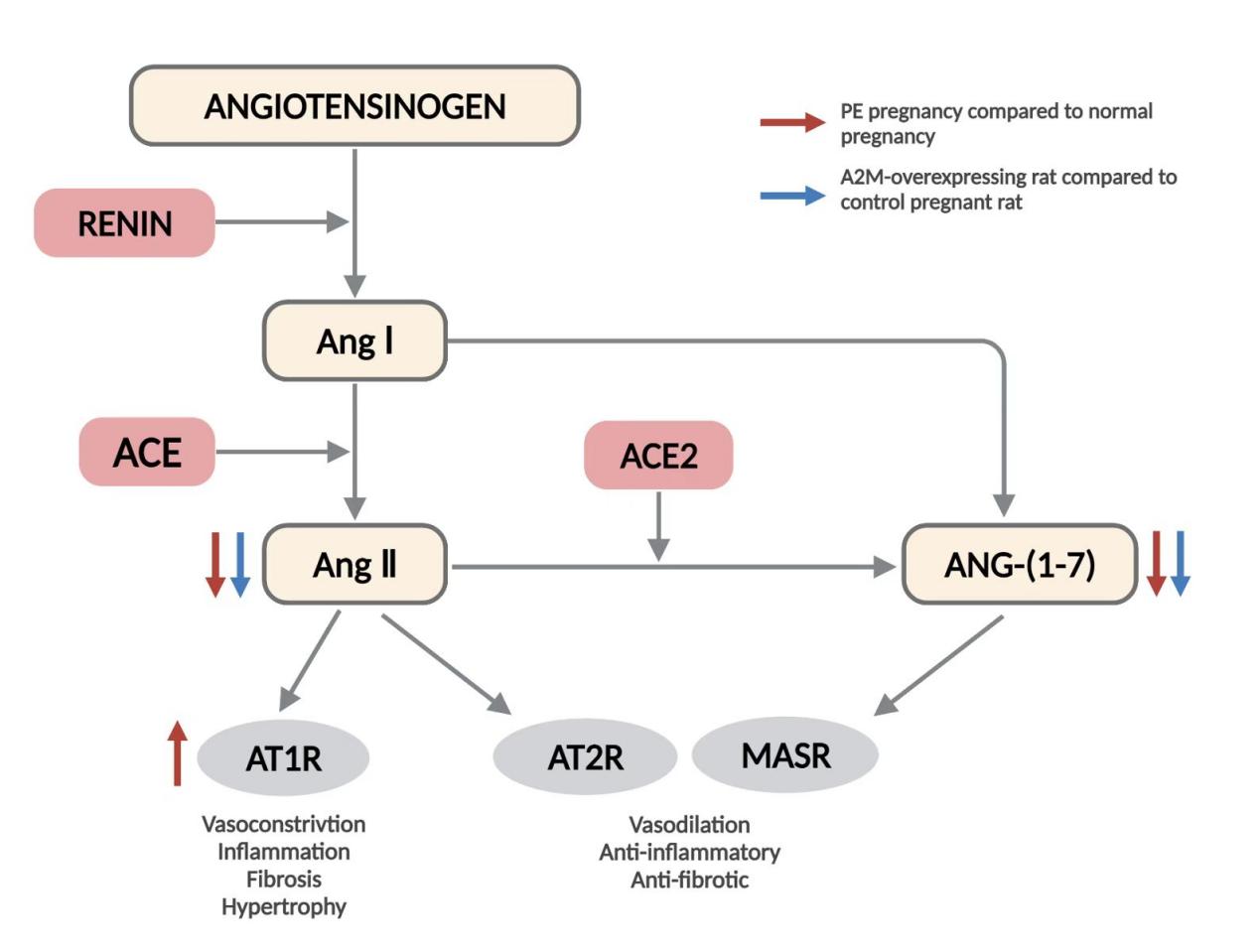


***Supplementary Fig. 8. Schematic illustration of the changes in key components of the RAAS system in the presence of high A2M levels.***
