# Supplementary material for: Alpha-2-macroglobulin is involved in the occurrence of early-onset pre-eclampsia via its negative impact on uterine spiral artery remodeling and placental angiogenesis
Source: BMC Med. 2023 Mar 9;21:90. doi: 10.1186/s12916-023-02807-9 (PMC9999529; doi:10.1186/s12916-023-02807-9)

Blots for figure. 1

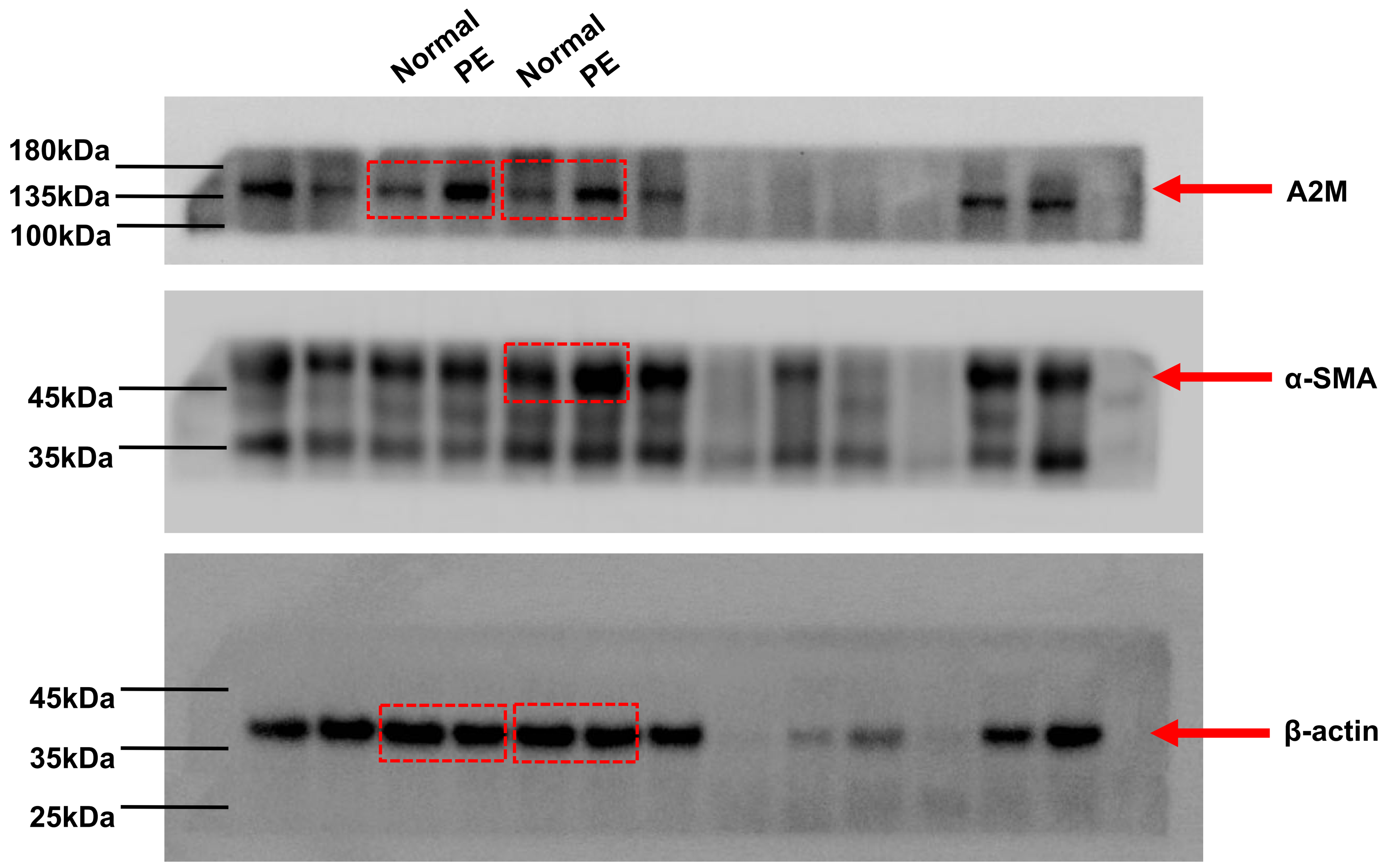

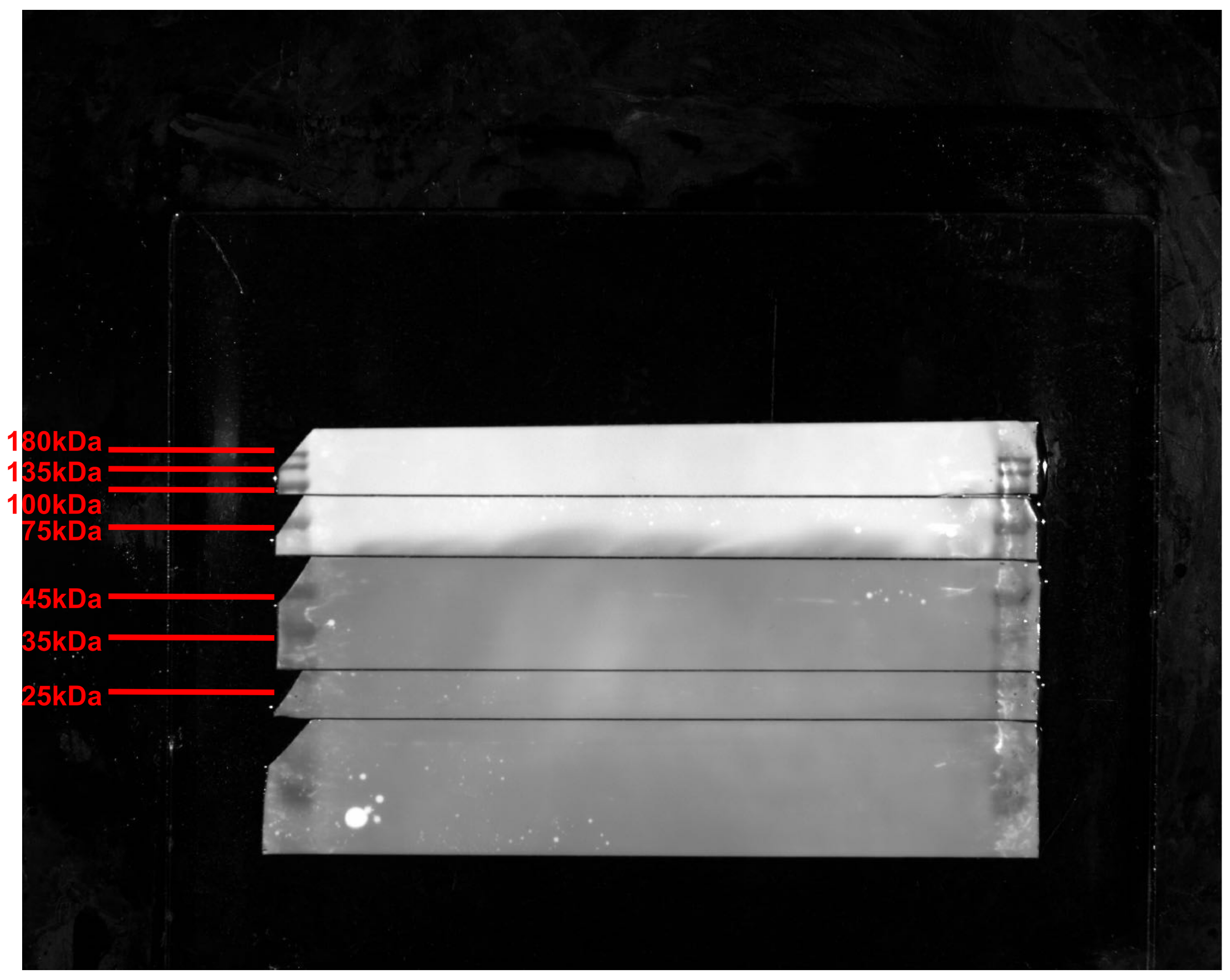

Blots for figure. 2

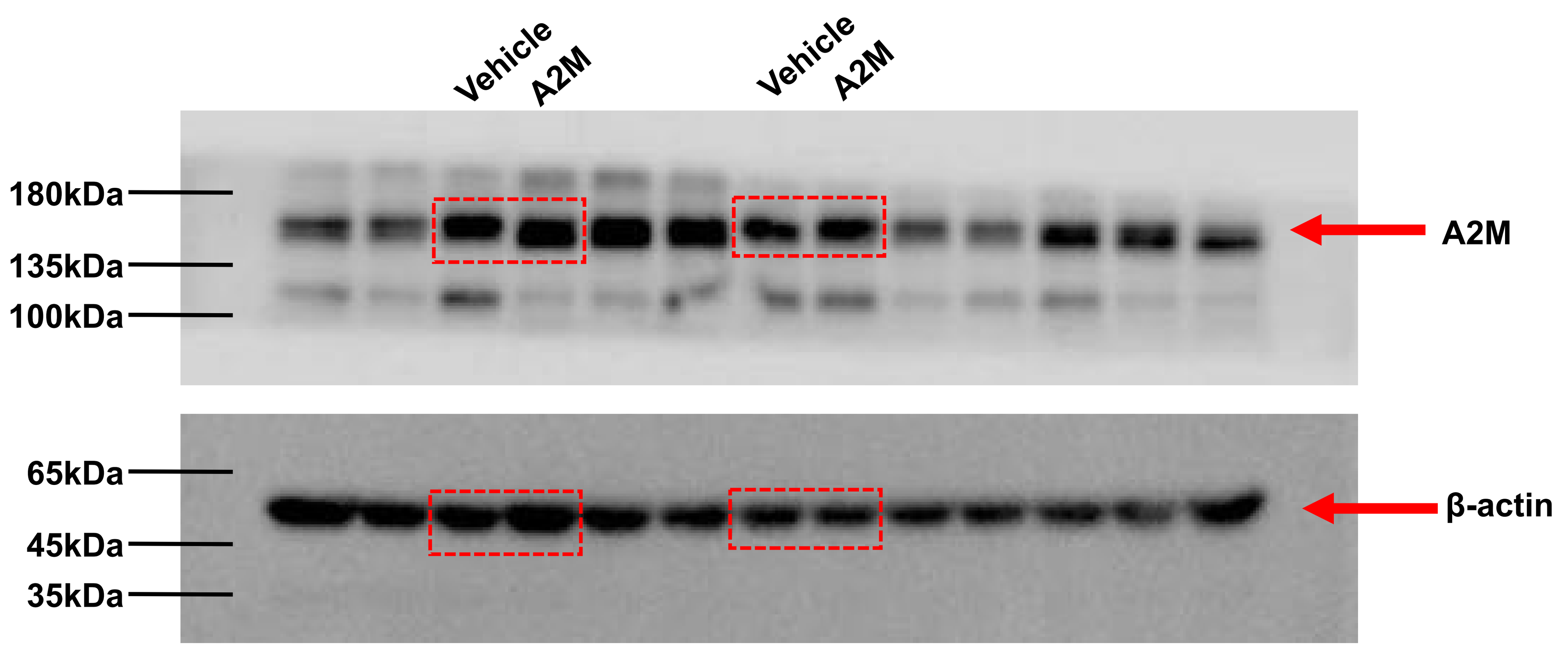

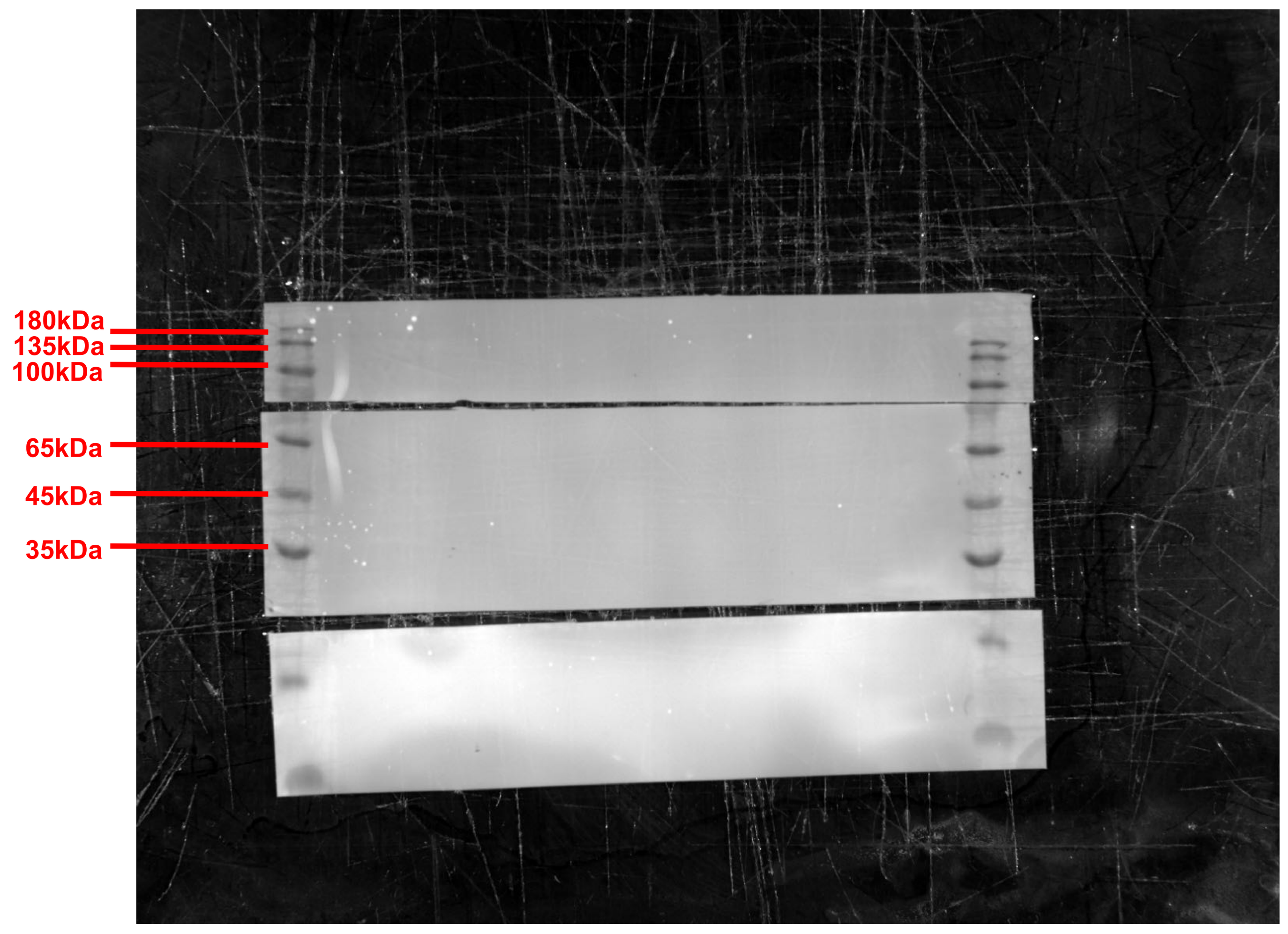

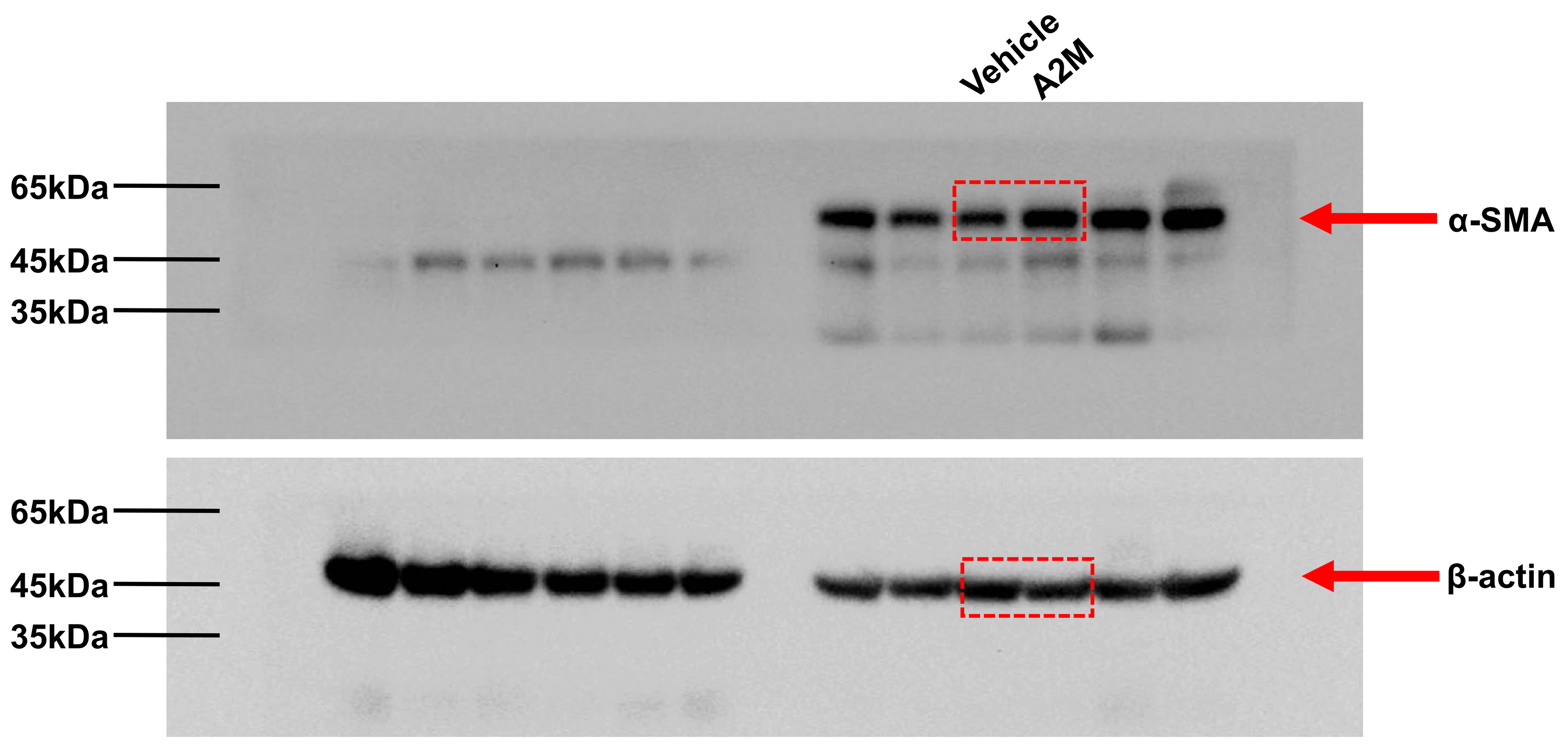

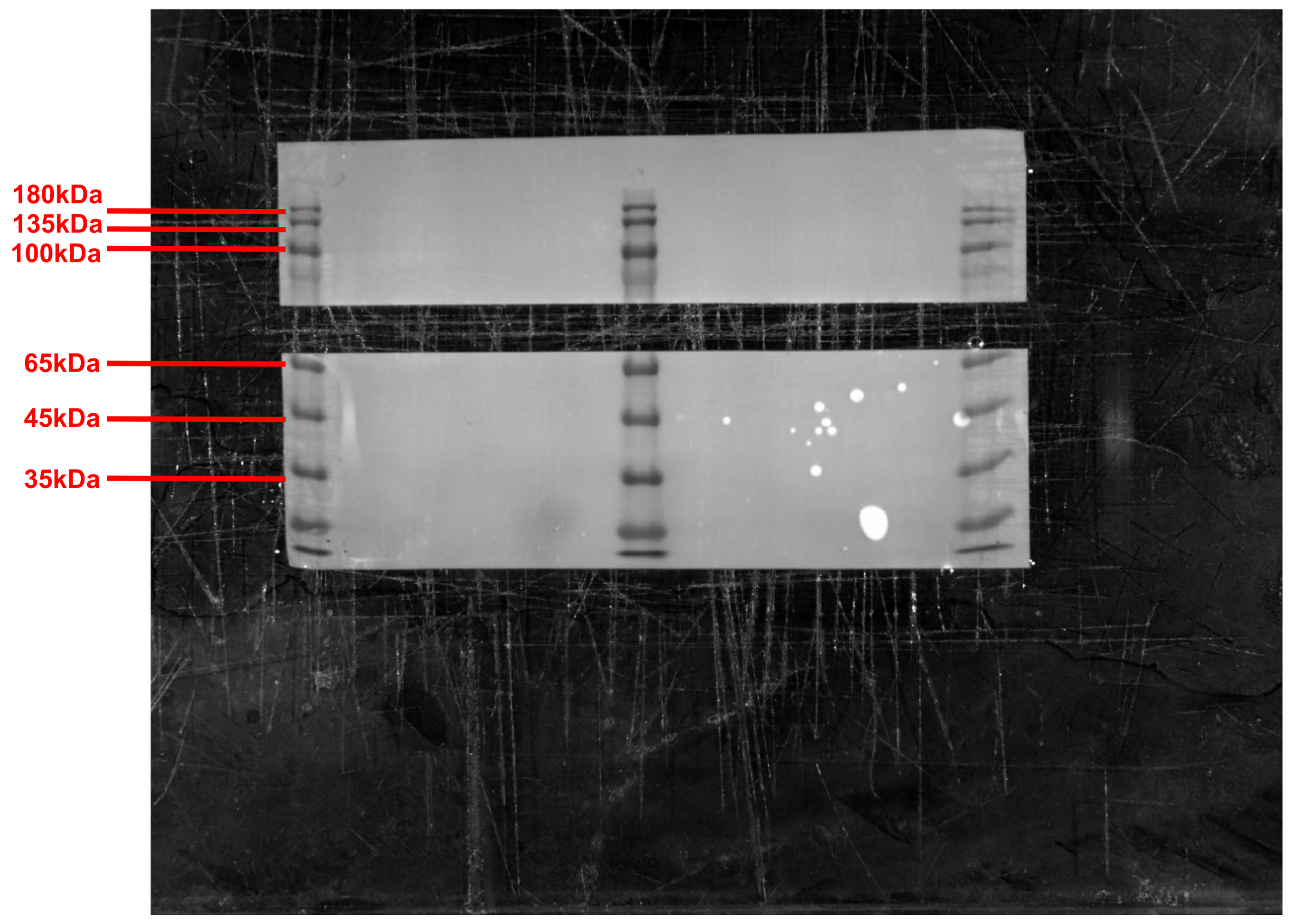

Blots for figure. 3

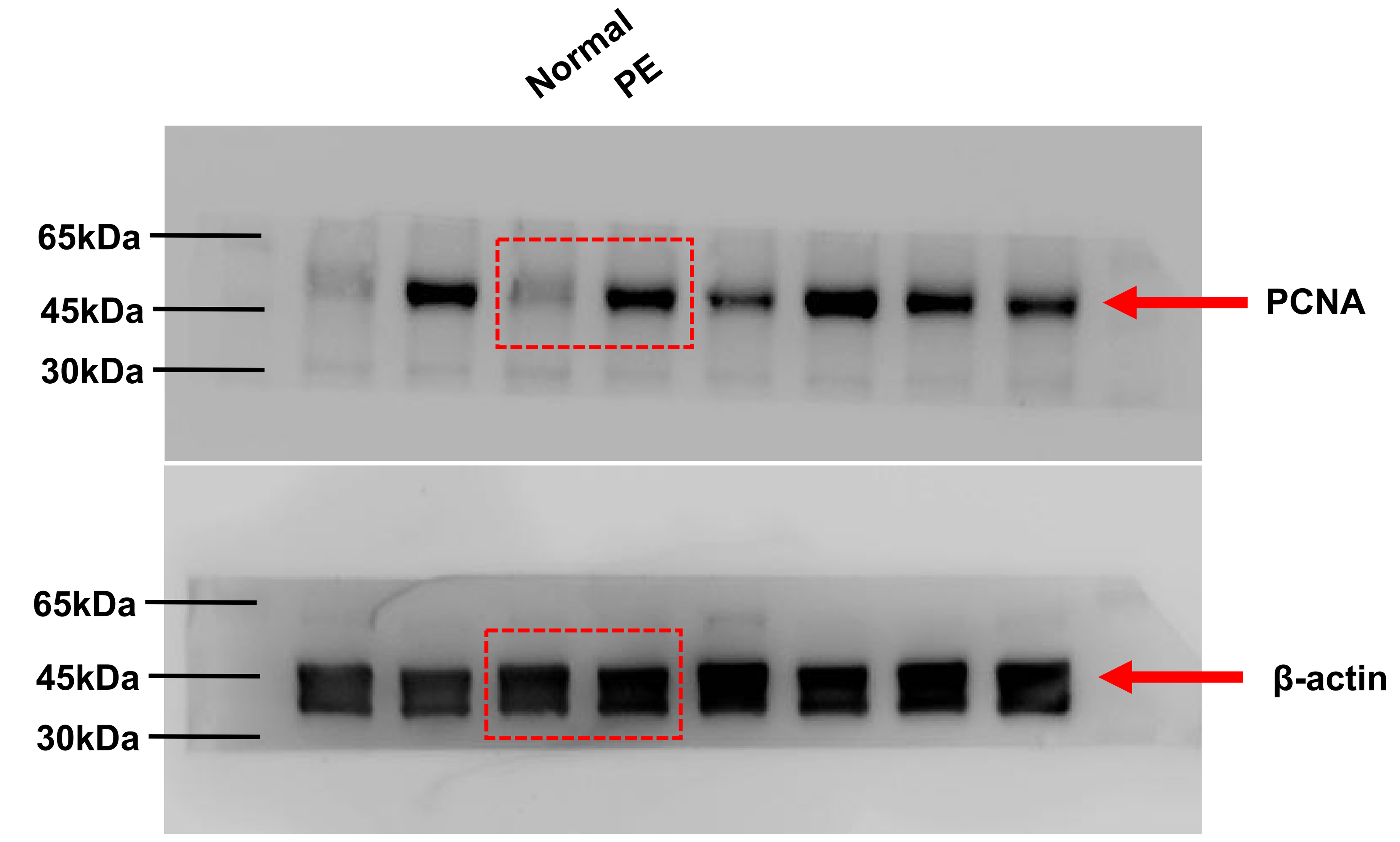

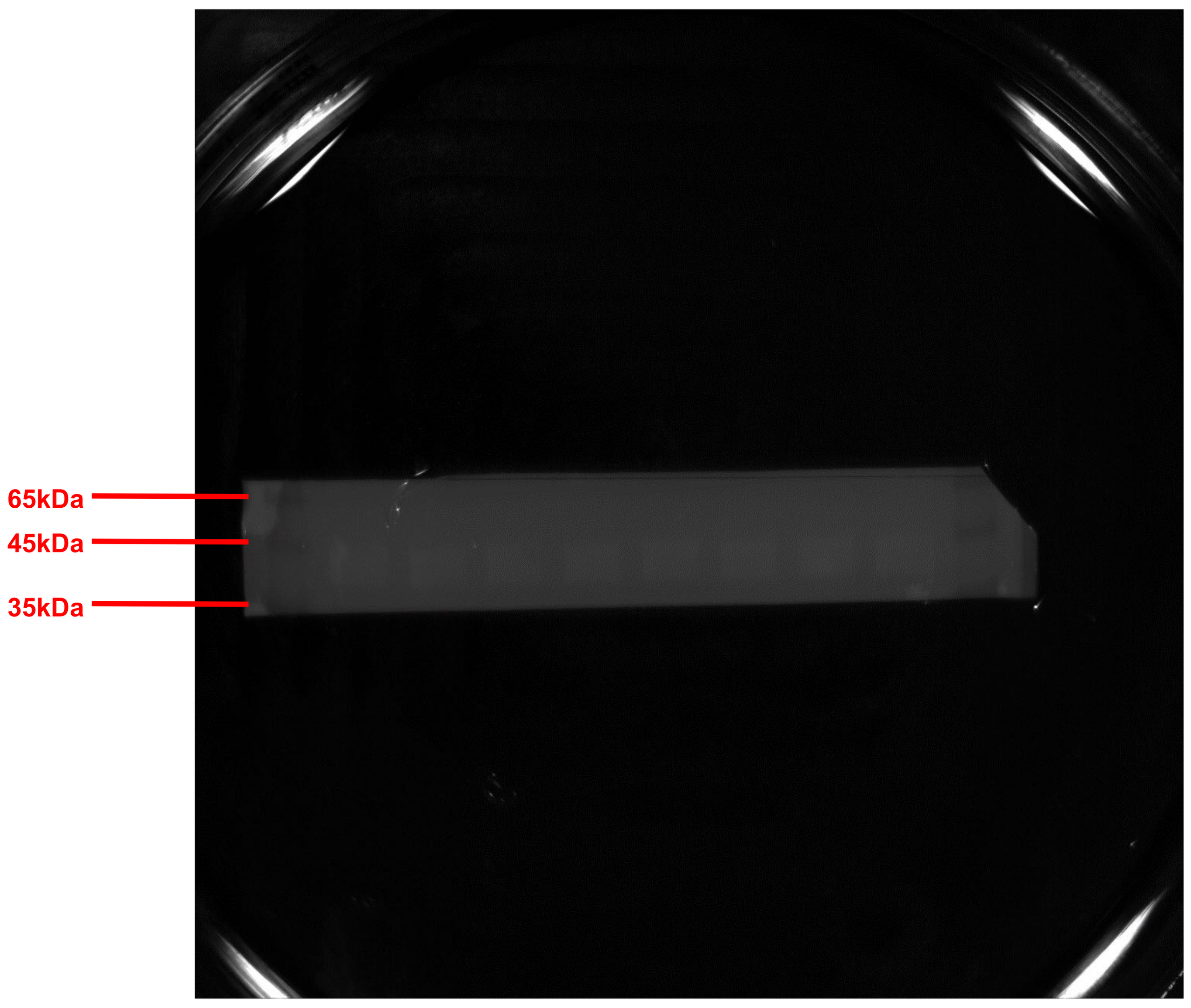

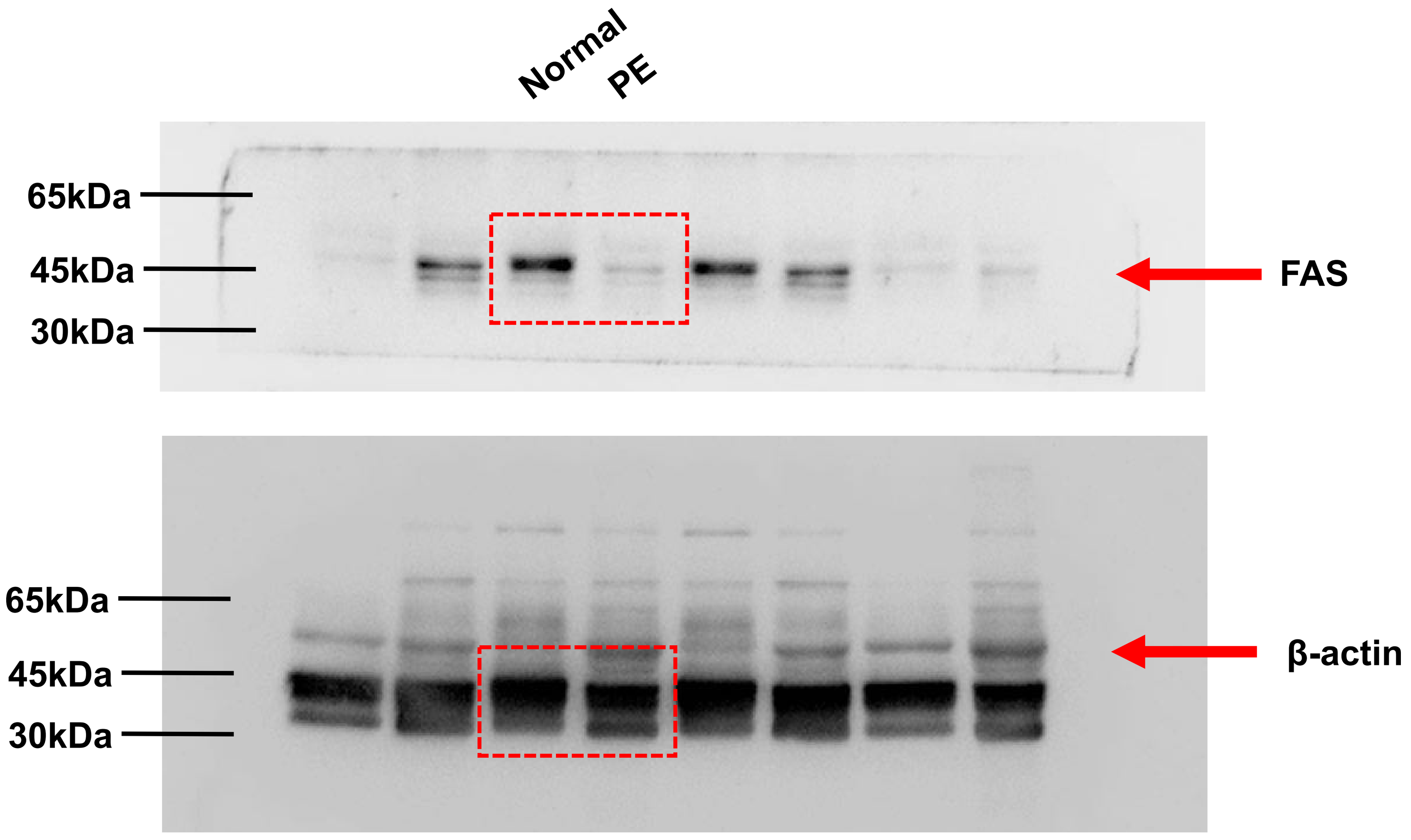

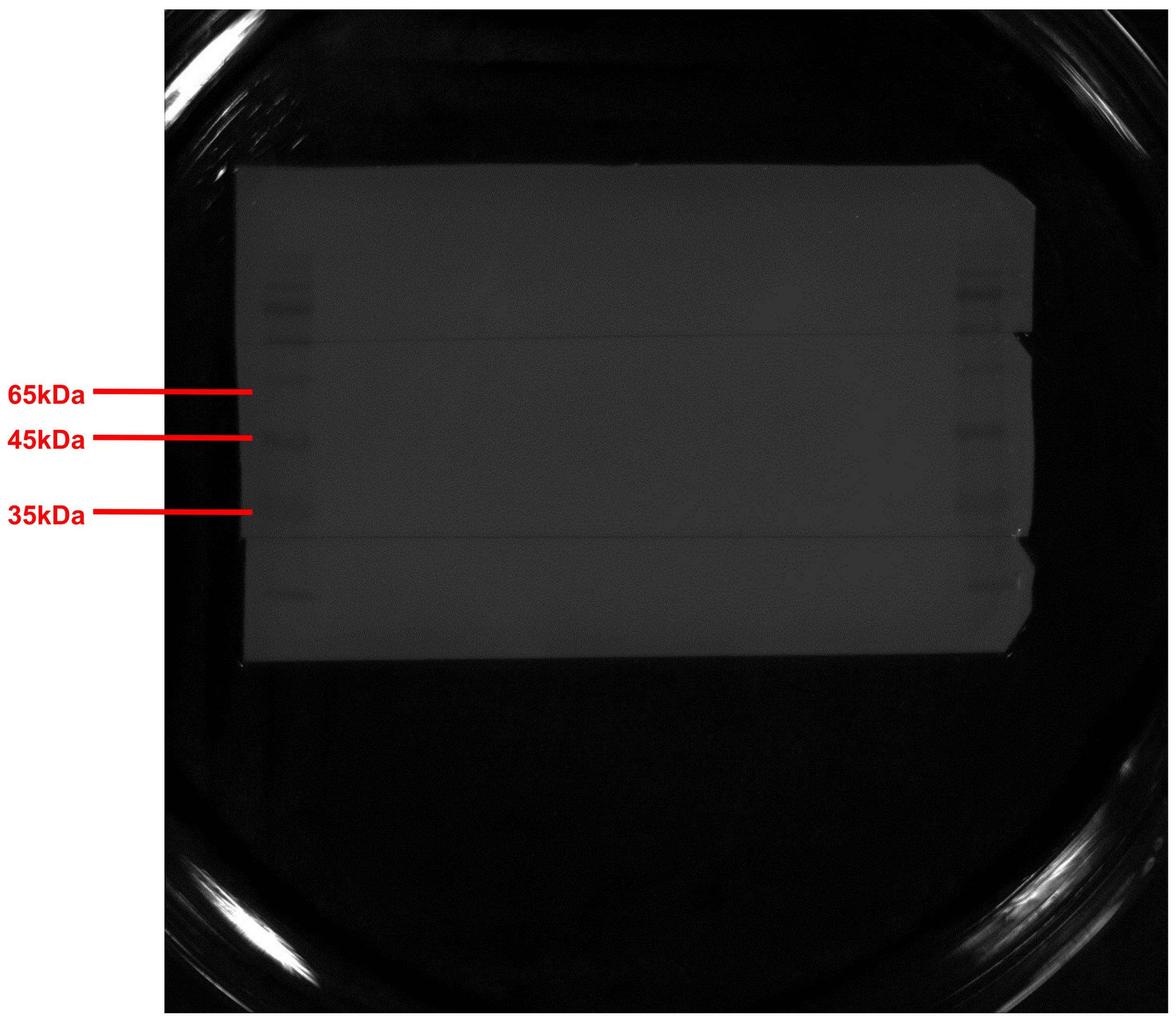

Blots for figure. 4

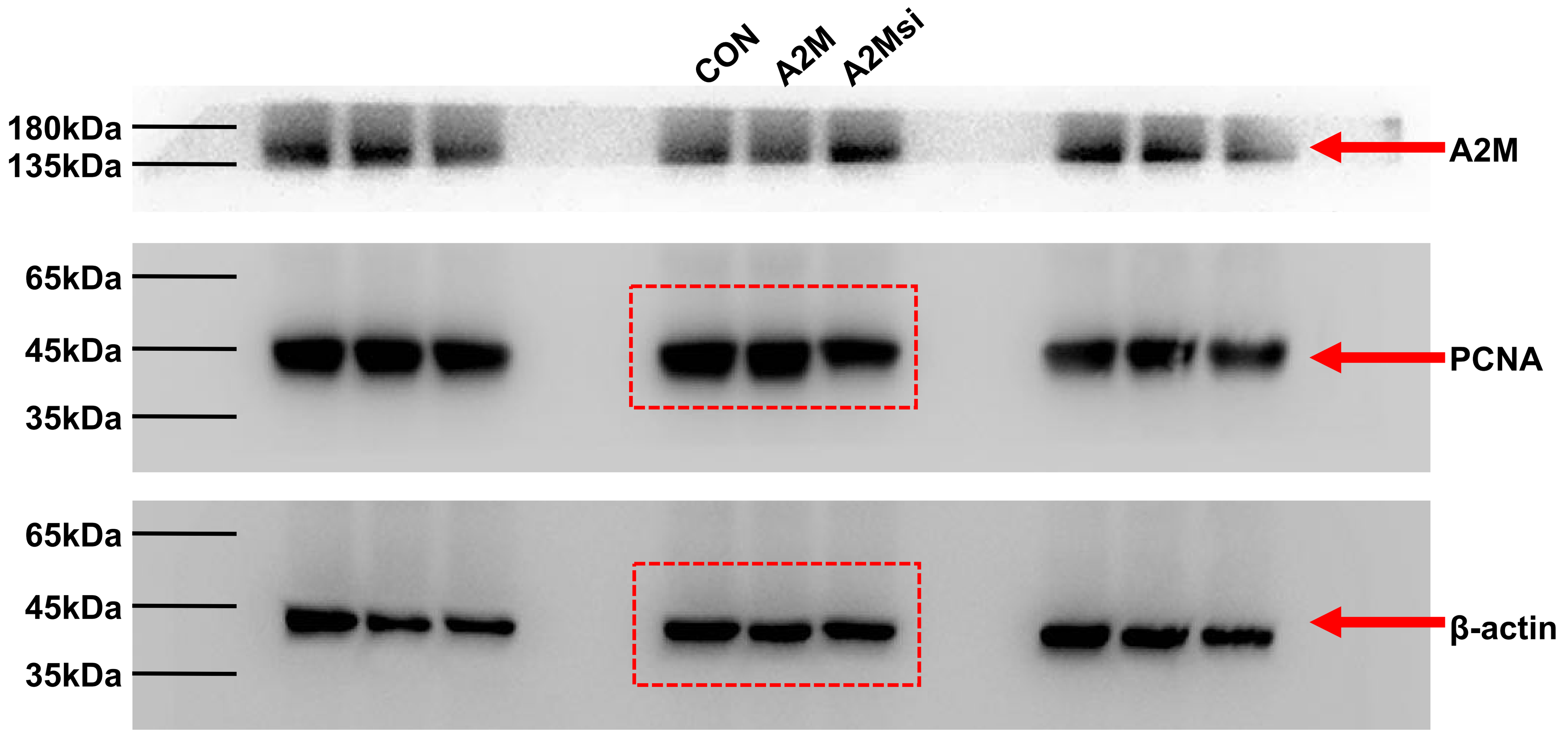

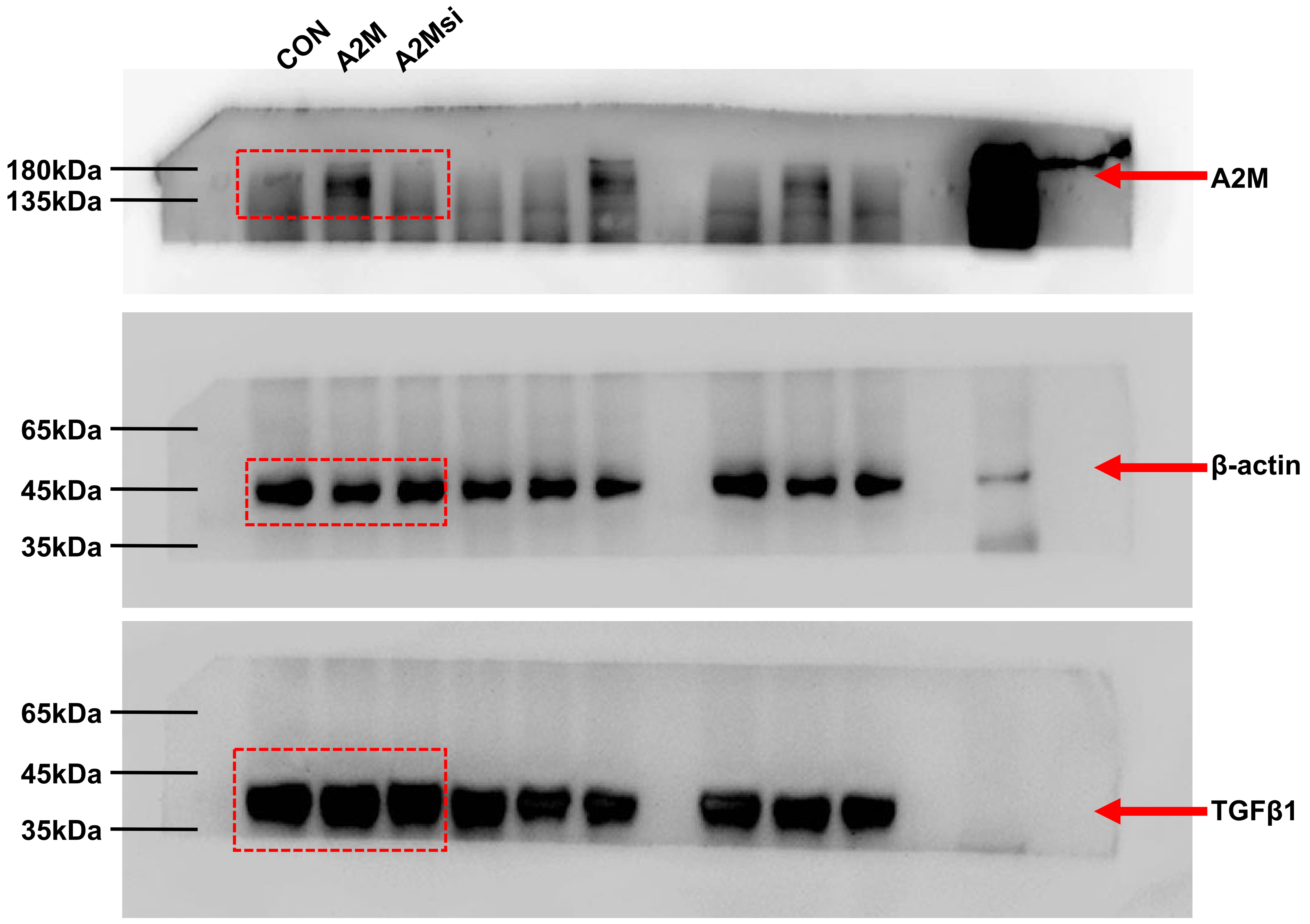

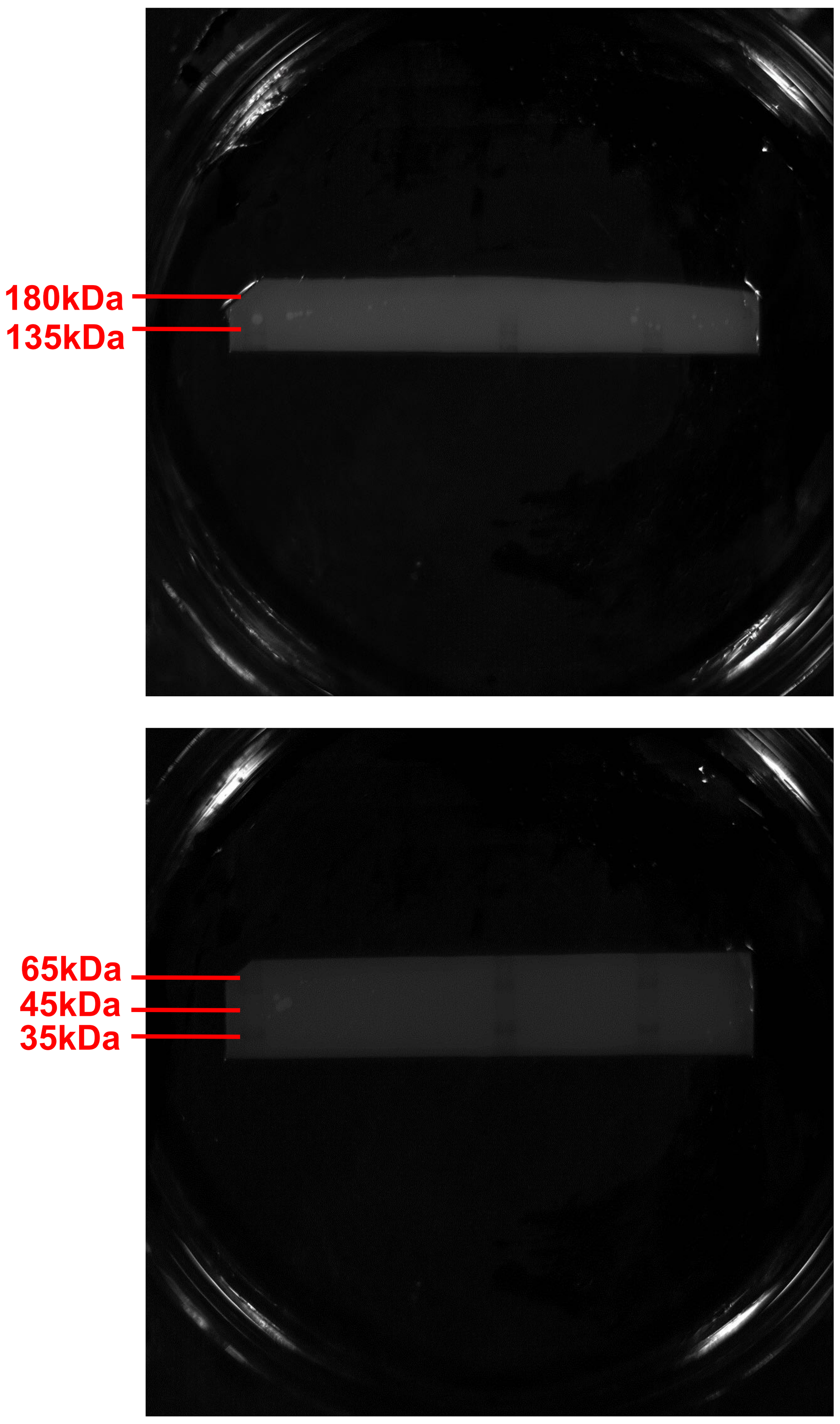

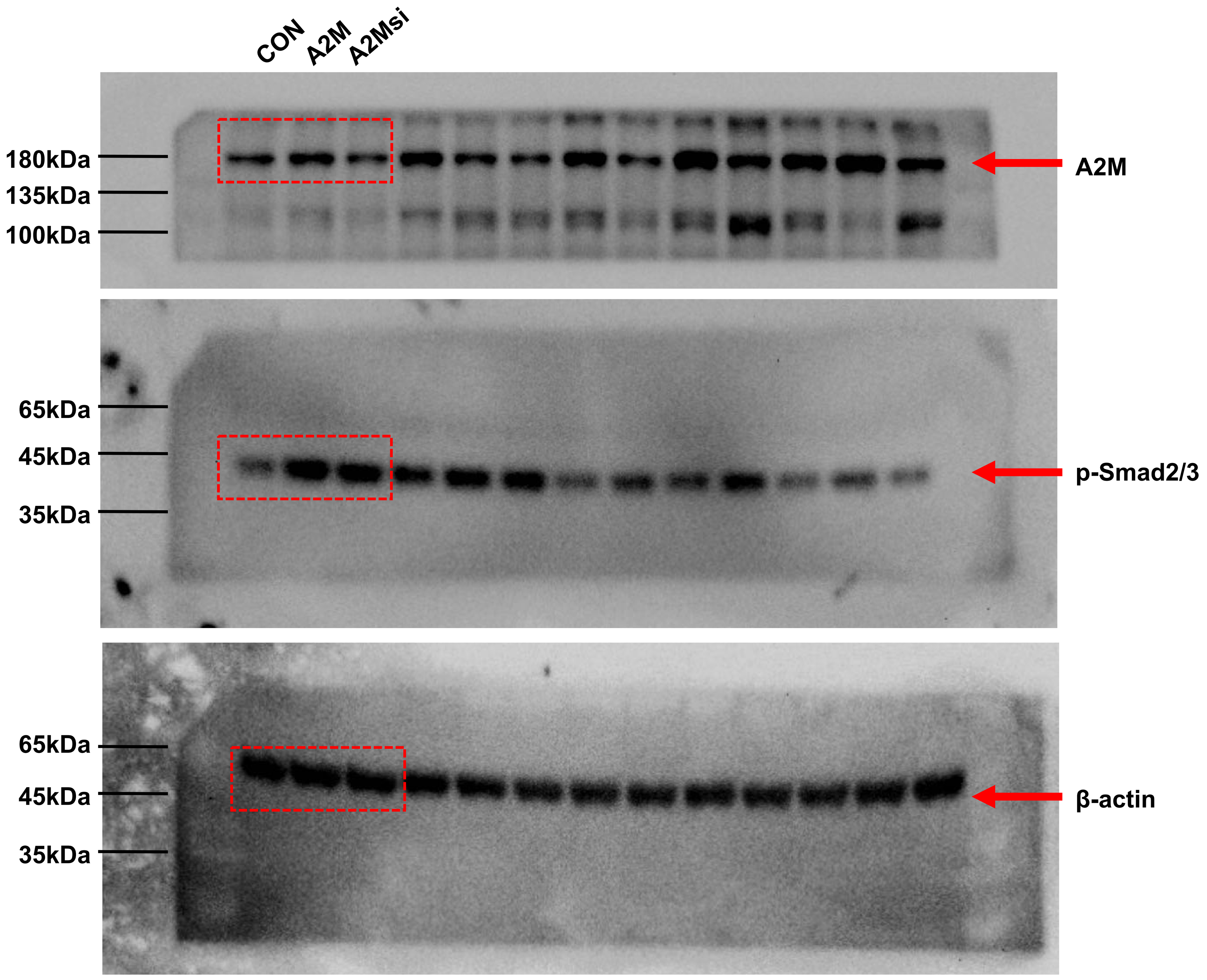

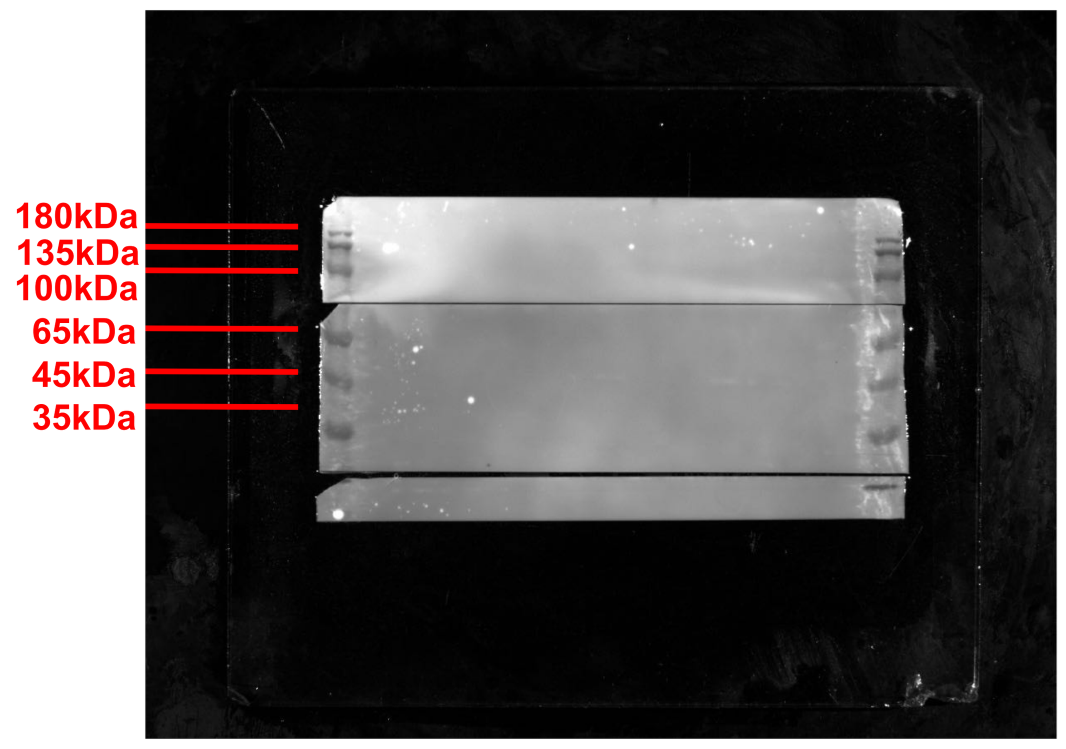

Blots for figure. 4

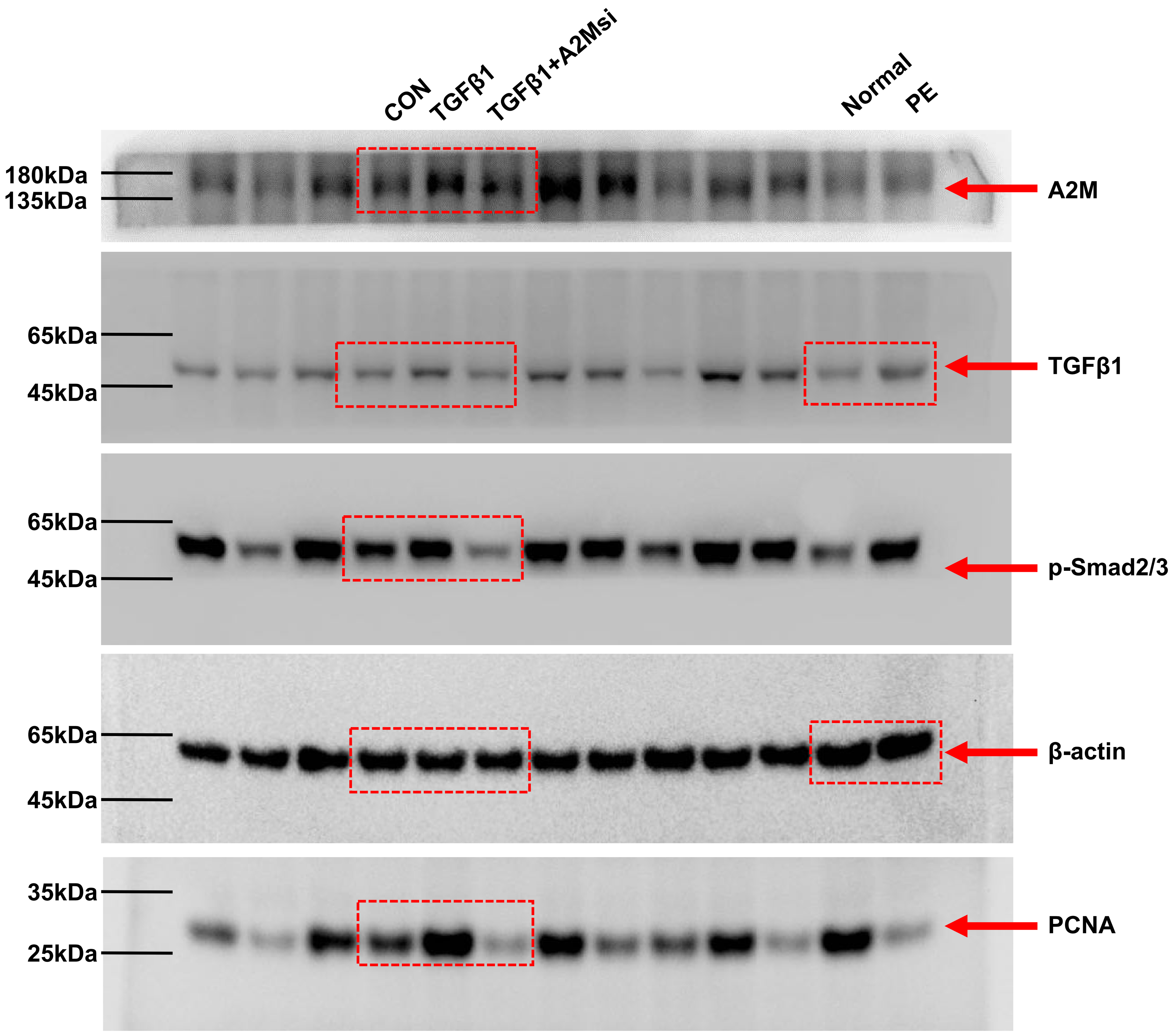

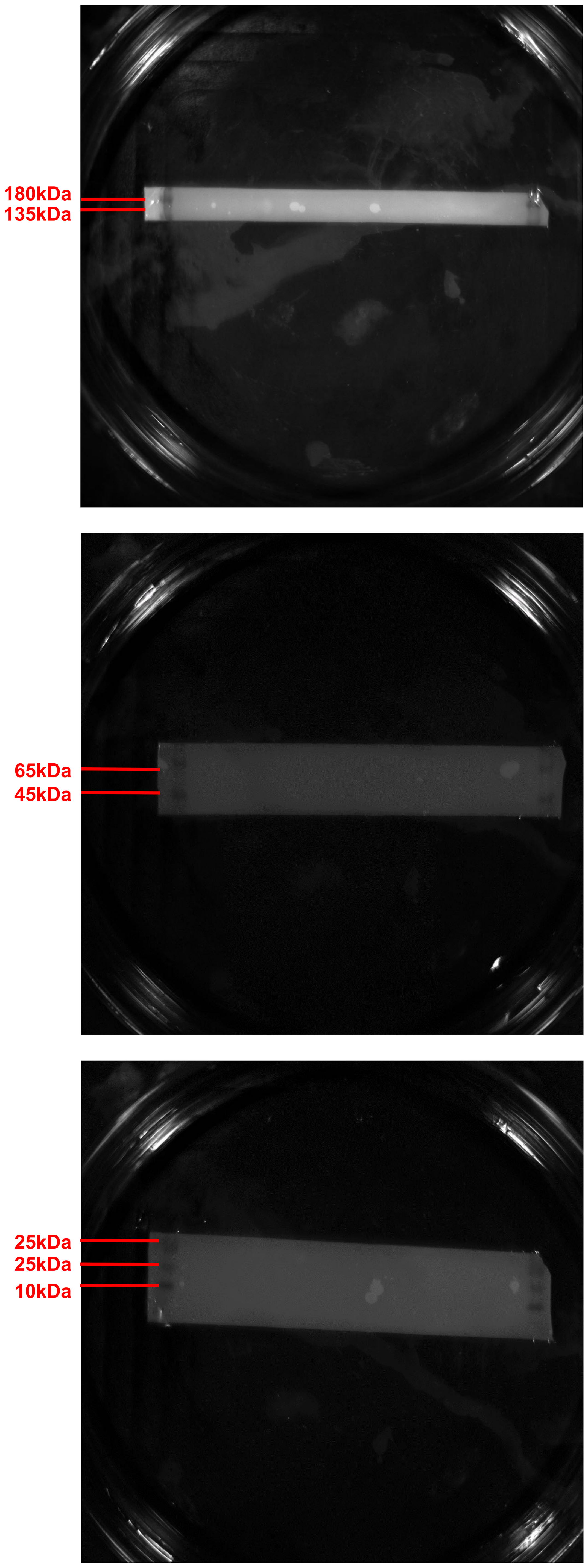

Blots for figure. 5

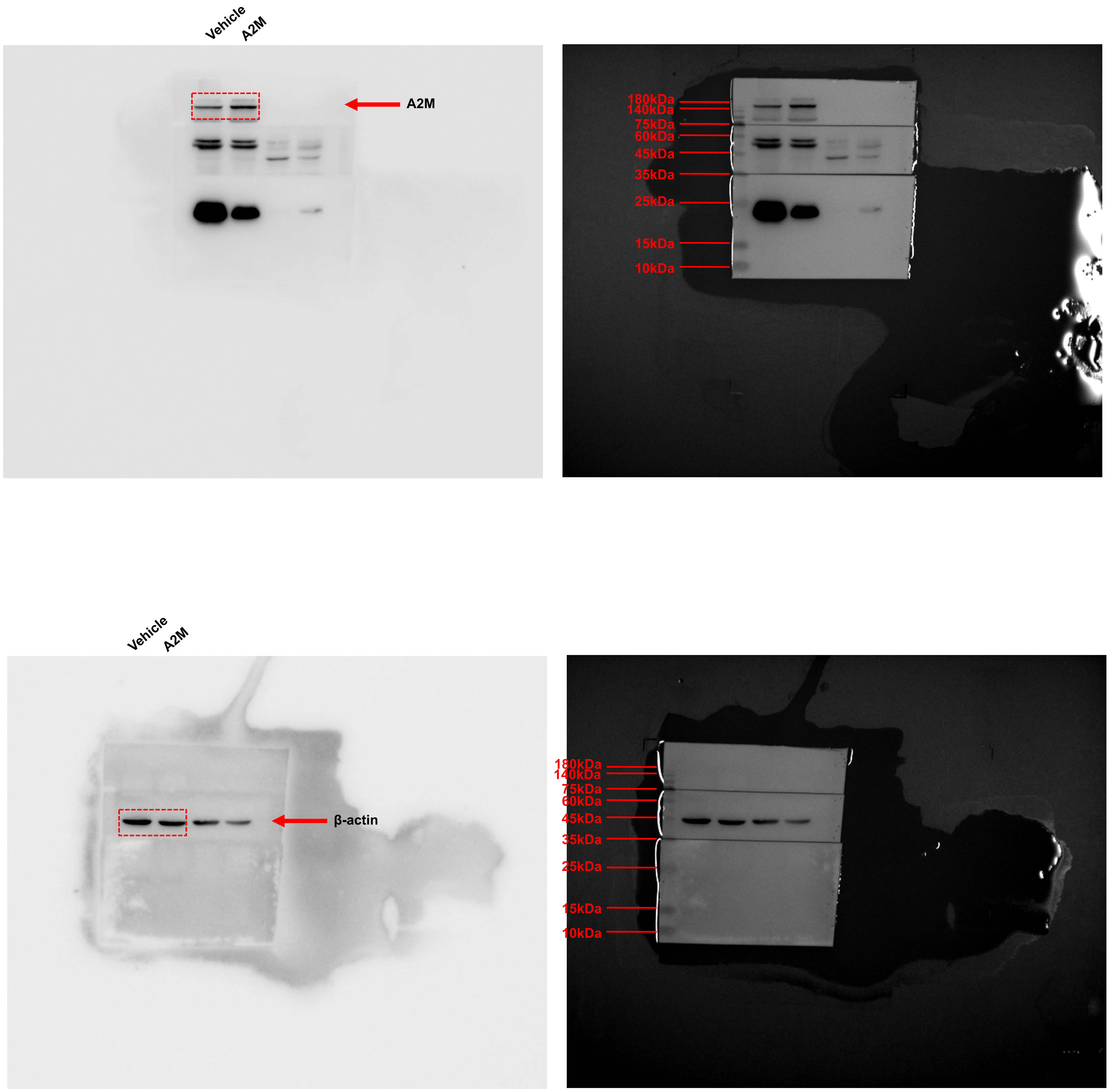

Blots for figure. 6

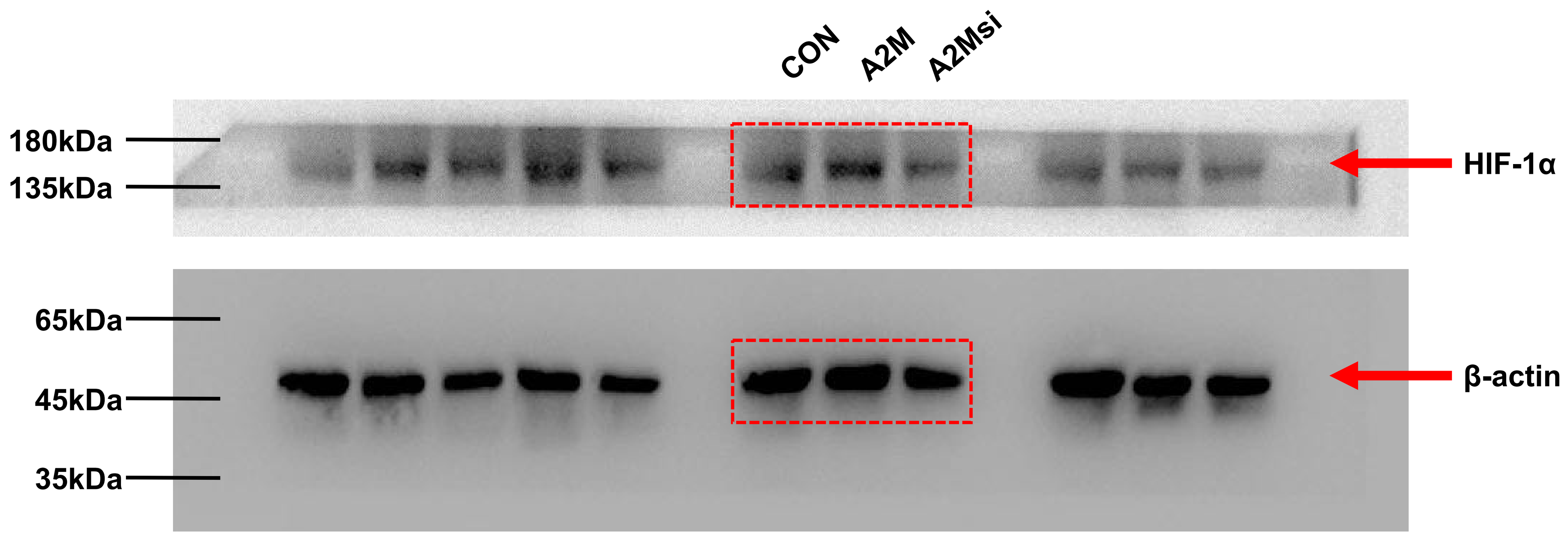

Blots for Supplementary Fig. 2

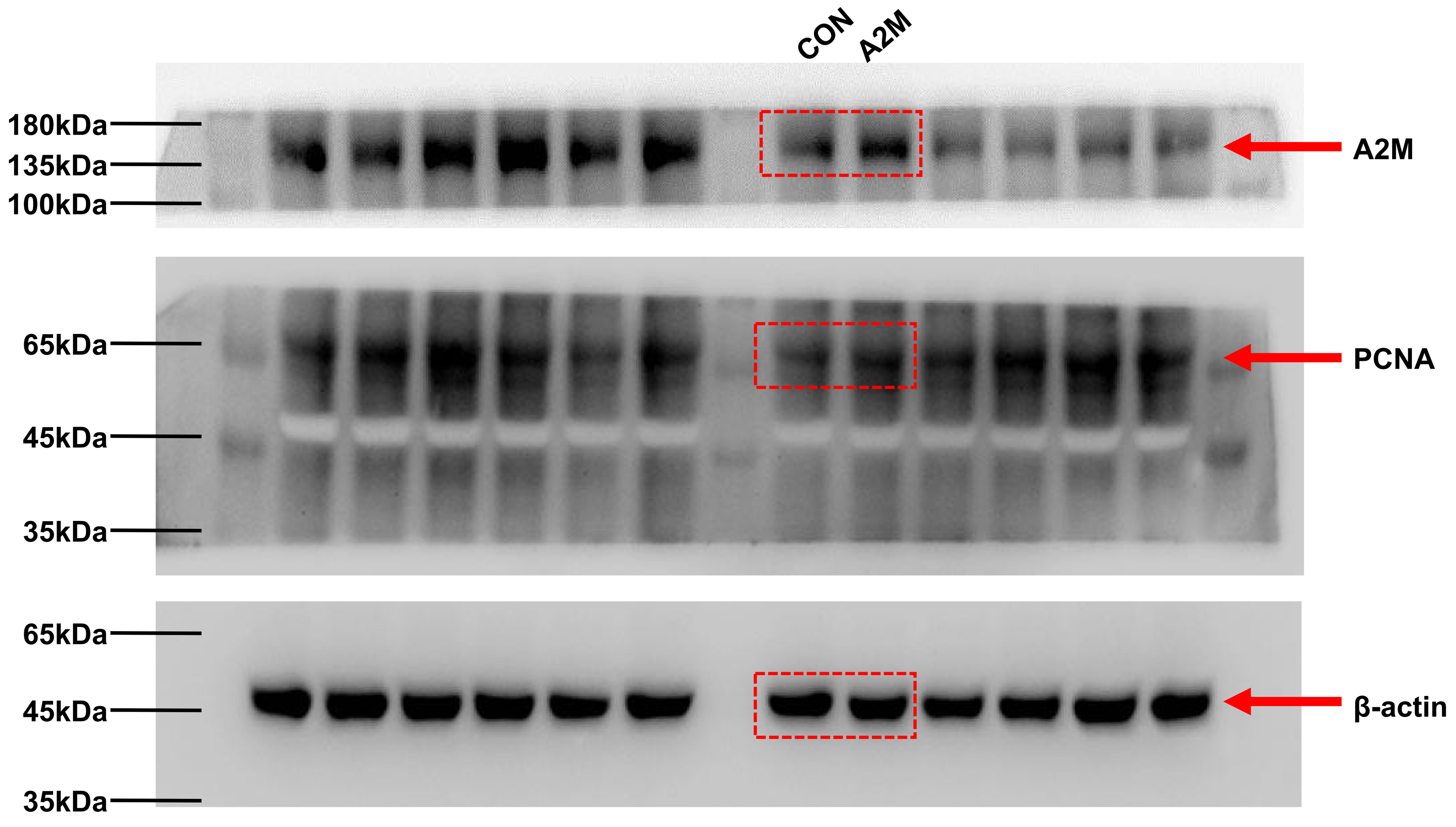

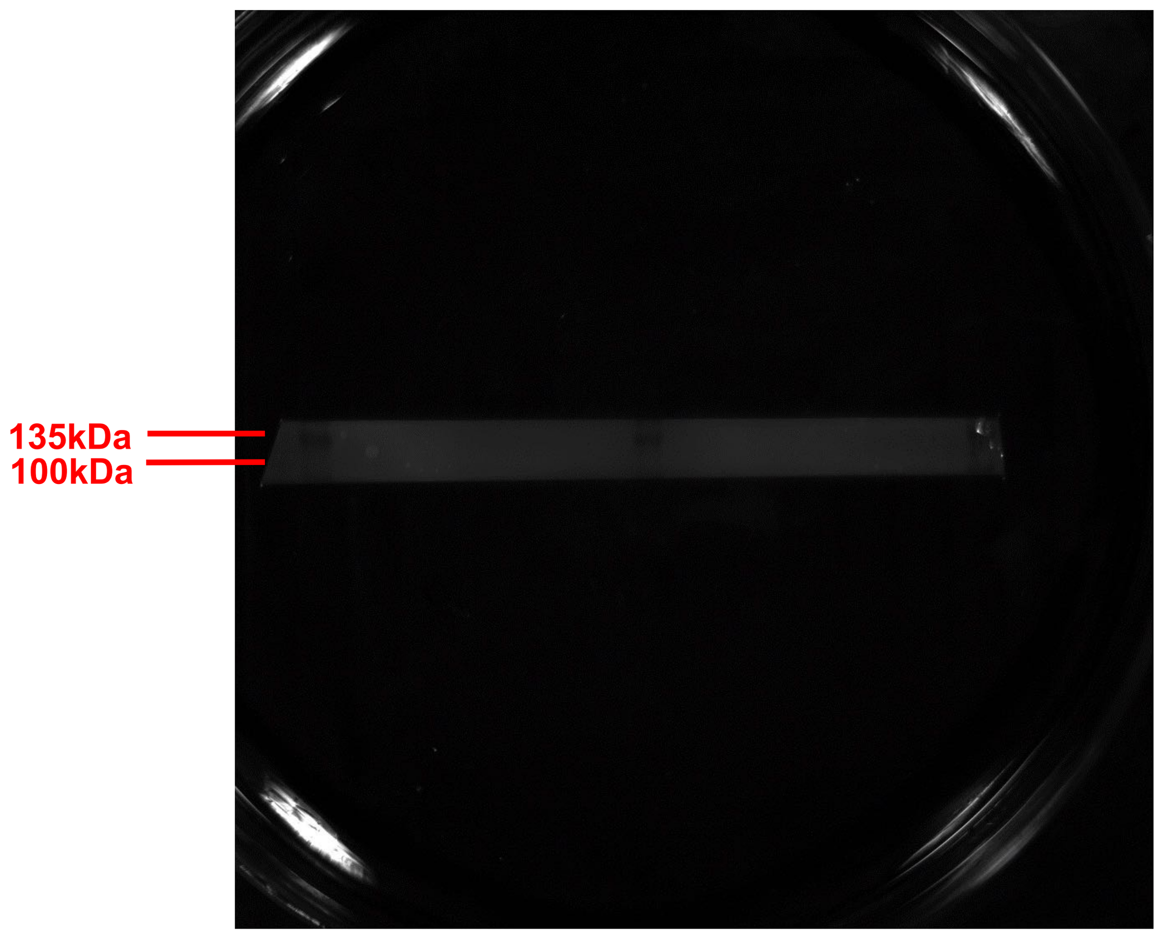

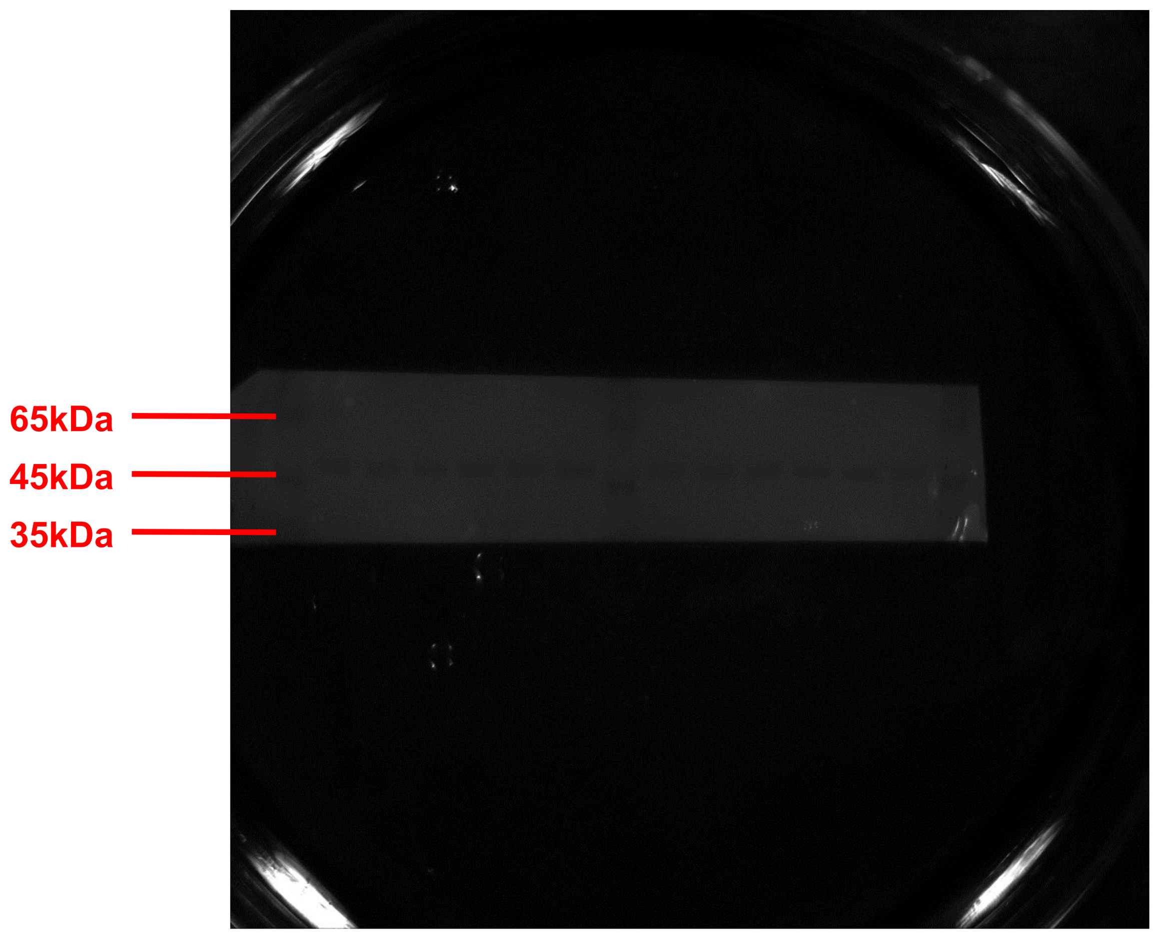

Blots for figure. Supplementary Fig. 6

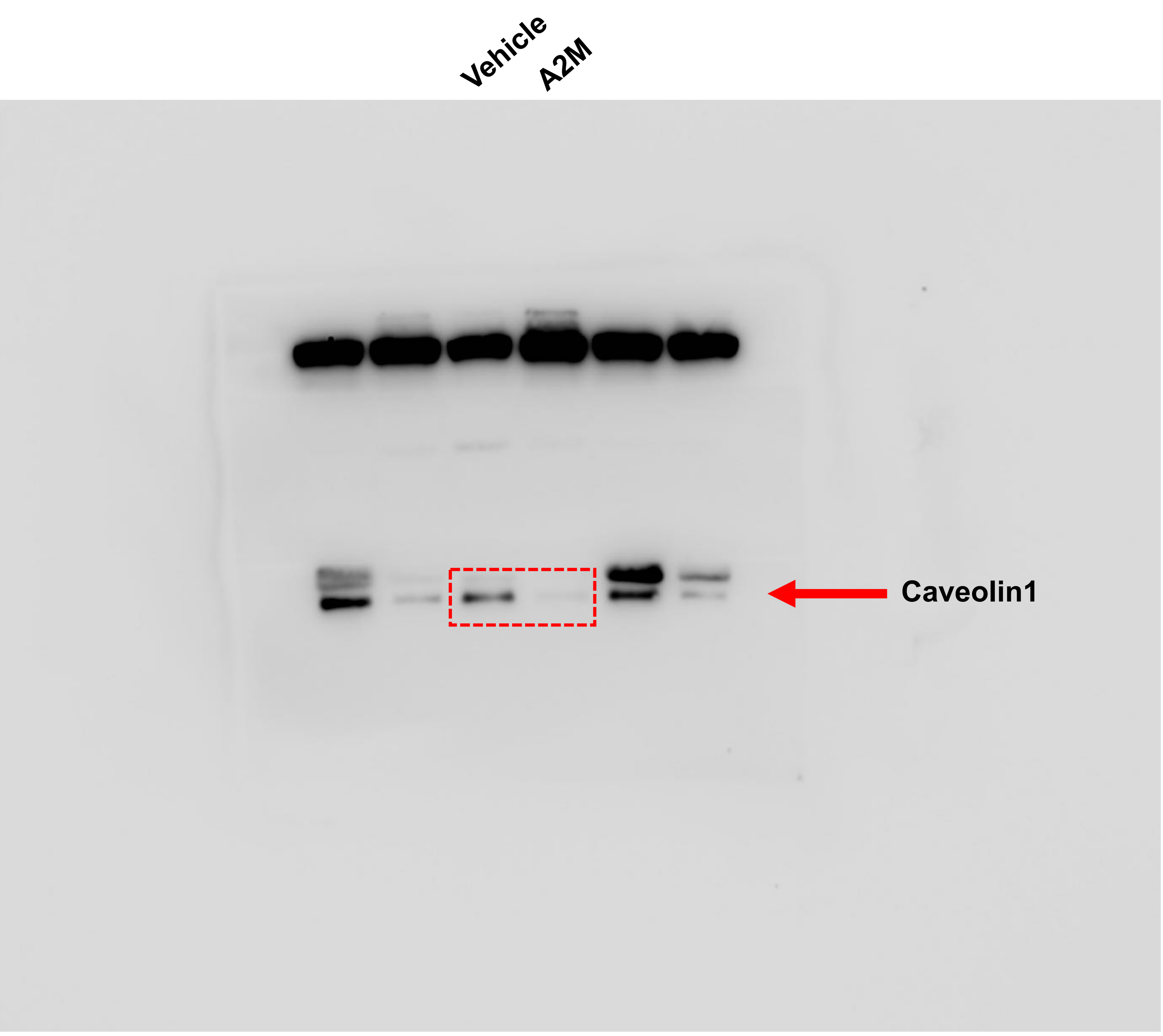

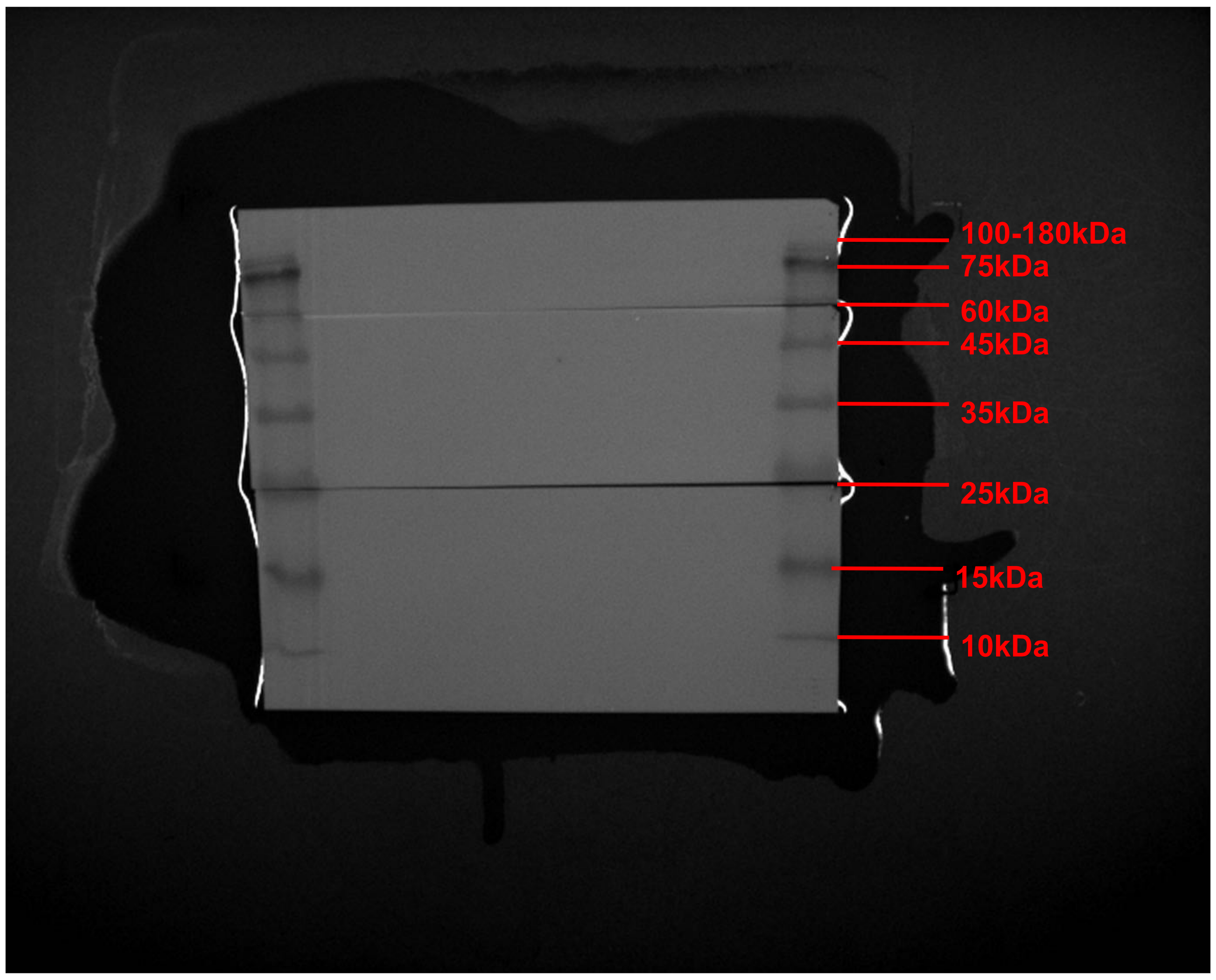

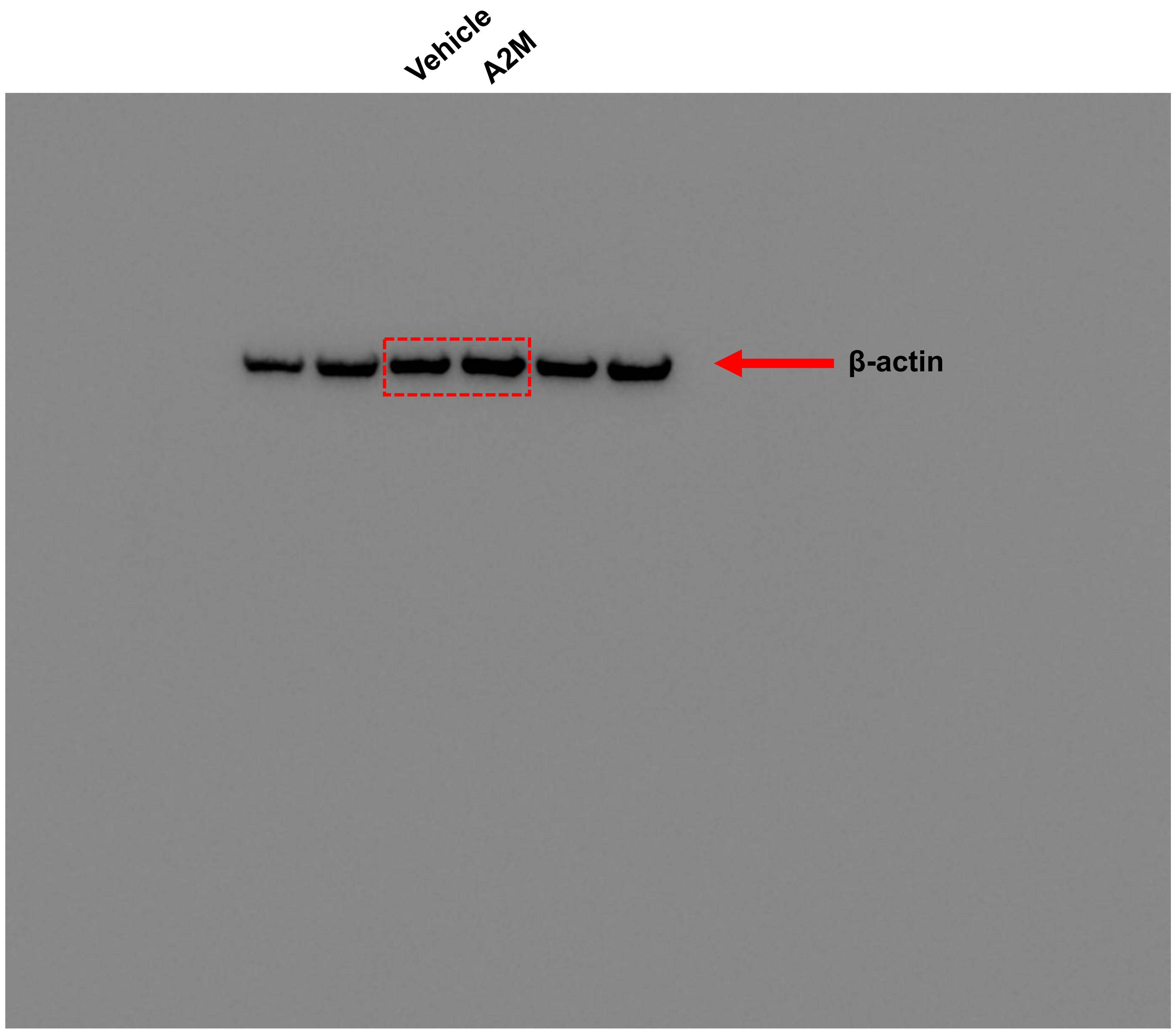

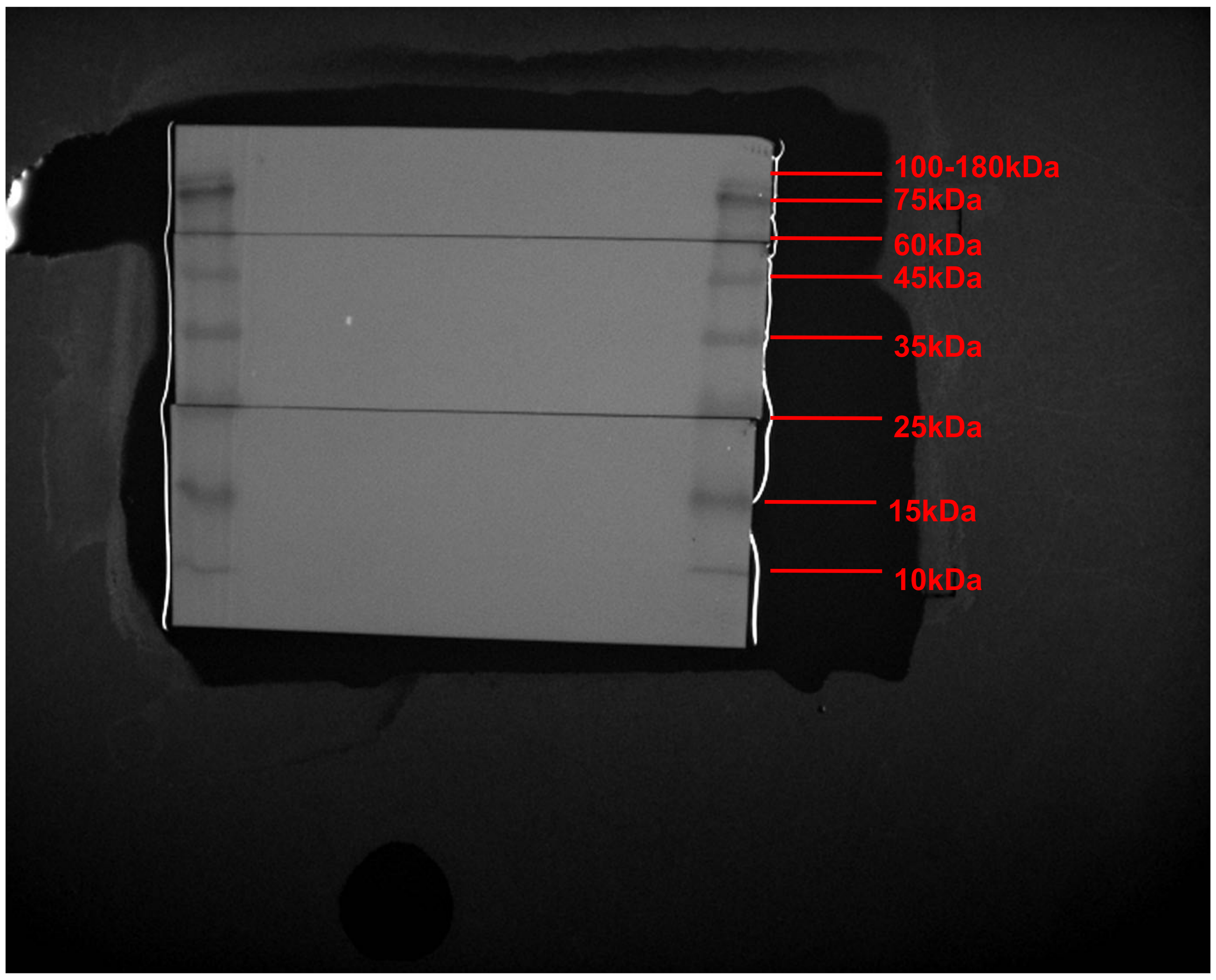

Blots for figure. Supplementary Fig. 6

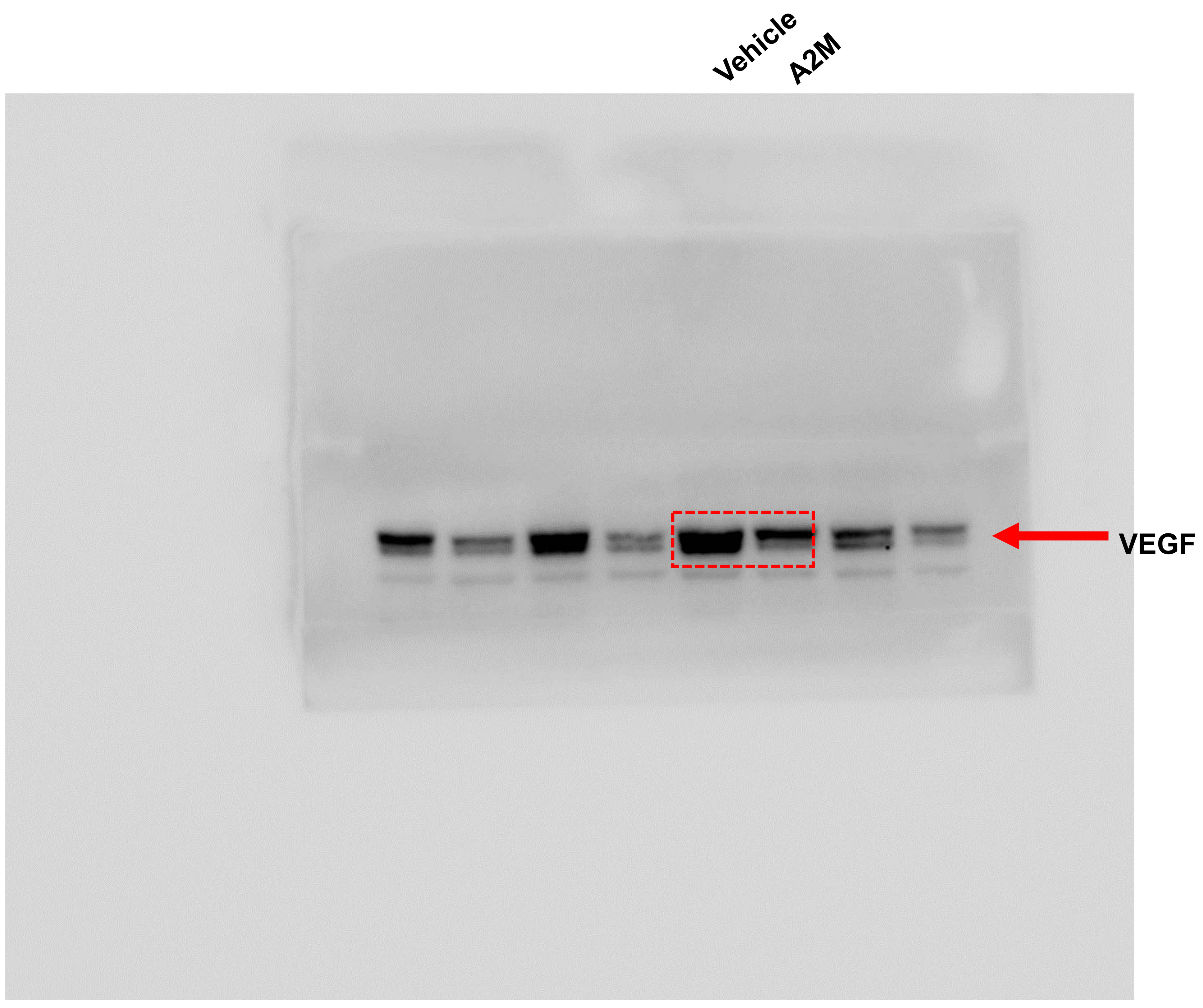

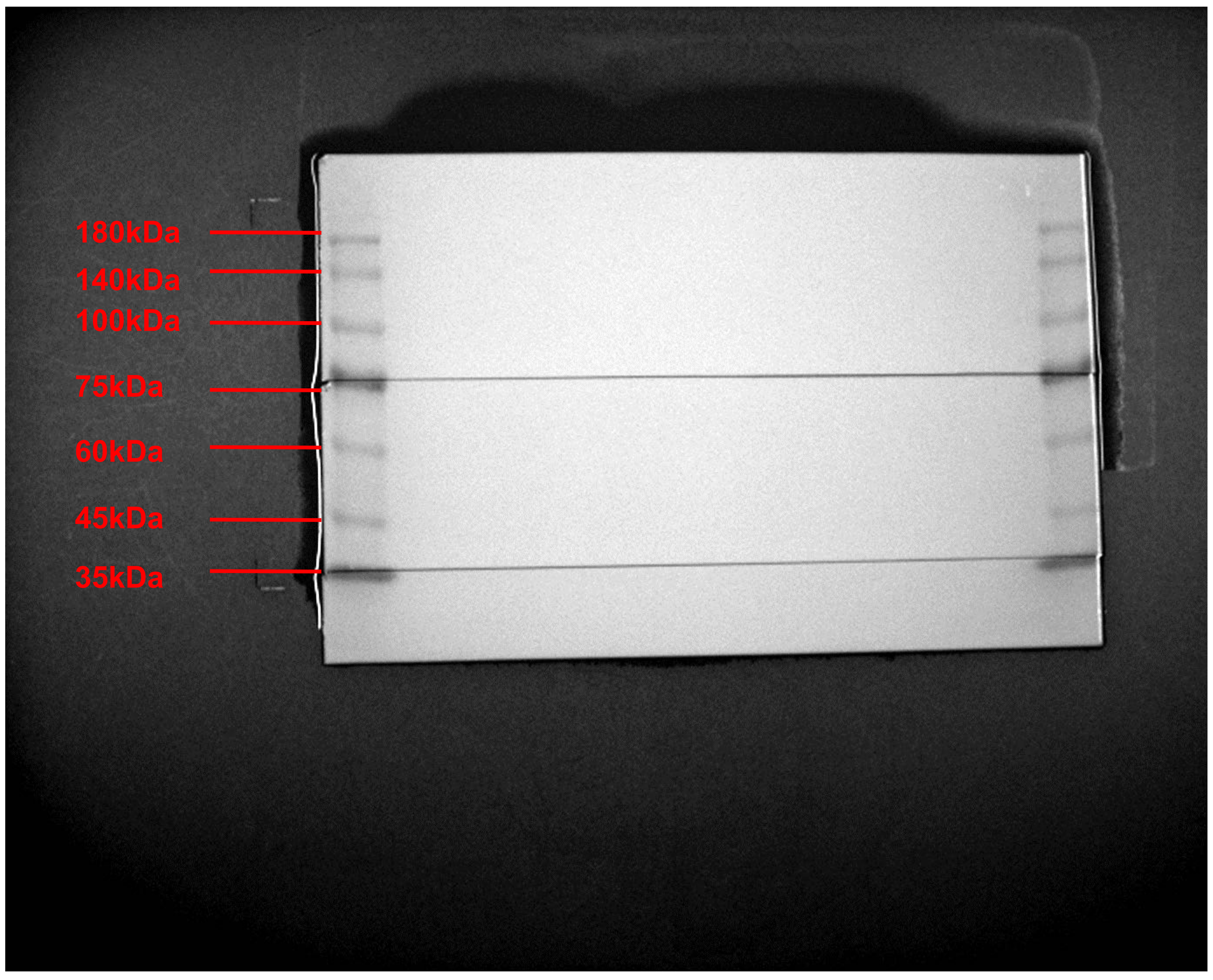

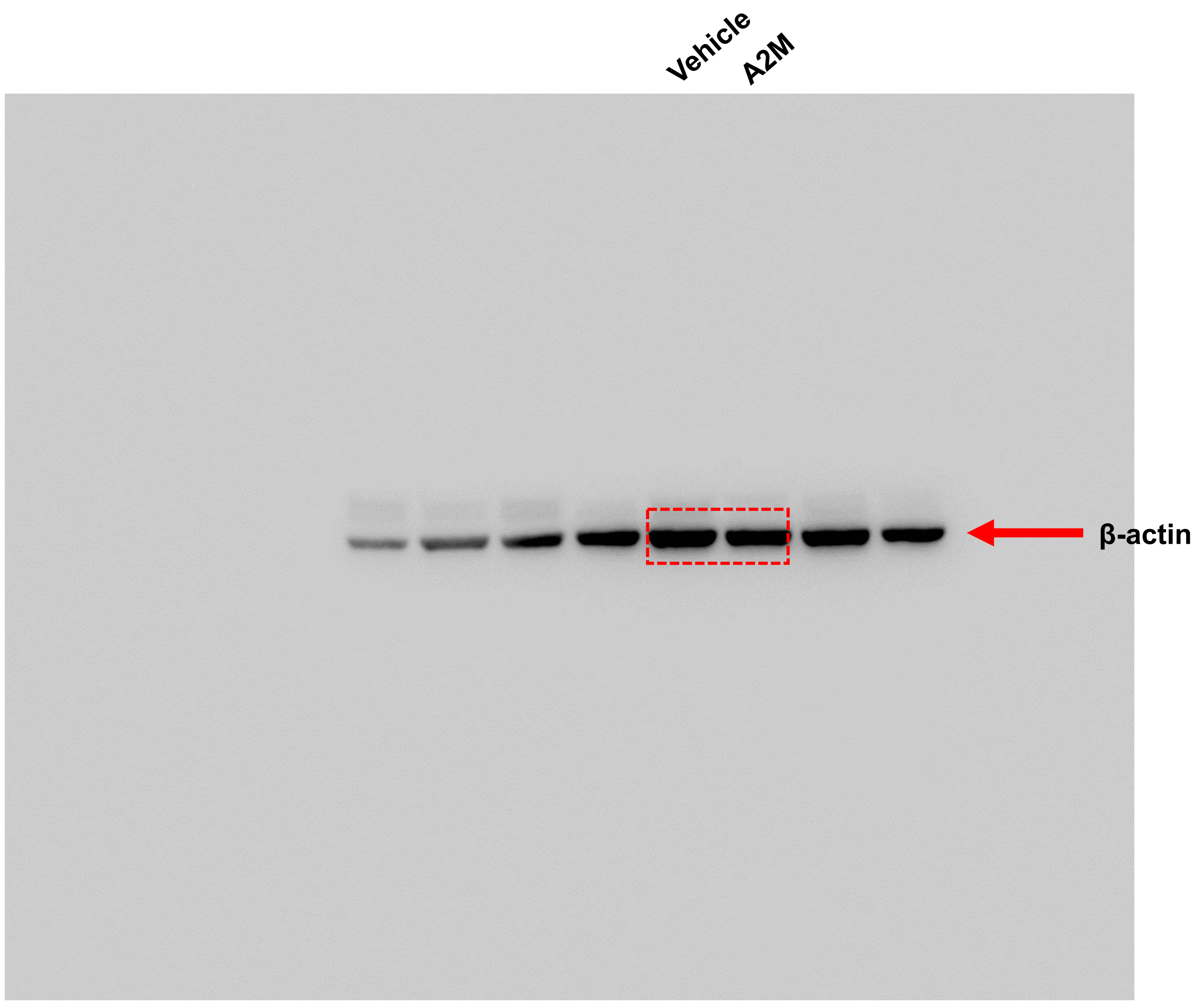

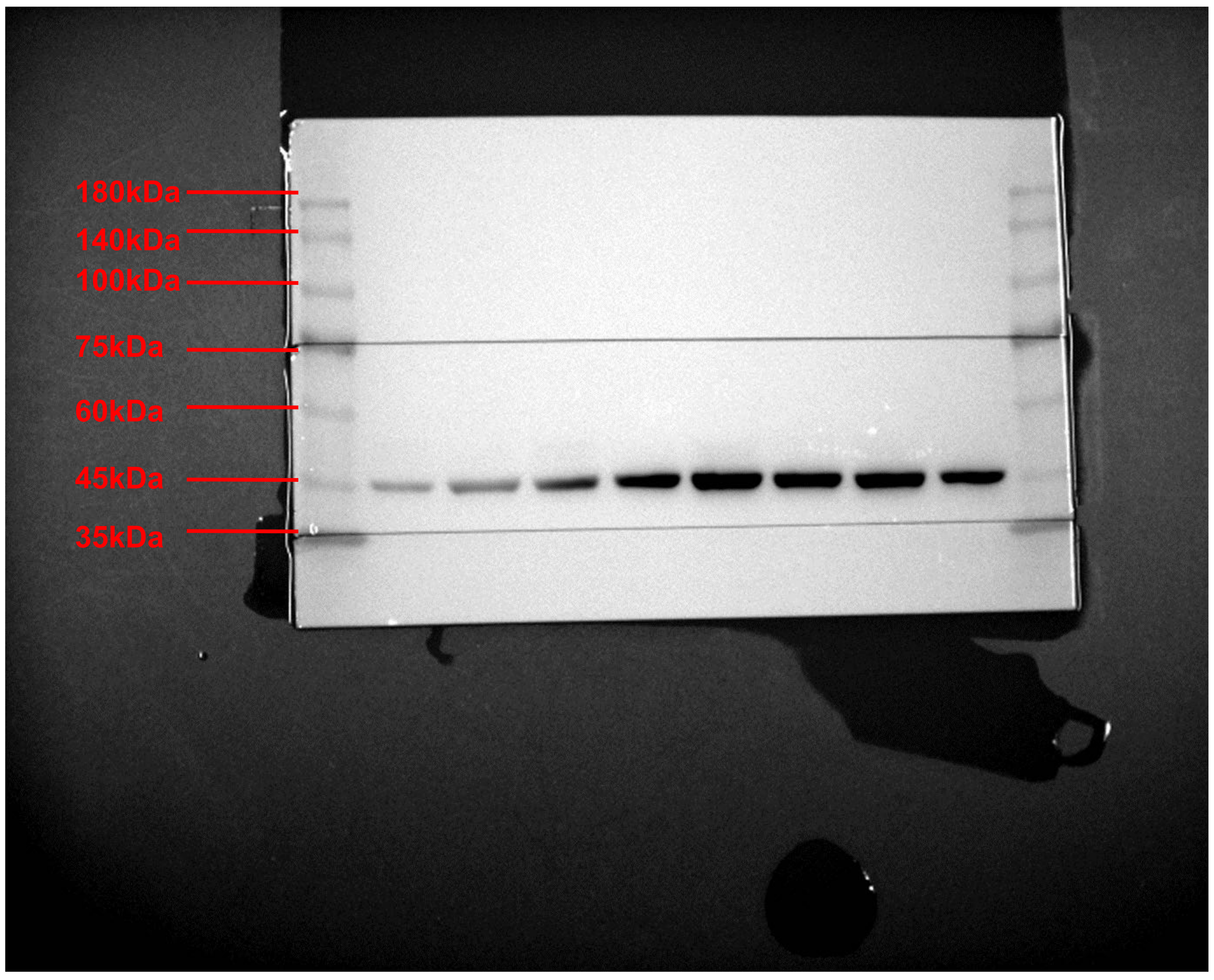

Blots for figure. Supplementary Fig. 6

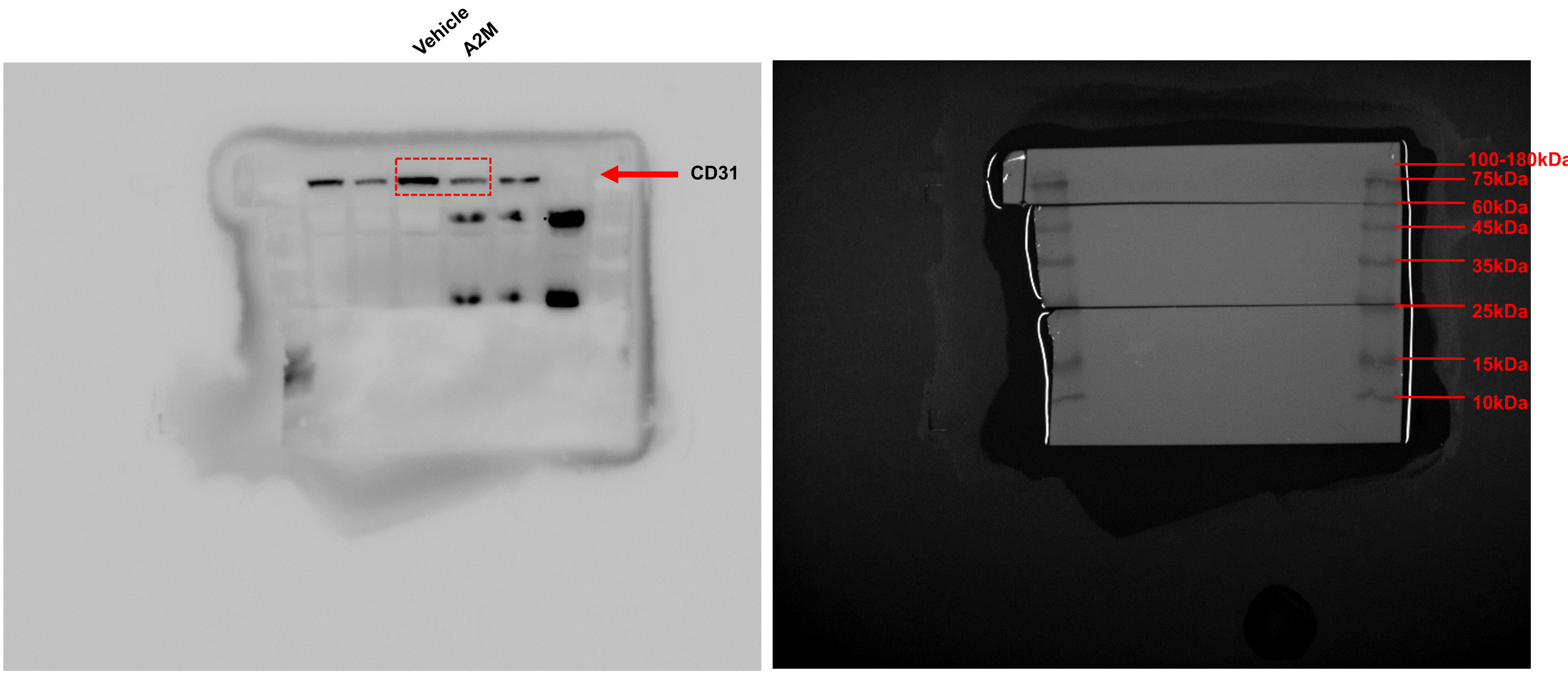

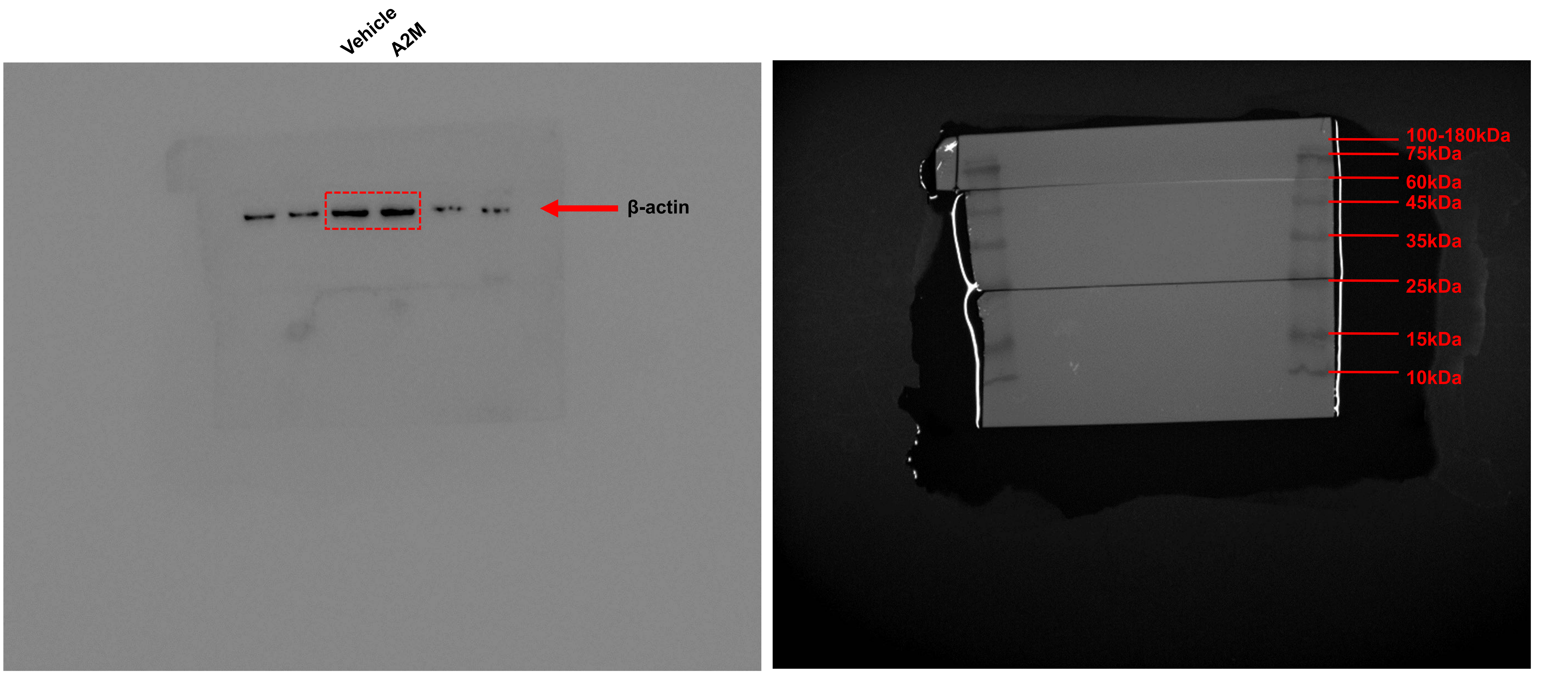

Blots for Supplementary Fig. 7

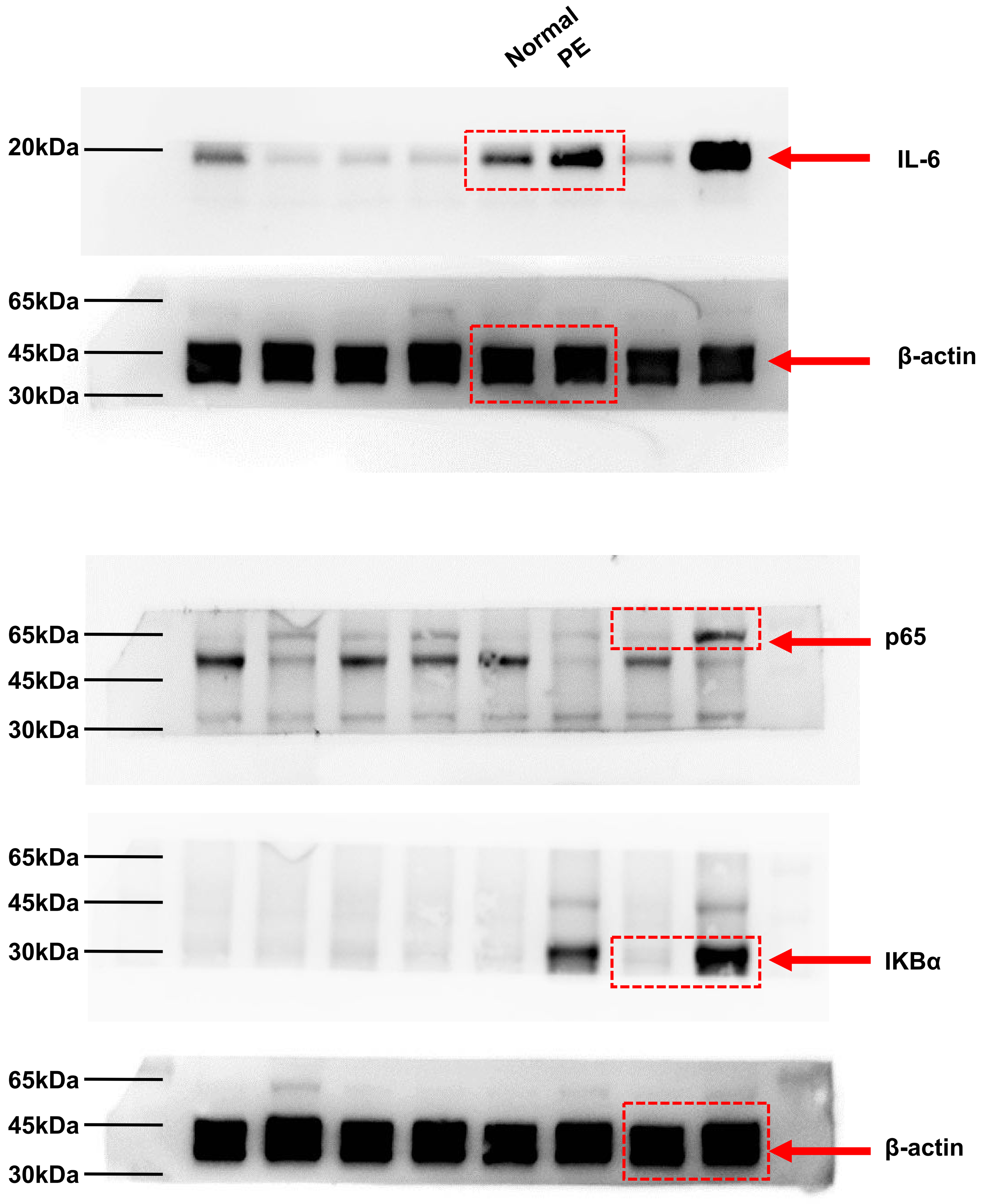

Supplement: Supplementary file 2 — Additional file 2. Western blot source data. [file 12916_2023_2807_MOESM2_ESM.pdf]
